# Reducible KAM Tori for the Degasperis–Procesi Equation

**DOI:** 10.1007/s00220-020-03788-z

**Published:** 2020-06-19

**Authors:** Roberto Feola, Filippo Giuliani, Michela Procesi

**Affiliations:** 1 UnivNantes, Nantes, France; 2 UPC, Barcelona, Spain; 3 RomaTRE, Rome, Italy

## Abstract

We develop KAM theory close to an elliptic fixed point for quasi-linear Hamiltonian perturbations of the dispersive Degasperis–Procesi equation on the circle. The overall strategy in KAM theory for quasi-linear PDEs is based on Nash–Moser nonlinear iteration, pseudo differential calculus and normal form techniques. In the present case the complicated *symplectic structure*, the *weak dispersive* effects of the linear flow and the presence of *strong resonant interactions* require a novel set of ideas. The main points are to exploit the integrability of the unperturbed equation, to look for special *wave packet* solutions and to perform a very careful algebraic analysis of the resonances. Our approach is quite general and can be applied also to other 1d integrable PDEs. We are confident for instance that the same strategy should work for the Camassa–Holm equation.

## Introduction and Main Result

In this paper we prove existence and stability of Cantor families of quasi-periodic, small amplitude, solutions for quasi-linear Hamiltonian perturbations of the Degasperis–Procesi (DP) equation

1.1 $$\begin{aligned} u_t-u_{x x t}+u_{xxx}-4 u_x-u u_{xxx}-3 u_x u_{xx}+4 u u_x +\mathcal {N}_8( u, u_x, u_{xx}, u_{xxx})=0 \end{aligned}$$

under periodic boundary conditions $x\in \mathbb {T}:=\mathbb {R}/2\pi \mathbb {Z}$, where

1.2 $$\begin{aligned} \mathcal {N}_8( u, u_x, u_{xx}, u_{xxx}) :=-(4-\partial _{xx}) \partial _x[(\partial _u f)( u)]\, , \end{aligned}$$

the “Hamiltonian density” *f* belongs to $C^{\infty }(\mathbb {R}, \mathbb {R})$ and is such that

1.3 $$\begin{aligned} f(u)=O(u^9), \end{aligned}$$

where $O(u^9)$ denotes a function with a zero of order at least nine at the origin. The Eq. (1.1) is a Hamiltonian PDE of the form $u_t=J\,\nabla H(u)$ where $\nabla H$ is the $L^2(\mathbb {T},\mathbb {R})$ gradient and the function

1.4 $$\begin{aligned} H(u)=\int \frac{u^2}{2}-\frac{u^3}{6}+f(u)\, dx{,} \quad J=(1-\partial _{xx})^{-1}(4-\partial _{xx})\partial _x\, \end{aligned}$$

is defined on the phase space $H_0^1(\mathbb {T}):=\left\{ u\in H^1(\mathbb {T}, \mathbb {R}) : \int _{\mathbb {T}} u\,dx=0\right\}$. The Eq. (1.1) for $f=0$ is the DP equation which was first proposed in [29] in the form

1.5 $$\begin{aligned} u_t+c_0 u_x+\gamma u_{xxx}-\alpha ^2 u_{xx t}= \left( -\frac{2 c_1}{\alpha ^2} u^2+c_2 (u_x^2+ u u_{xx})\right) _x{,} \end{aligned}$$

where $c_0,c_1,c_2,\gamma ,\alpha \in \mathbb {R}$, $\alpha \ne 0$. By applying Galilean boosts, translations and time rescaling to (1.5) one obtains Eq. (1.1) with $f=0$.

The DP equation can be regarded as a model for nonlinear shallow water dynamics and its asymptotic accuracy is the same as for the Camassa–Holm equation and a degree more than the KdV equation [23]. There is a rather large literature on this equation starting form the paper [28] in which the *complete integrability* is proved. The local and global well-posedness, for instance, have been extensively studied as well as existence of wave breaking phenomena (peakons, N-peakons solutions). Without trying to be exhaustive we quote [18, 20–22, 48, 54] and we refer to [32] and references therein for more literature about Degasperis–Procesi equation.

Actually many of these results (notably the wave breaking) are studied in the *dispersionless* case, which corresponds to (1.1) with $f=0$ and $u\rightsquigarrow u+1$. In the present paper the presence of the dispersive terms $-4 u_{x}+ u_{xxx}$ is fundamental. Our main purpose is to prove existence of quasi-periodic solutions in high Sobolev regularity by following a KAM approach. In this setting a *quasi*-periodic solution with $\nu \in \mathbb {N}$ frequencies is defined by an embedding

1.6 $$\begin{aligned} \mathbb {T}^{\nu }\ni {\varphi }\mapsto U({\varphi },x) \in H_0^1(\mathbb {T}, \mathbb {R}) \end{aligned}$$

and a frequency vector $\omega \in \mathbb {R}^{\nu }$, with rationally independent entries, such that $u(t,x)=U(\omega t,x)$ is a solution of (1.1) and $U({\varphi },x)\in H^p(\mathbb {T}^{\nu +1},\mathbb {R})$ for some *p* sufficiently large.

Notice that, in a neighbourhood of $u=0$, (1.1) can be seen as a perturbation of the linear PDE

1.7 $$\begin{aligned} v_t-v_{x x t}+v_{xxx}-4 v_x=0{,} \end{aligned}$$

whose bounded solutions have the form

1.8 $$\begin{aligned} v(t, x)=\sum _{j\in \mathbb {Z}} v_j\,e^{\mathrm {i}({\lambda }(j) t+j x)}{,}\qquad {\lambda }(j):=j\,\frac{4+j^2}{1+j^2}=j+\frac{3 j}{1+j^2}, \qquad j\in \mathbb {Z}{,} \end{aligned}$$

where $j\mapsto \lambda (j)$ is the *linear* dispersion law. It is easily seen that all solutions of (1.7) with compact Fourier support are periodic, but with period depending on the support. In this context it is natural to investigate whether Eq. (1.1) has periodic or quasi-periodic solutions *close to* to small amplitude linear solutions (1.8). We remark that, since the solutions of (1.8) are all periodic, the existence of quasi-periodic solutions, if any, strongly relies on the presence of the quadratic nonlinearity in (1.1).

In the present paper we construct quasi-periodic solutions mainly supported in Fourier space at $\nu \ge 2$ distinct *tangential sites*

1.9 $$\begin{aligned} S^+:=\{\overline{\jmath }_1, \ldots , \overline{\jmath }_{\nu } \}, \quad S:=S^{+}\cup (-S^+), \quad \overline{\jmath }_i\in \mathbb {N}\setminus \{ 0\}, \quad \forall i=1,\ldots , \nu {,} \end{aligned}$$

where, without loss of generality, we shall always assume that $\overline{\jmath }_1=\max _{i=1,\ldots , \nu }\overline{\jmath }_i$. We denote by

1.10 $$\begin{aligned} \overline{\omega }:=\left( \frac{\overline{\jmath }_1(4+\overline{\jmath }_1^2)}{1+\overline{\jmath }_1^2},\ldots , \frac{\overline{\jmath }_{\nu }(4+\overline{\jmath }_{\nu }^2)}{1+\overline{\jmath }_{\nu }^2}\right) \in \mathbb {Q}^{\nu } \end{aligned}$$

the linear frequencies of oscillations related to the tangential sites. More precisely our solutions will have the form

1.11 $$\begin{aligned} u(t, x; \xi )=2\sum _{i=1}^\nu \sqrt{\xi _i}\,\cos (\omega _i t+\overline{\jmath }_i x) +o(\sqrt{|\xi |}), \quad \omega =\overline{\omega } +O(|\xi |), \end{aligned}$$

where $o(\sqrt{|\xi |})$ is meant in the $H^{s}$-topology with *s* large. It is well know that in looking for quasi-periodic solutions “small divisors” problems arise. To overcome such problems we shall require that $S^+$ satisfies a *wave packet* condition and that the *unperturbed amplitudes*$\xi$ belong to an appropriate Cantor-like set of positive measure.

The following definition quantifies the *wave packet* condition.

### Definition 1.1

For $\mathtt {r}\in (0, 1)$, we say that a set of natural numbers $S^+=\{\overline{\jmath }_1, \ldots , \overline{\jmath }_{\nu }\}$ is in $\mathcal {V}(\mathtt {r})$ if

1.12 $$\begin{aligned}&\min _{i=1, \ldots , \nu } \overline{\jmath }_i >\frac{1}{\mathtt {r}} \qquad \text{ and } \qquad \left|\frac{\overline{\jmath }_i}{\overline{\jmath }_1} -1 \right|\le \mathtt {r}\,; \end{aligned}$$

1.13 $$\begin{aligned}&\sum _{i=1}^{\nu } \frac{\overline{\jmath }_i}{1+\overline{\jmath }_i^2}\,\ell _i\ne 0{,} \quad \forall \ell \in \mathbb {Z}^{\nu }:\quad |\ell |= 4{.} \end{aligned}$$

Denoting by $B(0,\varrho )$ the ball centred at the origin of $\mathbb {R}^{\nu }$ of radius $\varrho >0$, our result can be stated as follows.

### Theorem 1

Let $\nu \in \mathbb {N}$, $\nu \ge 2$, and consider $f\in C^{\infty }(\mathbb {R},\mathbb {R})$ satisfying (1.3). There exists a constant $\mathtt {r}_0>0$ such that, for any choice of $S^+$ in $\mathcal {V}(\mathtt {r})$, with $0<\mathtt {r}\le \mathtt {r}_0$, there exist $s\gg 1$, $0<\varrho \ll 1$ and a positive measure Cantor-like set $\mathfrak {A}\subseteq B(0,\varrho )$ such that the following holds. For any $\xi \in \mathfrak {A}$, the Eq. (1.1) possesses a small amplitude quasi-periodic solution $u(t,x; \xi )=U(\omega t,x; \xi )$ of the form (1.11) where $U({\varphi },x)\in H^{s}(\mathbb {T}^{\nu +1},\mathbb {R})$ and $\omega :=\omega (\xi )\in \mathbb {R}^{\nu }$ is a diophantine frequency vector. Moreover for $0<\varepsilon \le \sqrt{\varrho }$, the set $\mathfrak A$ has asymptotically full relative measure in $[\varepsilon ^2,2\varepsilon ^2]^\nu$.

Moreover we have the following stability result.

### Theorem 2

(Linear stability). The quasi-periodic solutions (1.11) $u(t, x)=U(\omega t, x)$ of Eq. (1.1) are *linearly stable* and *reducible* in the following sense. Consider Eq. (1.1) linearized at the embedded torus $U({\varphi },x)$, then the corresponding operator has purely imaginary spectrum and there exists a change of variables $H^s(\mathbb {T}, \mathbb {R}) \rightarrow H^s(\mathbb {T}, \mathbb {R})$, quasi periodic in time with frequency $\omega$, which diagonalizes it in the directions normal to the torus. As a consequence the Cauchy problem of the linearized equation is stable, i.e. the Sobolev norms are uniformly bounded in *t*.

Theorems 1, 2 are formulated in the typical style of results on reducible KAM tori for PDEs. For the proof we use the overall strategy of [4], which however has to be substantially developed to deal with (1.1). Let us briefly explain the main new issues.

- The dispersion law is *asymptotically linear* as for the Klein–Gordon equation, studied for instance in [6, 7]. As explained in those papers, the fact that the dispersive effects are very weak (essentially time and space play the same role) creates a number of difficulties even in the study of KAM theory for semi-linear PDEs. Of course, since (1.1) is quasi-linear, there are additional serious difficulties coming from the strong perturbative effects of the nonlinearity.
- The DP equation is *resonant* at zero and does not depend on any *external* parameters. This is a fundamental difference w.r.t. the Klein–Gordon equation, where one modulates the mass in order to avoid resonances. Moreover the DP has non-trivial resonances already at order four (see Sect. 1.3), differently from the previous KAM results for quasi-linear PDEs. As a further difficulty the algebraic structure of the resonances is quite complicated. In order to avoid the inherent problems we rely on the presence of “many” (precisely *eight*) approximate constants of motion of (1.1) coming from the integrable structure of the DP equation. Dealing with the problems related to resonances is the core of this paper and requires a set of new ideas and a careful analysis.
- The very strong restriction of the tangential sites $S^{+}$ is exploited several times to simplify the problems arising from the rational and asymptotically linear dispersion law. Physically we are looking for solutions mainly supported in Fourier space on modes which are *relatively close* to each other. It seems reasonable that such condition could be weakened, but it is not clear to us how to deal with the technical difficulties which would arise.
- As in other resonant cases, the diophantine constant $\gamma$ is related to the size of the solution one is looking for (see (1.11)). Moreover, due to the linear dispersion law, we are forced to impose very “weak” non-degeneracy conditions on the linear frequencies of oscillations. As a consequence we need a refined bifurcation analysis in order to find a very good first approximate solution and fulfil the smallness conditions required for the Nash–Moser scheme.

Some comments on Eq. (1.1) and on Theorems 1, 2 are in order.

### The unperturbed DP equation

We look at (1.1) as a perturbation of the linear equation (1.7), in order to fit the typical perturbative setting of KAM for PDEs , we refer to Sect. 1.1 for more details.

Actually, since the Degasperis–Procesi equation is completely integrable (see [28]) it would be very natural to try to construct solutions of (1.1) which bifurcate from quasi-periodic solutions of the unperturbed DP equation

1.14 $$\begin{aligned} u_t-u_{x x t}+u_{xxx}-4 u_x-u u_{xxx}-3 u_x u_{xx}+4 u u_x=0, \end{aligned}$$

which corresponds to (1.1) with $f=0$. Indeed, near zero, the (1.1) can be seen *also* as a perturbation of (1.14). Unfortunately even though algebro-geometric *finite-gap* solutions have been already constructed in literature for the DP equation (see [42]) it is not clear to us whether they are real quasi-periodic solutions in the sense of (1.6). Of course if one were able to bifurcate from *finite-gap* solutions of (1.14) then it would be possible to prove existence of *large* quasi-periodic solutions, by requiring that *f* is small. Such a strategy has been followed successfully for the KdV and cubic NLS equation on the circle. Actually for those equations one can prove the existence of Birkhoff coordinates [41, 43] (the Cartesian version of action-angle variables), which trivialize the dynamics (in the sense that the solutions turn out to be all periodic, quasi-periodic or almost periodic) and provide a fundamental tool for investigating the dynamical consequences of small perturbative effects, also far from the origin, see [14].

For 1d integrable PDEs one would expect this to be the typical scenario at least in a neighborhood of zero, see [5, 46]; however, as far as we know, up to now such results are available only for the KdV, the NLS and the Toda system. Theorem 1 provides, again as far as we know, the first existence result of quasi-periodic solutions, in the sense of (1.6), for (1.14).

It would be interesting to apply our KAM approach to the Camassa–Holm equation, which is a well-known integrable PDE with an asymptotically linear dispersion law, but with a different symplectic structure. Even though we have not performed the computations, we expect to be able to prove the equivalent of Theorems 1, 2 also for this equation. We remark that in this case, the finite gap solutions are known to be quasi-periodic tori, see [20].

One could start by comparing them with the solutions predicted by our method and then possibly develop KAM theory close to large finite gap solutions.

### Approximate constants of motion of (1.1)

Even though we do not fully exploit the integrability of (1.14) it is fundamental for us that (the non integrable) (1.1) has at least *eight* approximate constants of motion (up to an error of order $O(u^9)$). It is interesting to notice that, as shown in [29], no other equation with the same dispersion law, and the same symplectic structure, has *eight* approximate conserved quantities. This means that in (1.1) we cannot consider any quadratic nonlinearity, but we really need the DP structure.

The request of the presence of such approximate conserved quantities it is not only a technical matter. In order to implement a Nash/Moser-KAM algorithm one looks for a family of approximately invariant tori of (1.1) (with a sufficiently good approximation) such that the dynamics on the tori is integrable and non-degenerate, while the dynamics normal to the torus is non-degenerate at the linear level and satisfies the Melnikov conditions. If there are external parameters modulating the linear frequencies, then we can consider as approximate solutions the linear ones. Otherwise the modulation must come from the initial data and, hopefully, this can be achieved by means of Birkhoff normal form (BNF), see for instance [4, 39]. In this case, where the the dispersion law in (1.8) is a rational number and is asymptotically linear, such procedure is very difficult. One has to explicitly compute some potentially dangerous resonant terms in the Hamiltonian and show that they vanish. This is the same type of computations which have been done for water waves, see Craig–Worfolk [27] where the authors verify (by computing them) the vanishing of the coefficients of fourth order resonant interactions, the so called *Benjamin–Feir resonances*. In our case we have to deal with higher order resonances (up to *eight*), so this would be computationally extremely heavy. Our approach is to use the approximate constant of motions. This will be explained more in detail in Sect. 1.3. Once we have constructed the approximate invariant tori we have to impose the non-degeneracy and Melnikov conditions. Differently form the KdV case, this will not be possible for any choice of the tangential set, and it is where we will use the condition $S^+\in \mathcal {V}(\mathtt {r})$, see Definition 1.1.

#### Linear stability

The linear stability result of Theorem 2 is of course a relevant dynamical information in the study of evolutionary PDEs, but it is also the consequence of a fundamental ingredient of our proof: the reducibility of the linearized equation at any quasi-periodic approximate solution. Reducibility for the Degasperis–Procesi equation linearized at a quasi-periodic function has been obtained in [33], under some appropriate diophantine conditions on the frequencies. Unfortunately, due to the resonances, our case does not fit such hypotheses, and a major point will be to overcome this difficulty. Here we shall use such result (appropriately adapted) inside a nonlinear algorithm to prove the existence of quasi-periodic solutions. This is a classical feature of the literature of KAM theory.

### Some literature

Proving existence and stability for quasi-periodic solutions for PDEs close to an elliptic fixed point is a natural extension of the classical KAM theory for lower dimensional tori [51]. The first results in this direction were for model PDEs on an interval with no derivatives in the nonlinearity and with either Dirichlet, [44, 47, 51, 53] or periodic, [16, 19, 26], boundary conditions. For extension of KAM theory to higher spatial dimension we mention [8, 11, 17, 25, 30, 34, 52]. While KAM methods for constructing quasi-periodic solutions for PDEs on the circle with no derivatives in the nonlinearity are by now well established, generalizing to cases with derivatives is in general not at all trivial, even in the semi-linear cases (where the derivatives in the nonlinearity are of lower order w.r.t. the linear terms). We mention [45] for the KdV, [49] for the derivative NLS, and [6, 7] for the derivative NLW. Recently an innovative strategy was proposed, [3, 4] to deal with quasi-linear and fully nonlinear PDEs on the circle. This approach was first developed for the KdV equation but can be applied to many equations of interest in hydrodynamics, such as NLS, [37, 38] Kirchhoff [50] or directly the water wave equation [2, 15]. While these methods were first thought for PDEs on the circle, of course a very interesting point is the generalization to higher dimensions.

Equation (1.1) is a quasi-linear PDE on the circle and in our study we shall follow the general strategy of [4], extended and adapted to our case. Let us briefly explain the point of view of [4], referring also to [2] for more details.

### The general strategy

We describe the strategy to prove existence and linear stability for small, reducible quasi-periodic solutions of completely resonant quasi-linear PDEs.

(*i*): The starting point is a Nash–Moser theorem of hypothetical conjugation following [9]. The strategy is to construct quadratically convergent sequence of families of approximately invariant (isotropic) tori. Such construction is based on *tame* estimates on the inverse of the operator associated to the Eq. (1.1) linearized at an approximate torus and restricted to the normal direction. This is proved by exploiting the Hamiltonian structure and exhibiting symplectic variables adapted to each approximate invariant torus, which essentially decouple the linearized dynamics. Then the bounds on the inverse are achieved by removing all the “bad” values of the parameters. We mention also [24] for a parallel strategy which does not rely on the Hamiltonian structure.
(*ii*): To construct the sequence of item (*i*) we need a good starting point, i.e. a first family of approximately invariant tori parametrized by real vectors $\xi \in \mathbb {R}^{\nu }$.

As explained before this is achieved by BNF techniques. In particular, in the quasi-linear context, it is convenient to perform a *Weak* BNF, i.e. to exhibit a change of variables, close to the identity up to a finite rank operator, such that the following holds. The Hamiltonian *H* transforms to $H_\mathrm{Birk}+ R$ where *R* is a small remainder, and

1. The finite dimensional subspace $U_S:=\{u_j=0,\;\forall j\notin S\}$ is invariant for $H_\mathrm{Birk}$;
2. The Hamiltonian restricted to $U_S$ is integrable and non-degenerate in the sense that the “frequency-to-amplitude” map is invertible.

In order to describe in a simpler way the dynamics in a neighborhood of $U_S$ it is convenient to define *action-angle* variables. This allows to distinguish the tangential and normal dynamics to the approximately invariant tori.

We remark that, for semi-linear PDEs, typically one performs a stronger BNF preliminary step, in order to “normalize” also the linearized dynamics normal to the torus, i.e. the terms in the Hamiltonian which are quadratic in the normal directions. In this case the Birkhoff map is close to the identity up to a bounded operator (at most one-smoothing), see for instance [47, 51]. Compared to the latter approach, the weak procedure has the disadvantage that the normal form depends on the angles; on the other hand we do not have to address well-posedness issues, since these changes of coordinates are time-one flow maps of an ODE. Note that the recent papers, [10, 35, 36] directly study the full Birkhoff normal form for quasi-linear PDES.

(*iii*): The third key point is to study the invertibility of the linearized operator restricted to the normal directions. Thanks to the very “mild” conjugation procedure of item (*ii*) (with a map = identity+finite rank) it turns out that such linear operator is *pseudo differential* (with non constant coefficients) up to a finite rank remainder. This is the most important reason for adopting the weak procedure described in (*ii*).

The invertibility of the linearized operator, with appropriate tame estimates, is based on a *reducibility* argument which is divided into two parts:

- A reduction in decreasing order procedure which conjugates the linearized operator to a pseudo differential one with constant coefficients up to a remainder which is a bounded/regularizing term i.e. maps $H^{s}(\mathbb {T}, \mathbb {R})$ to $H^{s+\rho }(\mathbb {T}, \mathbb {R})$, $\rho \ge 0$. The choice of $\rho$ depends of course on the problem one is studying;
- A quadratic KAM scheme (for *bounded* operators) which completely diagonalizes the bounded/smoothing remainder of the previous step.

We want to point out the following:

- The step (a) strongly relies on the pseudo differential structure of the operator;
- The normal form contains angle-dependent terms and some of them turn out to be not perturbative for the KAM scheme (*b*). The conjugation to constant coefficients of such terms relies on purely algebraic arguments. We refer to this procedure as *linear Birkhoff normal form*;
- As a consequence of having applied the weak and the linear Birkhoff procedure, the normal form around the approximately invariant tori has constant coefficients also in the normal directions.

In order to perform the diagonalization procedure of step (b) one needs the *second Melnikov conditions*, which essentially amount to requiring that the operator has simple eigenvalues with a lower bound on the differences. Once one has diagonalized the operator, the bounds on the inverse follow trivially from lower bounds on the eigenvalues, i.e. *first Melnikov conditions*.

(*iv*): In the scheme above, at each step we have removed some bad values of the parameters $\xi$ where the Melnikov conditions do not hold. Hence the last (but not least) step is to prove that at the end of the procedure one has still a positive measure set of parameters. Note that often it is more convenient to express such conditions in terms of the frequency of the quasi-periodic solution. This can be done thanks to the invertibility of the frequency-to-amplitude map.

### Main novelties and scheme of the proof

We describe the structure of the paper following Sect. 1.2, and with particular attention to the main novelties.

In Sect. 2 we introduce the Hamiltonian formalism for the DP equation and the functional spaces on which we shall work.

In Sect. 3 we perform the weak Birkhoff normal form explained in item (ii) of the previous section. The result is stated in Proposition 3.2. In order to reach a sufficiently good first approximate solution we need to perform 6-BNF steps. As is well-known, at the *n*-th step of this procedure one has to take into account the denominators (recall (1.8))

1.15 $$\begin{aligned} \lambda (j_1)+\cdots +\lambda (j_{n+2}). \end{aligned}$$

We say that a $(n+2)$-uple of integer indices $(j_1, \ldots , j_{n+2})$ is a resonance, and hence may appear in $H_\mathrm{Birk}$, if (1.15)$=0$ and the momentum condition holds, namely $\sum _{i=1}^{n+2} j_i=0$. We say that a resonance is *trivial* if it has the form $(i, -i, j, -j, \ldots )$ so that the corresponding monomial is integrable.

As mentioned before a major difficulty comes from the fact that the DP equation has many non-trivial resonances (already at order four) and in principle there is no reason why the Birkhoff Hamiltonian restricted to $U_S$ should be integrable. By the fact that the Hamiltonian density *f* is of order $O(u^9)$ the perturbation does not affect the leading terms of the Birkhoff Hamiltonian and we can exploit the integrability of the DP equation. Indeed the same Birkhoff transformation should normalize simultaneously all the commuting Hamiltonians. This means that a resonant monomial contributes to $H_\mathrm{Birk}$ if and only if it is resonant for all the constants of motion. This was proved in detail in [32] at the level of formal power series. Here we adapt this result to the Eq. (1.1) which is only approximately integrable (close to the origin) and we reformulate it in a way better suited to the weak Birkhoff normal form context, see Proposition 3.6.

Once we have shown that the $H_\mathrm{Birk}$-dynamics restricted to $U_S$ is integrable, in Sect. 4, we prove that it is non-degenerate, i.e. that the frequency to amplitude map is a diffeomorphism. We have a very explicit description of this map and hence this step amounts to proving that the matrix $\mathbb {A}$ in (4.6) (which depends only on $S^+$) has determinant bounded away from zero (the so-called twist condition), see Lemma 4.1. A big difference with [4] is that, in our case, the determinant of $\mathbb {A}$ is a rational function of several variables $\overline{\jmath }_i$ that could accumulate to zero as $|\overline{\jmath }_i |\rightarrow \infty$. By imposing the wave packet condition we restrict the study of its asymptotic behaviour to regions in which it behaves like a one variable function. Then we use continuity arguments to guarantee the invertibility of $\mathbb {A}$ for every choice of $S^+\in \mathcal {V}({\mathtt {r}})$ (see Definition 1.1) for $\mathtt {r}$ small enough. Outside $\mathcal {V}({\mathtt {r}})$ the proof of lower bounds for $\det \mathbb {A}$ should rely on purely algebraic arguments and not on perturbative ones.

In Sect. 5 we introduce the Nash Moser hypothetical conjugation theorem (see Theorem 5.4) and in Sect. 6 we explain how to prove the invertibility of the linearized operator at an approximate solution by only studying it in the normal direction. Since there is no difference with [4] we only give a synopsis.

In Sects. 7 and 7.3 we prove the Theorems 7.1 and 7.13 which provide the reducibility of the linearized operator following item (iii) of Sect. 1.2. As we already mentioned, in [33] we provide a reducibility result for the DP equation (1.1) linearized at sufficiently small quasi-periodic functions under appropriate diophantine conditions on the frequencies . Unfortunately in our case the diophantine constant $\gamma$ is related to the size of the approximate solutions (see (5.3)) and then the smallness and diophantine conditions above cannot be met.

In [4] this issue appears only in the step (*b*) of the strategy, where it is solved by the linear Birkhoff normal form method. A first difficulty in our case is that this problem appears also in step (*a*). So that we first need to perform some preliminary steps (see Sect. 7.1), more precisely we need changes of coordinates, preserving the pseudo differential structure, that conjugate the leading order of the linearized operator to a diagonal one plus a correction, which is unbounded but perturbative in the sense of [33]. In such steps the provided changes of coordinates are similar in structure to those of step (*a*) but they are proved to be well-defined not by using perturbative arguments, but by algebraic computations involving the Birkhoff resonances (see Lemma A.1). These difficulties appear also for the quasi-linear generalized KdV [39], but here we have several further problems due to the complexity of the symplectic structure of the DP equation. The first step, removing terms of order $\varepsilon$, is straightforward. Already at the second step we encounter the difficulties arising form the presence of non-trivial resonances of order 4, and a priori there is no reason why the normal form should be integrable. Here it does not appear simple to apply the strategy of the weak BNF, using the constants of motion. On the other hand, computing the normal form explicitly by hand, as done in [39], is unmanageable. To bypass this problem we take a different point of view, based on an *a posteriori identification argument* of normal forms. More precisely in Theorem 7.9 we prove that the normal form obtained after the weak BNF, the preliminary steps and the linear BNF coincides with the one that we would obtain by performing the full *formal* BNF and then projecting on the quadratic terms in the normal variables. This result strongly relies on the fact that all the resonances contributing to the formal normal form are trivial. A similar identification argument has been used, for instance, in [12, 13].

A further point is that, due to the rational dispersion law $\lambda (j)$, it is possible that a *denominator* in the linear BNF is not zero but is still uncontrollably small. In the third step, in order to deal with this problem we need to take into account in the unperturbed Hamiltonian also the integrable terms of order $\varepsilon ^2$ coming from the previous steps of linear BNF. For this reason it is important to know the exact expression of the main order of the correction at the eigenvalues given by the perturbation, see for instance (5.5). This is also needed in the KAM scheme (*b*), in order to impose the second Melnikov conditions. Computing these corrections by hand would be a very difficult task, but this comes for free from Theorem 7.9.

In the first part of Sect. 8 we show the convergence of the Nash–Moser algorithm (see Theorem 8.1), which requires the ratio between the size of $R=H-H_\mathrm{Birk}$ and $\gamma ^{7/2}$ to be small (see the smallness condition (8.5)); in the second part we prove that the set of “bad” parameters, i.e. the frequencies which do not meet the first and second Melnikov conditions, has small measure (see (8.25), note that such sets are indexed by three parameters $\ell ,j,k$).

In Lemma 8.4 we provide the measure of the single bad set. Here we use the algebraic arguments provided by Lemma A.1, which guarantees the non-degeneracy of the leading terms of the small divisors. In Sect. 8.1.2 we deal with the summability of the bad sets in *j*, *k* for fixed $\ell$.

The key difficulty is that the spectral gap $\lambda (j)-\lambda (k)$ is asymptotically constant, hence there is a bad separation property of the eigenvalues. The same occurs for the wave equation [6, 7]. Due to the asymptotically constant spectral gap, these sets are infinitely many. Then the key ingredient is to show that for *j*, *k* sufficiently large the second Melnikov conditions are implied by the first ones. This is possible provided that we consider two different diophantine constants. More precisely we have to impose second order Melnikov conditions with $\gamma ^{3/2}$ (see (8.6)), which is clearly much smaller than $\gamma$. This is why we have to perform many steps of Birkhoff normal form in order to obtain a very good first approximate solution.

We point out that, differently from [2], our Melnikov conditions do not imply a loss of regularity in space. In [2] this loss is acceptable, since in the regularization step ((*a*) p. 5) the diagonalization is performed up to a very smoothing remainder. In this procedure it is fundamental that the diophantine constant $\gamma$ is independent of the size of the solution. Of course in our case this is not true and thus in the regularization step we end up with a remainder of order $-1$, and then in the measure estimates we put some extra efforts to prove second Melnikov conditions without loss of regularity.

## Functional Setting

**Hamiltonian formalism of the Degasperis–Procesi equation** For any *u*, *v* in the space

$$\begin{aligned} H_0^1(\mathbb {T}):=\left\{ u\in L^2(\mathbb {T}, \mathbb {R}) : \int _{\mathbb {T}} u\,dx=0\right\} \end{aligned}$$

we define the non-degenerate symplectic form

2.1 $$\begin{aligned} \Omega (u, v):=\int _{\mathbb {T}} (J^{-1} u)\,v\,dx=(J^{-1}u,v)_{L^{2}} \end{aligned}$$

where *J* is defined in (1.4) and $(\cdot ,\cdot )_{L^{2}}$ is the $L^{2}(\mathbb {T}, \mathbb {R})$ scalar product. To any $C^{1}$ function $H : H_0^{1}(\mathbb {T})\rightarrow \mathbb {R}$ we associate a vector field $X_{H}$ by requiring

$$\begin{aligned} dH(u)[h]=(\nabla H(u),h)_{L^{2}}=\Omega (X_{H}(u),h), \quad \forall \, u,h\in H_0^{1}(\mathbb {T}){.} \end{aligned}$$

The Hamiltonian vector field $X_{H}$ is uniquely determined since the symplectic form $\Omega$ in (2.1) is non-degenerate, in particular $X_{H}(u)=J\nabla H(u)$. The *Poisson bracket* between two $C^{1}$ functions $F,G : H_0^{1}(\mathbb {T})\rightarrow \mathbb {R}$ is

2.2 $$\begin{aligned} \{F,G\}:=\Omega (X_{F},X_{G})=(\nabla F, J\nabla G)_{L^2}{.} \end{aligned}$$

In this way

2.3 $$\begin{aligned} X_{\{F,G\}}=[X_{F},X_{G}]{,} \quad \mathrm{where}\quad [X,Y]:=dX [Y]-dY [X]{.} \end{aligned}$$

Finally, given a Hamiltonian *H* we define its *adjoint action* as the operator

2.4 $$\begin{aligned} \mathrm {ad}_{H}[\cdot ]:=\{H, \cdot \}{.} \end{aligned}$$

Consider now two Hamiltonians *H*, *G* and let $\Phi _{G}$ be the time-1 flow map of the vector field $X_{G}$. Then we have (formally)

2.5 $$\begin{aligned} H\circ \Phi _{G}= & {} \sum _{k\ge 0}\frac{(-1)^{k}}{k!}\mathrm {ad}_{G}^{k}[H]{,} \qquad H\circ \Phi ^{-1}_{G}=\sum _{k\ge 0}\frac{1}{k!}\mathrm {ad}_{G}^{k}[H]{,}\nonumber \\ \mathrm {ad}_{G}^{k}[H]:= & {} \mathrm {ad}_{G}\big [\mathrm {ad}_{G}^{k-1}[H]\big ]{,} \end{aligned}$$

where $\mathrm {ad}_{G}^{0}:=\mathrm {I}$ is the identity map.

**Functional space** We consider functions $u(\varphi , x)$ defined on $\mathbb {T}^{\nu }\times \mathbb {T}$. Passing to the Fourier representation

2.6 $$\begin{aligned} u(\varphi , x)=\sum _{j\in \mathbb {Z}} u_{j}(\varphi )\,e^{\mathrm {i} j x}=\sum _{\ell \in \mathbb {Z}^{\nu }, j \in \mathbb {Z}} u_{\ell j} \,e^{\mathrm {i}(\ell \cdot \varphi +j x)}{,}\quad \overline{u}_j(\varphi )=u_{-j}(\varphi ){,} \quad \overline{u}_{\ell j}=u_{-\ell , -j}{.} \end{aligned}$$

We define the scale of Sobolev spaces

2.7 $$\begin{aligned} H^{s}:=\Big \{ u(\varphi , x)\in L^{2}(\mathbb {T}^{\nu +1}, \mathbb {R}) : \Vert u \Vert _s^2:=\sum _{\ell \in \mathbb {Z}^{\nu }, j\in \mathbb {Z}} |u_{\ell j} |^2 \langle \ell , j \rangle ^{2 s}<\infty \Big \} \end{aligned}$$

where $\langle \ell , j \rangle :=\max \{ 1, |\ell |, |j |\}$, $|\ell |:=\sum _{i=1}^{\nu } |\ell _i |$. We shall work on the phase space $H^s\cap H_0^1(\mathbb {T}, \mathbb {R})$. We denote by $\mathfrak {B}_{r}(0, X)$ the ball of radius *r* centered at the origin of a Banach space *X*.

**Lipschitz norm** Fix $\nu \in \mathbb {N}^{*}:=\mathbb {N}{\setminus }\{0\}$ and let $\mathcal {O}$ be a compact subset of $\mathbb {R}^{\nu }$. For a function $u:\mathcal {O}\rightarrow E$, where $(E, \Vert \cdot \Vert _E)$ is a Banach space, we define the sup-norm and the lip-seminorm of *u* as

2.8 $$\begin{aligned}&\Vert u \Vert _E^{sup}:=\Vert u \Vert _{E}^{sup, \mathcal {O}} :=\sup _{\omega \in \mathcal {O}} \Vert u(\omega ) \Vert _E,\nonumber \\&\Vert u \Vert _{E}^{lip} :=\Vert u \Vert _{E}^{lip, \mathcal {O}} :=\sup _{\begin{array}{c} \omega _1, \omega _2\in \mathcal {O},\\ \omega _1\ne \omega _2 \end{array}} \frac{\Vert u(\omega _1)-u(\omega _2)\Vert _E}{|\omega _1-\omega _2|}{.} \end{aligned}$$

If *E* is finite dimensional, for any $\gamma >0$ we introduce the weighted Lipschitz norm

2.9 $$\begin{aligned} \Vert u \Vert _E^{{\gamma }, \mathcal {O}} :=\Vert u \Vert _E^{sup, \mathcal {O}}+\gamma \Vert u \Vert _{E}^{lip, \mathcal {O}}{.} \end{aligned}$$

If *E* is a scale of Banach spaces, say $E= H^s$, for $\gamma >0$ we introduce the weighted Lipschitz norm

2.10 $$\begin{aligned} \Vert u \Vert _s^{{\gamma }, \mathcal {O}}:=\Vert u \Vert _s^{sup, \mathcal {O}} +\gamma \Vert u \Vert _{s-1}^{lip, \mathcal {O}}{,} \quad \forall s\ge [\nu /2]+4 \end{aligned}$$

where we denoted by [*r*] the integer part of $r\in \mathbb {R}$.

**Linear operators** Let $A:\mathbb {T}^{\nu }\rightarrow \mathcal {L}(L^2(\mathbb {T},\mathbb {R}))$, $\varphi \mapsto A(\varphi )$, be a $\varphi$-dependent family of linear operators acting on $L^2(\mathbb {T},\mathbb {R})$. We consider *A* as an operator acting on $H^{s}(\mathbb {T}^{\nu +1},\mathbb {R})$ by setting

$$\begin{aligned} (A u)(\varphi , x)=(A(\varphi )u(\varphi , \cdot ))(x){.} \end{aligned}$$

This action is represented in Fourier coordinates as

2.11 $$\begin{aligned} A u(\varphi , x)=\sum _{j, j'\in \mathbb {Z}} A_j^{j'}(\varphi ) \,u_{j'}(\varphi )\,e^{\mathrm {i} j x}=\sum _{\ell \in \mathbb {Z}^{\nu }, j\in \mathbb {Z}} \sum _{\ell '\in \mathbb {Z}^{\nu }, j'\in \mathbb {Z}} A_{j}^{j'}(\ell -\ell ')\,u_{j' \ell '}\,e^{\mathrm {i}(\ell \cdot \varphi +j x)}{.} \end{aligned}$$

Conversely, given a Töpliz in time operator *A*, namely such that its matrix coefficients (with respect to the Fourier basis in ${\varphi },x$) satisfy

2.12 $$\begin{aligned} A_{j, \ell }^{j', \ell '}=A_j^{j'}(\ell -\ell ')\qquad \forall j, j'\in \mathbb {Z},\,\,\ell , \ell '\in \mathbb {Z}^{\nu }{,} \end{aligned}$$

we can associate it a time dependent family of operators acting on $H^s(\mathbb {T})$ by setting

$$\begin{aligned} A(\varphi ) h=\sum _{j, j'\in \mathbb {Z}, \ell \in \mathbb {Z}^{\nu }} A_j^{j'}(\ell ) h_{j'}\,e^{\mathrm {i} j x} e^{\mathrm {i}\ell \cdot \varphi }{,} \qquad \forall h\in H^s(\mathbb {T},\mathbb {R}){.} \end{aligned}$$

For $m=1, \ldots , \nu$ we define the operators $\partial _{\varphi _m} A(\varphi )$ as

2.13 $$\begin{aligned} (\partial _{\varphi _m} A(\varphi )) u(\varphi , x)= \sum _{\ell \in \mathbb {Z}^{\nu }, j\in \mathbb {Z}} \, \sum _{\ell '\in \mathbb {Z}^{\nu }, j'\in \mathbb {Z}}\mathrm {i}(\ell _m-\ell _m')\, A_j^{j'}(\ell -\ell ')\,u_{\ell ' j'}\,e^{\mathrm {i}(\ell \cdot \varphi +j x)}{.} \end{aligned}$$

We say that *A* is a *real* operator if it maps real valued functions in real valued functions. For the matrix coefficients this means that

$$\begin{aligned} \overline{A_j^{j'}(\ell )}=A_{-j}^{-j'}(-\ell ){.} \end{aligned}$$

**Hamiltonian linear operators** In the paper we shall deal with operators which are Hamiltonian according to the following Definition.

### Definition 2.1

We say that a linear map is symplectic if it preserves the 2-form $\Omega$ in (2.1); similarly we say that a linear operator *M* is Hamiltonian if *Mu* is a linear Hamiltonian vector field w.r.t. $\Omega$ in (2.1). This means that each $J^{-1} M$ is symmetric respect to the real $L^2$-scalar product. Similarly, we call a family of maps ${\varphi }\rightarrow A({\varphi })$ symplectic if, for each fixed ${\varphi }$, $A({\varphi })$ is symplectic, same for the Hamiltonians. We shall say that an operator of the form $\omega \cdot \partial _{{\varphi }}+M({\varphi })$ is Hamiltonian if $M({\varphi })$ is Hamiltonian.

*Notation* We use the notation $A\lesssim B$ to denote $A\le C B$ where *C* is a positive constant possibly depending on fixed parameters given by the problem. We use the notation $A\lesssim _y B$ to denote $A\le C(y) B$ if we wish to highlight the dependence on the variable *y* of the constant $C(y)>0$.

**Linear Tame operators** Here we introduce rigorously the spaces and the classes of operators on which we work.

### Definition 2.2

($\sigma$-*Tame operators*). Given $\sigma \ge 0$ we say that a linear operator *A* is $\sigma$-*tame* w.r.t. a non-decreasing sequence $\{\mathfrak M_A(\sigma ,s)\}_{s=s_0}^\mathcal {S}$ (with possibly $\mathcal {S}=+\infty$) if

2.14 $$\begin{aligned} \Vert A u \Vert _{s}\le \mathfrak {M}_A(\sigma ,s) \Vert u \Vert _{s_0+\sigma }+\mathfrak {M}_A(\sigma ,s_0) \Vert u \Vert _{s+\sigma } \qquad u\in H^s{,} \end{aligned}$$

for any $s_0\le s\le \mathcal {S}$. We call $\mathfrak {M}_A(\sigma ,s)$ a tame constant for the operator *A*. When the index $\sigma$ is not relevant we write $\mathfrak {M}_{A}(\sigma ,s)=\mathfrak {M}_{A}(s)$.

### Definition 2.3

(*Lip*-$\sigma$-*Tame operators*). Let $\sigma \ge 0$ and $A=A(\omega )$ be a linear operator defined for $\omega \in \mathcal {O}\subset \mathbb {R}^{\nu }$. Let us define

2.15 $$\begin{aligned} \Delta _{\omega ,\omega '}A:=\frac{A(\omega )-A(\omega ')}{|\omega -\omega '|}{,} \quad \omega ,\omega '\in \mathcal {O}{.} \end{aligned}$$

Then *A* is *Lip-*$\sigma$*-tame* w.r.t. a non-decreasing sequence $\{\mathfrak M_A(\sigma ,s)\}_{s=s_0}^\mathcal {S}$ if the following estimate holds

2.16 $$\begin{aligned} \sup _{\omega \in \mathcal {O}}\Vert Au\Vert _{s},{\gamma }\sup _{\omega \ne \omega '}\Vert (\Delta _{\omega ,\omega '}A)\Vert _{s-1} \le _{s}\mathfrak {M}^{{\gamma }}_{A}(\sigma ,s)\Vert u\Vert _{s_0+\sigma }+ \mathfrak {M}^{{\gamma }}_{A}(\sigma ,s)\Vert u\Vert _{s+\sigma }, \quad u\in H^{s}{.} \end{aligned}$$

We call $\mathfrak {M}^{\gamma }_A(\sigma ,s)$ a Lip-tame constant  of the operator *A*. When the index $\sigma$ is not relevant we write $\mathfrak {M}^{\gamma }_{A}(\sigma ,s)=\mathfrak {M}^{\gamma }_{A}(s)$.

**Modulo-tame operators and majorant norms** The modulo-tame operators are introduced in Sect. 2.2 of [15]. Note that we are interested only in the Lipschitz variation of the operators respect to the parameters of the problem, whereas in [15] the authors need to control also higher order derivatives.

### Definition 2.4

Let $u\in H^s$, $s\ge 0$, we define the majorant function $\underline{u} (\varphi , x):=\sum _{\ell \in \mathbb {Z}^{\nu }, j\in \mathbb {Z}} |u_{\ell j} |e^{\mathrm {i}(\ell \cdot \varphi +j x)}.$ Note that $\Vert u \Vert _s=\Vert \underline{u} \Vert _s$.

### Definition 2.5

(*Majorant operator*). Let $A\in \mathcal {L}(H^s)$ and recall its matrix representation (2.11). We define the majorant matrix $\underline{A}$ as the matrix with entries

$$\begin{aligned} (\underline{A})_j^{j'}(\ell ):=|(A)_{j}^{j'}(\ell ) |\qquad j, j'\in \mathbb {Z},\,\,\ell \in \mathbb {Z}^{\nu }{.} \end{aligned}$$

We consider the majorant operatorial norms

2.17 $$\begin{aligned} \Vert \underline{M}\Vert _{\mathcal L(H^s)}:= \sup _{\Vert u \Vert _s\le 1} \Vert \underline{M}u \Vert _{s}{.} \end{aligned}$$

We have a partial ordering relation in the set of the infinite dimensional matrices, i.e. if

2.18 $$\begin{aligned}&M \preceq N \Leftrightarrow |M_{j}^{j'}(\ell )|\le |N_{j}^{j'}(\ell )|\;\;\forall j,j',\ell \; \Rightarrow \Vert \underline{M} \Vert _{\mathcal L(H^s)}\le \Vert \underline{N} \Vert _{\mathcal L(H^s)}{,} \nonumber \\&\quad \Vert {M}u \Vert _s\le \Vert \underline{M}\,\underline{u} \Vert _s \le \Vert \underline{N}\, \underline{u} \Vert _s{.} \end{aligned}$$

Since we are working on a majorant norm we have the continuity of the projections on monomial subspace, in particular we define the following functor acting on the matrices

$$\begin{aligned} \Pi _K M:= {\left\{ \begin{array}{ll} M_{j}^{j'}(\ell )\qquad \text {if} \; |\ell |\le K{,} \\ 0 \qquad \qquad \text{ otherwise } \end{array}\right. } \qquad \qquad \Pi _K^\perp := \mathrm {I}-\Pi _K{.} \end{aligned}$$

Finally we define for $\mathtt {b}_0\in \mathbb {N}$

2.19 $$\begin{aligned} (\langle \partial _{\varphi }\rangle ^{\mathtt {b}_0} M )_{j}^{j'}(\ell ) := \langle \ell \rangle ^{\mathtt {b}_0} M_j^{j'}(\ell ){.} \end{aligned}$$

In the sequel let $1>{\gamma }>{\gamma }^{3/2}>0$ be fixed constants.

### Definition 2.6

(*Lip*-$\sigma$-*modulo tame*). Let $\sigma \ge 0$. A linear operator $A := A(\omega )$, $\omega \in \mathcal {O}\subset \mathbb {R}^{\nu }$, is Lip-$\sigma$-modulo-tame w.r.t. a non-decreasing sequence $\{ {\mathfrak M}_{A}^{\sharp , {\gamma }^{3/2}} (\sigma , s) \}_{s=s_0}^{\mathcal {S}}$ if the majorant operators $\underline{ A }, \underline{\Delta _{\omega ,\omega '} A}$ are Lip-$\sigma$-tame w.r.t. these constants, i.e. they satisfy the following weighted tame estimates: for $\sigma \ge 0$, for all $s \ge s_0$ and for any $u \in H^{s}$,

2.20 $$\begin{aligned} \sup _{\omega \in \mathcal {O}}\Vert \underline{A} u\Vert _s{,} \sup _{\omega \ne \omega '\in \mathcal {O}}{{\gamma }^{3/2}} \Vert \underline{\Delta _{\omega ,\omega '} A} u\Vert _s \le {\mathfrak M}_{A}^{\sharp , {\gamma }^{3/2}} (\sigma ,s_0) \Vert u \Vert _{s+\sigma } + {\mathfrak M}_{A}^{\sharp , {\gamma }^{3/2}} (\sigma ,s) \Vert u \Vert _{s_0+\sigma } {.} \end{aligned}$$

The constant ${\mathfrak M}_A^{{\sharp , {\gamma }^{3/2}}} (\sigma ,s)$ is called the modulo-tame constant of the operator *A*. When the index $\sigma$ is not relevant we write ${\mathfrak M}_{A}^{{\sharp , {\gamma }^{3/2}}} (\sigma , s) = {\mathfrak M}_{A}^{{\sharp , {\gamma }^{3/2}}} (s)$.

### Definition 2.7

We say that *A* is Lip-$-1$-modulo tame if $\langle D_x\rangle ^{1/2}{A} \langle D_x\rangle ^{1/2}$ is Lip-0-modulo tame. We denote

2.21 $$\begin{aligned} \mathfrak M^{{\sharp , {\gamma }^{3/2}}}_{A}(-1,s):= & {} \mathfrak M^{\sharp , {\gamma }^{3/2}}_{ \langle D_x \rangle ^{1/2}A \langle D_x \rangle ^{1/2}}(0,s), \nonumber \\ \mathfrak M^{\sharp , {\gamma }^{3/2}}_{A}(-1,s,a):= & {} \mathfrak M^{\sharp , {\gamma }^{3/2}}_{\langle \partial _{\varphi }\rangle ^{a} \langle D_x \rangle ^{1/2}A \langle D_x \rangle ^{1/2}}(0, s){,} \quad a\ge 0{.} \end{aligned}$$

In the following we shall systematically use $-1$ modulo-tame operators. We refer the reader to the “Appendix” of [33] for the properties of Tame and Modulo-tame operators.

**Pseudo differential operators** Following [15] we give the following definitions.

### Definition 2.8

Let $m\in \mathbb {R}$. A linear operator *A* is called pseudo differential of order $\le m$ if its action on any $H^s(\mathbb {T}, \mathbb {R})$ with $s\ge m$ is given by

$$\begin{aligned} A\sum _{j\in \mathbb {Z}} u_j e^{\mathrm {i} j x} = \sum _{j\in \mathbb {Z}} a(x,j) u_j e^{\mathrm {i}j x} {,} \end{aligned}$$

where *a*(*x*, *j*), called the *symbol* of *A*, is the restriction to $\mathbb {T}\times \mathbb {Z}$ of a complex valued function *a*(*x*, *y*) which is $C^{\infty }$ smooth on $\mathbb {T}\times \mathbb {R}$, $2\pi$-periodic in *x* and satisfies

2.22 $$\begin{aligned} |\partial _{x}^{\alpha }\partial _{y}^{\beta }a(x,y)|\le C_{\alpha ,\beta }\langle y\rangle ^{m-\beta }{,} \;\;\forall \; \alpha ,\beta \in \mathbb {N}{.} \end{aligned}$$

We denote by $A[\cdot ]=\mathrm{Op}(a)[\cdot ]$ the pseudo operator with symbol $a:=a(x, j)$. We call $OPS^m$ the class of the pseudo differential operator of order less or equal to *m* and $OPS^{-\infty }:=\bigcap _m OPS^m$. We define the class $S^m$ as the set of symbols which satisfies (2.22).

We will consider mainly operators acting on $H^s(\mathbb {T},\mathbb {R})$ with a quasi-periodic time dependence. In the case of pseudo differential operators this corresponds^1^ to considering symbols $a(\varphi , x, y)$ with $\varphi \in \mathbb {T}^{\nu }$. Clearly these operators can be thought as acting on functions $u({\varphi },x)=\sum _{j\in \mathbb {Z}}u_{j}({\varphi })e^{\mathrm{i}jx}$ in $H^s(\mathbb {T}^{\nu +1},\mathbb {R})$ in the following sense:

$$\begin{aligned} (Au)({\varphi },x)=\sum _{j\in \mathbb {Z}}a({\varphi },x,j) u_{j}({\varphi })e^{\mathrm {i} jx}{,} \quad a({\varphi },x,j)\in S^{m}{.} \end{aligned}$$

The symbol $a(\varphi , x, y)$ is $C^{\infty }$ smooth also in the variable $\varphi$. We still denote $A:=A(\varphi )=\mathrm{Op}(a(\varphi , \cdot ))=\mathrm{Op}(a)$.

### Definition 2.9

Let $a:=a({\varphi },x, y)\in S^{m}$ and set $A:=\mathrm{Op}(a)\in OPS^{m}$,

2.23 $$\begin{aligned} |A|_{m,s,\alpha }:=\max _{0\le \beta \le \alpha } \sup _{y\in \mathbb {R}}\Vert \partial _{y}^{\beta }a(\cdot ,\cdot , y)\Vert _{s} \langle y\rangle ^{-m+\beta }{.} \end{aligned}$$

We will use also the notation $|a |_{m, s, \alpha }:=|A|_{m,s,\alpha }$.

Note that the norm $|\cdot |_{m,s,\alpha }$ is non-decreasing in *s* and $\alpha$. Moreover given a symbol $a({\varphi },x)$ independent of *y*, the norm of the associated multiplication operator $\mathrm{Op}(a)$ is just the $H^{s}$ norm of the function *a*. If on the contrary the symbol *a*(*y*) depends only on *y*, then the norm of the corresponding Fourier multipliers $\mathrm{Op}(a(y))$ is just controlled by a constant.

As in formula (2.10), if $A=\mathrm{Op}(a(\omega ,{\varphi },x, y))\in OPS^{m}$ is a family of pseudo differential operators with symbols $a(\omega ,{\varphi },x, y)$ belonging to $S^{m}$ and depending in a Lipschitz way on some parameter $\omega \in \mathcal {O}\subset \mathbb {R}^{\nu }$, we set

2.24 $$\begin{aligned} |A|_{m,s,\alpha }^{{\gamma },\mathcal {O}}:=\sup _{\omega \in \mathcal {O}}|A|_{m,s,\alpha }+ {\gamma }\sup _{\omega _1,\omega _2\in \mathcal {O}}\frac{{|\mathrm Op}\big (a(\omega _1,{\varphi },x, y)-a(\omega _2,{\varphi },x, y)\big )|_{m,s-1,\alpha }}{|\omega _1-\omega _2|}{.} \end{aligned}$$

For the properties of compositions, adjointness and quantitative estimates of the actions on the Sobolev spaces $H^{s}$ of pseudo differential operators we refer to “Appendix B” of [33].

## Weak Birkhoff Normal Form

The aim of this section is to construct a $\xi$-parameter family of approximately invariant, finite dimensional tori supporting quasi-periodic motions with frequency $\omega (\xi )$. We will impose the map $\xi \mapsto \omega (\xi )$ to be a diffeomorphism and we will consider such approximate solutions as the starting point for the Nash–Moser algorithm.

In order to state the main result of this section, we need some preliminary definitions.

We write the DP Hamiltonian in (1.4) in the following way:

3.1 $$\begin{aligned} H(u)&=H^{(2)}(u)+H^{(3)}(u)+H^{(\ge 9)}\, , \nonumber \\ H^{(2)}(u)&:=\frac{1}{2} \int _{\mathbb {T}} u^2\,dx{,} \quad H^{(3)}(u):=-\frac{1}{6}\int _{\mathbb {T}} u^3\,dx{,} \quad H^{(\ge 9)}(u):=\int _{\mathbb {T}} f(u)\,dx\, . \end{aligned}$$

Recall *S* in (1.9) and define $S^{c}:=\mathbb {Z}{\setminus }\big (S\cup \{0\}\big )$. We decompose the phase space as

3.2 $$\begin{aligned} H_0^1(\mathbb {T}):=H_S\oplus H_S^{\perp }{,} \quad H_S:=\text{ span }\{ e^{\mathrm {i}\,j\,x} : j\in S \}{,} \quad H_S^{\perp }:=\text{ span }\{ e^{\mathrm {i}\,j\,x} : j\in S^{c} \}{,} \end{aligned}$$

and we denote by $\Pi _S, \Pi _S^{\perp }$ the corresponding orthogonal projectors. The subspaces $H_S$ and $H_S^{\perp }$ are symplectic orthogonal respect to the 2-form $\Omega$ (see (2.1)). We write

$$\begin{aligned} u=v+z{,} \quad v:=\Pi _S u:=\sum _{j\in S} u_j\,e^{\mathrm {i}\,j\,x}{,} \quad z=\Pi _S^{\perp } u:=\sum _{j\in S^c} u_j\,e^{\mathrm {i}\,j\,x}{.} \end{aligned}$$

For a finite dimensional space

3.3 $$\begin{aligned} E:=E_C:=\text{ span }\left\{ e^{\mathrm {i}\,j\,x} : 0<|j |\le C \right\} {,} \quad C>0{,} \end{aligned}$$

let $\Pi _E$ denote the corresponding $L^2$-projector on *E*. The notation $R(v^{k-q} z^q)$ indicates a homogeneous polynomial of degree *k* in (*v*, *z*) of the form

$$\begin{aligned} R(v^{k-q} z^q)=M[\underbrace{v, \ldots , v}_{(k-q)\mathrm{-times}},\underbrace{z, \ldots , z}_{q\mathrm{-times}}\,]{,} \quad M=k\text{-linear }{.} \end{aligned}$$

We denote with $H^{(n, \ge k)}, H^{(n, k)}, H^{(n, \le k)}$ the terms of type $R(v^{n-s}\, z^s)$, where, respectively, $s\ge k, s=k, s\le k$, that appear in the homogeneous polynomial $H_n$ of degree *n* in the variables (*v*, *z*). Given an *n*-uple $\{j_1,\ldots , j_n\}\subset \mathbb Z{\setminus }\{0\}$ and a set $B\subset \mathbb Z{\setminus }\{0\}$ we define

$$\begin{aligned} \sharp (\{j_1,\ldots , j_n\},B) :=\text{ number } \text{ of } j_i \text{ belonging } \text{ to } B{.} \end{aligned}$$

Now we start the “weak” Birkhoff normal form procedure, i.e. we look for a change of coordinates which normalizes the terms in (3.1) independent and linear in the normal variable *z*.

As it is well known, one of the main problem of the Birkhoff normal form procedures is to deal with the resonances given by the equations (1.15) = 0 which arise from considering the kernel of the adjoint action $\mathrm {ad}_{H^{(2)}}$ (see (2.4)). It turns out that when $n\ge 2$ there are many non-trivial solutions of (1.15) = 0. A way to deal with this problem is to exploit the integrability of the DP equation.

In [32] the authors construct an infinite number of conserved quantities $K_n$ for the Eq. (1.1) with $f=0$ starting from the ones given in [28]. By an explicit characterization of the quadratic part of each $K_n$, they deduce that, at a purely formal level, the Birkhoff normal form of the Degasperis–Procesi equation is action preserving (or integrable). Here we rename these constants of motion in the following way, writing only the quadratic parts (which are fundamental for the study of the Birkhoff resonances at $u=0$)

3.4 $$\begin{aligned} K_0(u)&:=H(u){,} \qquad K_{1}(u):=\frac{1}{2}\int _{\mathbb {T}} (J^{-1} u_x) \,u\,dx{,}\nonumber \\ K_{n+2}&:=\int _{\mathbb {T}} (\partial _x^n w)^2\,dx+O(u^3){,} \quad n\ge 0{,} \end{aligned}$$

where we denoted by

3.5 $$\begin{aligned} w:=\Lambda ^{-1} u:=u-u_{xx}{,} \qquad \Lambda :=(1-\partial _{xx})^{-1}{.} \end{aligned}$$

We remark that $K_1$ is the *momentum* Hamiltonian arising from the translation invariance of the equation.

### Definition 3.1

Given a quadratic diagonal Hamiltonian $Q(u)=\sum _j \mathfrak {l}(j) |u_j |^2$, we define $\Pi _{\mathrm{Ker}(Q)}$ as the projection on the kernel of the adjoint action (recall (2.2) and $J=\mathrm {diag}_{j\in \mathbb {Z}}(\lambda (j))$)

3.6 $$\begin{aligned} \mathrm {ad}_{Q}(K)= & {} \sum _{j_1, \ldots , j_n} \Big ( \sum _{i=1}^n \mathfrak {l}(j_i) {\lambda }(j_i)\Big ) K_{j_1 \ldots j_n} u_{j_1}\ldots u_{j_n}{,}\nonumber \\ \quad K(u):= & {} \sum _{j_1, \ldots , j_n} K_{j_1 \ldots j_n} u_{j_1}\ldots u_{j_n}. \end{aligned}$$

We define the projector on the range of the adjoint action as $\Pi _{\mathrm{Rg}(Q)}:=\mathrm {I}-\Pi _{{\text{ Ker }(Q)}}$.

We say that *K*, as in (3.6), “preserves” momentum if and only if

$$\begin{aligned} \Big ( \sum _{i=1}^n j_i \Big ) K_{j_1 \ldots j_n}=0 \qquad \forall j_1, \ldots , j_n\in \mathbb {Z}{\setminus }\{0\}{.} \end{aligned}$$

The main result of this section is the following.

### Proposition 3.2

There exist $r>0$, depending on *S* (see (1.9)), and an analytic symplectic change of coordinates

3.7 $$\begin{aligned} \Phi _B:\mathcal B_{r}(0,H_0^1(\mathbb {T}))\rightarrow H_0^1(\mathbb {T}){,} \qquad \Phi _B= \mathrm {I} + \Psi {,}\quad \Psi = \Pi _E \circ \Psi \circ \Pi _E \end{aligned}$$

where *E* is a finite dimensional space as in (3.3), such that the Hamiltonian *H* in (3.1) transforms into

3.8 $$\begin{aligned} \mathcal {H}:= H\circ \Phi _B = H^{(2)} +\mathcal {H}^{(4,0)} +\mathcal {H}^{(6,0)} +\mathcal {H}^{(8, 0)}+\mathcal {H}^{(\ge 9, \le 1)} + \mathcal H^{(\ge 3,\ge 2)}{,} \end{aligned}$$

where

3.9 $$\begin{aligned} \mathcal {H}^{(3, \ge 2)}&:=-\frac{1}{2}\int _{\mathbb {T}} v\, z^2\,dx-\frac{1}{6} \int _{\mathbb {T}} z^3\,dx{,} \nonumber \\ \mathcal {H}^{(4,0)}&\! :=\! \frac{1}{2}\sum _{j\in S^+} \,\frac{{\lambda }(2 j)}{2{\lambda }(j)-{\lambda }(2 j)}\,|u_j |^4 \!+\!\sum _{\begin{array}{c} j_1, j_2\in S^+,\\ j_1- j_2\ne 0 \end{array}} \frac{{\lambda }(j_1\!+\!j_2)}{{\lambda }(j_1)\!+\!{\lambda }(j_2)-{\lambda }(j_1\!+\!j_2)} |u_{j_1} |^2|u_{j_2}|^2\nonumber \\&\quad +\sum _{\begin{array}{c} j_1, j_2\in S^+,\\ j_1 - j_2\ne 0 \end{array}} \frac{{\lambda }(j_1-j_2)}{{\lambda }(j_1)-{\lambda }(j_2)-{\lambda }(j_1-j_2)} |u_{j_1} |^2|u_{j_2}|^2 \end{aligned}$$

and $\mathcal {H}^{(k, 0)}=\Pi _{\mathrm{Ker}(H^{(2)})} \mathcal {H}^{(k, 0)}$ with $k=4, 6, 8$ depend only on $|u_j |^2$. The same change of variables $\Phi _B$ puts all the Hamiltonians in (3.4) in weak Birkhoff normal form up to order eight as in (3.8). In particular we have $K_1\circ \Phi _B=K_1$.

In order to prove the Proposition 3.2 above we need some preliminary results proved in detail in [32].

### Definition 3.3

(*M*-*resonances*). Fix $M\in \mathbb {N}$, $M\ge 3$. We recall that the quadratic part of *H* and $K_r$, $2\le r\le M$, in (3.4) are

$$\begin{aligned} K^{(2)}_r(u):=\sum _{j\in \mathbb {Z}{\setminus }\{0\}} (1+j^2)^2\,j^{2 (r-2)}\,|u_{j} |^2{,} \quad H^{(2)}(u)=\sum _{j\ge 1} |u_{j} |^2{.} \end{aligned}$$

We say that an *n*-uple $\{j_1,\ldots ,j_{n}\}\subset \mathbb {Z}{\setminus }\{0\}$, with $n\le M$, is a *M*-*resonance* of order *n* for the DP hierarchy if

3.10 $$\begin{aligned} \sum _{i=1}^n j_i=0{,} \quad \sum _{i=1}^n {\lambda }(j_i)=0{,} \quad \sum _{i=1}^n (1+j_i^2)^2\,j_i^{2 (r-2)}{\lambda }(j_i)=0\, \quad \forall r=2, \ldots , M+1{.} \end{aligned}$$

### Proposition 3.4

Fix $M\in \mathbb {N}$, $M\ge 3$. All the *M*-resonances of the DP equations (3.4) are trivial, namely there are no resonances of odd order and the even ones are, up to permutations, of the form

3.11 $$\begin{aligned} (i, -i, j, -j,k, -k, p, -p, \ldots ){.} \end{aligned}$$

### Proof

Since this Proposition is proved in [32] with different notations, for completeness we restate here a concise proof by induction on *M*. For $M=3$ the thesis follows trivially: indeed direct computations show that

$$\begin{aligned} \sum _{i=1}^3 j_i=0, \quad \sum _{i=1}^3 {\lambda }(j_i)=0 \; \Leftrightarrow \; j_1=-j_2{,}\; j_3=0 \end{aligned}$$

up to permutations, and this solution is incompatible with $j_i\in \mathbb {Z}{\setminus }\{0\}$.

Let us now suppose that the thesis is true up to $M-1\ge 3$ and prove it for *M*. We start by noticing that if $n<M$ then (3.10) with $r\le M-1$ can hold only if $\{j_1,\ldots ,j_{n}\}$ is a $M-1$ resonance of order *n*. The inductive hypothesis then says that $\{j_1,\ldots ,j_{n}\}$ is trivial. Similarly if $\{j_1,\ldots ,j_{n}\}$ contains a trivial resonance, i.e. if $j_{i_1}+ j_{i_2}=0$ for $1\le i_1,i_2\le n$, then $j_{i_1}, j_{i_2}$ do not appear in (3.10) and hence $\{j_1,\ldots ,j_{n}\}$ is an *M*-resonance of order *n* if and only if

$$\begin{aligned} \{j_1,\ldots ,j_{n}\}{\setminus } \{j_{i_1}, j_{i_2}\}{,} \quad \text{ is } \text{ an } M-2 \text{ resonance } \text{ of } \text{ order } n-2. \end{aligned}$$

Without loss of generality we assume that $n=M$ and that $j_{i_1}+ j_{i_2}\ne 0$ for any $1\le i_1,i_2\le M$.

Up to a permutation we can assume that for some $M\ge k\ge 1$ and $\alpha _1,\ldots ,\alpha _k\ge 1$ one has

$$\begin{aligned} \{j_1,\ldots ,j_{n}\}= \{\underbrace{\widehat{\jmath }_1,\ldots , \widehat{\jmath }_1}_{\alpha _1}, \ldots , \underbrace{\widehat{\jmath }_k,\ldots , \widehat{\jmath }_k}_{\alpha _k}\}{.} \end{aligned}$$

Consequently rewrite the third equation in (3.10) as $\sum _{i=1}^{k} \alpha _i (1+\widehat{\jmath }_i{}^2)^2\, \widehat{\jmath }_i{}^{2 (r-2)}{\lambda }(\widehat{\jmath }_i)=0$, $\forall r=2, \ldots , M+1$. Then we can extract *k* equations from these ones and write them in the form

3.12 $$\begin{aligned} \begin{pmatrix} 1 &{} \ldots &{} 1\\ \widehat{\jmath }_1{}^{2} &{} \ldots &{} \widehat{\jmath }_k{}^{2} \\ \vdots &{} &{} \vdots \\ \widehat{\jmath }_1{}^{2(k-1)} &{} \ldots &{} \widehat{\jmath }_k{}^{2(k-1)} \end{pmatrix} \begin{pmatrix} \alpha _1(1+\widehat{\jmath }_1{}^2)^2\,{\lambda }(\widehat{\jmath }_1) \\ \vdots \\ \alpha _k(1+\widehat{\jmath }_k{}^2)^2\,{\lambda }(\widehat{\jmath }_k) \end{pmatrix} = \begin{pmatrix} 0 \\ \vdots \\ 0 \end{pmatrix}{.} \end{aligned}$$

The determinant of the Vandermonde matrix in (3.12) is $\prod _{i\ne h} (\widehat{\jmath }_i{}^2-\widehat{\jmath }_h{}^2)\ne 0,$ since, by hypothesis, $\widehat{\jmath }_i\ne \pm \widehat{\jmath }_h$. Then the only possible solution corresponds to $\widehat{\jmath }_i=0$ for all *i*, which is not compatible with $\widehat{\jmath }_i\in \mathbb {Z}{\setminus }\{0\}$. $\quad \square$

### Remark 3.5

Notice that if $j_1, \ldots , j_N\in \mathbb {Z}{\setminus }\{ 0\}$, $j_1+\cdots + j_N=0$ and $\#(\{ j_1, \ldots , j_N \}, S^c)\le 1$, then $\max _{i=1,\ldots , N} |j_i |\le (N-1) \overline{\jmath }_1$. Thus, the vector field $X_{F^{(N,\le 1)}}$, generated by the finitely supported Hamiltonian

3.13 $$\begin{aligned} F^{(N,\le 1)}=\sum _{\begin{array}{c} j_1+\cdots +j_N=0 \\ \#(\{ j_1, \ldots , j_N \}, S^c)\le 1 \end{array}} F^{(N,\le 1)}_{j_1 \ldots j_N} u_{j_1}\ldots u_{j_N}\, \end{aligned}$$

is finite rank, and, in particular, it vanishes outside the finite dimensional subspace $E:=E_{(N-1) \overline{\jmath }_1}$ (see (3.3) ) and it has the form

$$\begin{aligned} X_{F^{(N,\le 1)}}(u)=\Pi _E X_{F^{(N,\le 1)}} (\Pi _E u){.} \end{aligned}$$

Therefore its flow $\Phi ^{(N)}$ is analytic and invertible on the phase space $H_0^1(\mathbb {T})$, provided that $| \Pi _E u|$ is appropriately small.

In order to prove Proposition 3.2 we need the following result.

### Proposition 3.6

Fix $M\in \mathbb {N}$, $M\ge 2$ and consider *H* in (1.4) and $K_m$, $m=1, \ldots , M$, in (3.4). Then, for any $N\le M-2$, there exists $r>0$ and an analytic symplectic change of coordinates $\Phi _N^{\pm 1} :\mathcal B_r(0,H_0^1(\mathbb {T}))\rightarrow H_0^1(\mathbb {T})$ of the form

3.14 $$\begin{aligned} \Phi _N= \mathrm {I} + \Psi _N{,} \quad \Phi _0=\mathrm {I},\quad \Psi _N(u)= \Pi _E \circ \Psi _N \circ \Pi _E , \end{aligned}$$

where *E* is a finite dimensional space as in (3.3), such that

3.15 $$\begin{aligned}&H\circ \Phi _N^{-1}=H^{(2)}+Z_N^{(\le N+2, 0)}+R_N^{(\ge N+3, \le 1)} +H_{N}^{(\ge 3, \ge 2)}{,}\qquad \;\; K_1\circ \Phi _N^{-1}=K_1{,}\nonumber \\&K_m\circ \Phi _N^{-1}=K_m^{(2)}+W_{m, N}^{(\le N+2, 0)} +Q_{m, N}^{(\ge N+3, \le 1)} +K_{m,N}^{(\ge 3, \ge 2)}{,} \quad m=2, \ldots , M{,} \end{aligned}$$

where $$Z_N^{(\le N+2, 0)}, W^{(\le N+2, 0)}_{m, N}\in \bigcap _{m=1}^M \text{ Ker }(K_m^{(2)}) \cap \text{ Ker }(H^{(2)})$$.

### Proof

The terms of degree at most 2 in the variable *z* are not affected by the procedure that we are going to describe.

We argue the result by induction on the number of steps *N*. For $N=0$ it is trivial since $\Phi _0$ is the identity map.

Suppose that we have performed *N* steps. By the fact that $\{ H, K_m \}=0$ then $\{ H, K_m\}\circ \Phi _N^{-1}=0$. For the latter, we are interested in the corresponding equations for the terms of homogeneity at most $N+3$ and degree in the variable *z* less or equal than one. So we consider the projection $\Pi ^{(\le N+3, \le 1)}\Big (\{ H, K_m\}\circ \Phi _N^{-1}\Big )=0$ and we get, for any $m=1, \ldots , M$, the following system of equations $\{ H^{(2)}, K_m^{(2)}\}=0\,$ and

$$\begin{aligned}&\{ H^{(2)}, W_{N, m}^{(N+2, 0)} \} +\{ Z^{(N+2, 0)}_N, K_m^{(2)} \} +\Pi ^{(\le N+2)}\{ Z_N^{(N+2, 0)}, W_{N, m}^{(N+2, 0)}\}=0{,}\\&\Pi ^{(N+3)}\{ Z_N^{(N+2, 0)}, W_{N, m}^{(N+2, 0)}\} +\{ H^{(2)}, Q_{m,N}^{(N+3, \le 1)}\} +\{ R_N^{(N+3, \le 1)}, K_m^{(2)} \}=0{.} \end{aligned}$$

By the inductive hypothesis $W_{N, m}^{(N+2, 0)}, Z_N^{(N+2, 0)}\in \bigcap _{m=1}^M \text{ Ker }(K_m^{(2)}) \cap \text{ Ker }(H^{(2)})$, hence

$$\begin{aligned} \{ H^{(2)}, W_{N, m}^{(N+2, 0)}\}=\{ Z_N^{(N+2, 0)}, K_m^{(2)}\}=0 \end{aligned}$$

and

3.16 $$\begin{aligned} \{ H^{(2)}, Q_{m,N}^{(N+3, \le 1)}\}+\{ R_N^{(N+3, \le 1)}, K_m^{(2)} \}=0{,} \quad m=1, \ldots , M{,} \end{aligned}$$

since $$\{ H^{(2)}, Q_{m,N}^{(N+3, \le 1)}\}\in \text{ Rg }(H^{(2)})$$ and $$\{ R_N^{(N+3, \le 1)}, K_m^{(2)} \}\in \text{ Rg }(K_m^{(2)})$$.

We note the following fact, which derives from the Jacobi identity: if $f\in \text{ Ker }(H^{(2)})$ then $\{ f, K_m^{(2)}\}\in \text{ Ker }(H^{(2)})$.

Then we have that $\{ \Pi _{\mathrm{Ker}(H^{(2)})} R_N^{(N+3, \le 1)}, K_m^{(2)} \} \in \text{ Ker }(H^{(2)})$ and by (3.16)

$$\begin{aligned}&\{ \Pi _{\mathrm{Ker}(H^{(2)})} R_N^{(N+3, \le 1)}, K_m^{(2)} \}= -\{ \Pi _{\mathrm{Rg}(H^{(2)})} R_N^{(N+3, \le 1)}, K_m^{(2)} \}\\&\quad +\{ H^{(2)}, Q_{N, m}^{(N+3, \le 1)}\}\in \text{ Rg }(H^{(2)}){.} \end{aligned}$$

Thus $$\{ \Pi _{\mathrm{Ker}(H^{(2)})} R_N^{(N+3, \le 1)}, K_m^{(2)} \}=0$$ and

$$\begin{aligned} \Pi _{\mathrm{Ker}(H^{(2)})} R_N^{(N+3, \le 1)} =\Pi _{\mathrm{Ker}(H^{(2)})} \Pi _{\mathrm{Ker}(K_m^{(2)})} R_N^{(N+3, \le 1)}{.} \end{aligned}$$

By symmetry $\Pi _{\mathrm{Ker}(K_m^{(2)})} Q_{m, N}^{(N+3, \le 1)} =\Pi _{\mathrm{Ker}(H^{(2)})} \Pi _{\mathrm{Ker}(K_m^{(2)})} Q_{m, N}^{(N+3, \le 1)}$. Hence

3.17 $$\begin{aligned} \Pi _{\mathrm{Rg}(H^{(2)})} \Pi _{\mathrm{Ker}(K_m^{(2)})} Q_{m, N}^{(N+3, \le 1)} =\Pi _{\mathrm{Rg}(K_m^{(2)})} \Pi _{\mathrm{Ker}(H^{(2)})} R_N^{(N+3, \le 1)}=0{,} \quad m=1,\ldots , M{.} \end{aligned}$$

In order to obtain the Birkhoff normal form at order $N+3$ we consider a Birkhoff transformation $\Phi _{F^{(N+3,\le 1)}}$ with generator $F^{(N+3),\le 1}$ of the form (3.13) ( with $N\rightsquigarrow N+3$) and we define $\Phi _{N+1}:= \Phi _{F^{(N+3,\le 1)}} \circ \Phi _N$. By Remark 3.5 the flow $\Phi _{F^{(N+3,\le 1)}}$ is well defined in an appropriately small ball and it has the form Identity plus a finite rank operator. Note that, since $F^{(N+3,\le 1)}$ is Fourier supported on $(j_1, \ldots , j_{N+3})$ such that $j_1+\cdots +j_{N+3}=0$, the Hamiltonian $K_1$ commutes with $F^{(N+3,\le 1)}$ and, by the inductive hypothesis, $K_1\circ \Phi _{N+1}^{-1}=K_1$. The function $F^{(N+3,\le 1)}$ is chosen in order to solve the homological equation

$$\begin{aligned} \{ H^{(2)}, F^{(N+3,\le 1)}\}=\Pi _{\mathrm{Rg}(H^{(2)})} R_N^{(N+3, \le 1)} {\mathop {=}\limits ^{(3.17)}} \Pi _{\mathrm{Rg}(K_m^{(2)})}\Pi _{\mathrm{Rg}(H^{(2)})} R_N^{(N+3, \le 1)}{.} \end{aligned}$$

We now show that $F^{(N+3,\le 1)}$ solves also the homological equation for the commuting Hamiltonians $K_m\circ \Phi _N^{-1}$, $m=1, \ldots , M$. Indeed, by the fact that $\mathrm {ad}_{H^{(2)}}^{-1}$ commutes with $\mathrm {ad}_{K_m^{(2)}}$ on the intersection $\text{ Rg }(H^{(2)})\cap \text{ Rg }(K_m^{(2)})$, we have

$$\begin{aligned} \{ K_m^{(2)}, F^{(N+3,\le 1)}\}= \mathrm {ad}_{H^{(2)}}^{-1}\{ K_m^{(2)}, \Pi _{Rg(K_m^{(2)})}\Pi _{Rg(H^{(2)})} R_N^{(N+3, \le 1)} \}{,} \end{aligned}$$

and by (3.16), (3.17) we get

$$\begin{aligned} \{ K_m^{(2)}, \Pi _{\mathrm{Rg}(K_m^{(2)})} \Pi _{\mathrm{Rg}(H^{(2)})} R_N^{(N+3, \le 1)} \} =\{H^{(2)}, \Pi _{\mathrm{Rg}(K_m^{(2)})}\Pi _{\mathrm{Rg}(H^{(2)})} Q_{m, N}^{(N+3, \le 1)} \}{.} \end{aligned}$$

By (3.17) we have that the resonant term $Z^{(N+3, \le 1)}_{N+1}:=\Pi _{\mathrm{Ker}(H^{(2)})} R_N^{(N+3, \le 1)}$ belongs to the intersection of the kernels and by Proposition 3.4 these terms are supported only on *n*-ples of indices of the form $(i, -i, j, -j, k, -k, \ldots )$. By the symmetry of the tangential set *S* this is possible for a set of indices with at most one outside *S* if and only if all the indices belong to *S*. Hence $Z^{(N+3, 1)}_{N+1}=0$ and we define $Z_{N+1}^{(\le N+3, 0)}:=Z_{N+1}^{(N+3, 0)}+Z_N^{(\le N+2, 0)}$. We do not compute explicitly the radius *r* of the ball in which we can perform the Birkhoff change of variables, however one can easily check that $r\rightarrow 0$ as $N\rightarrow \infty$ or as $\mathtt {r}\rightarrow 0$ in Definition 1.9. $\quad \square$

### Proof of Proposition 3.2

We apply Proposition 3.6 with $N=6$ and $M=8$ and we obtain (3.7), (3.8) by setting $\Phi _B:=\Phi _N^{-1}$. To prove (3.9) we have to show explicitly the computations of the first step of Birkhoff normal form.

First we remove the cubic terms independent of *z* and linear in *z* from the Hamiltonian

3.18 $$\begin{aligned} H^{(3)}= -\frac{1}{6} \int _{\mathbb {T}} u^3\,dx= -\frac{1}{6} \int _{\mathbb {T}}v^3\,dx-\frac{1}{2}\int _{\mathbb {T}} v^2 z\,dx -\frac{1}{2}\int _{\mathbb {T}} v z^2\,dx-\frac{1}{6}\int _{\mathbb {T}} z^3\,dx{.} \end{aligned}$$

We consider $\Phi _1:=(\Phi ^t_{F^{(3,\le 1)}})_{|_{t=1}}$ as the time-1 flow map generated by the Hamiltonian vector field $X_{F^{(3,\le 1)}}$, with an auxiliary Hamiltonian $F^{(3,\le 1)}$ of the form (3.13) with $N=3$. The transformed Hamiltonian is $H_1:=H\circ \Phi _1^{-1}=H^{(2)}+H_1^{(3)}+H_1^{(4)}+H_1^{(\ge 5)}$ with

3.19 $$\begin{aligned} H_1^{(3)}\!:=\!\{ F^{(3,\le 1)}, H^{(2)}\}\!+\!H^{(3)}{,} \quad H_1^{(4)}\!:=\! \frac{1}{2}\{ F^{(3,\le 1)} , \{F^{(3,\le 1)}, H^{(2)}\}\}\!+\!\{ F^{(3,\le 1)}, H^{(3)}\}{,} \end{aligned}$$

and where $H_1^{(\ge 5)}$ collects all the terms of order at least five in (*v*, *z*). We choose $F^{(3,\le 1)}$ such that the following homological equation holds

3.20 $$\begin{aligned} \{ F^{(3,\le 1)}, H^{(2)}\}+H^{(3)}=H^{(3, \ge 2)} \quad \Leftrightarrow \quad \{H^{(2)}, F^{(3,\le 1)}\}=\Pi _{\mathrm{Rg}(H^{(2)})} H^{(3, \le 1)}{.} \end{aligned}$$

Recalling (2.2) and (3.18), the solution of the Eq. (3.20) is given by $F^{(3,\le 1)}$ as in (3.13) with $N=3$ with coefficients defined as

3.21 $$\begin{aligned} F_{j_1 j_2 j_3}^{(3,\le 1)}:={\left\{ \begin{array}{ll} \dfrac{1}{6\,\mathrm {i}\, ({\lambda }(j_1)+{\lambda }(j_2)+{\lambda }(j_3))} \qquad \text{ if }\,\,\sharp (\{ j_1, j_2, j_3 \}, S^c)\le 1,\, j_1+j_2+j_3=0{,}\\ 0 \qquad \qquad \qquad \qquad \qquad \qquad \qquad \qquad \text{ otherwise }{.} \end{array}\right. } \end{aligned}$$

The Hamiltonian $F^{(3,\le 1)}$ is well defined since, by Proposition 3.4, there are no non-trivial 3-resonances of order 3. Since $\Pi _{\mathrm{Rg}(H^{(2)})} H^{(3, \le 1)}=H^{(3, \le 1)}$ we get (see (3.19), (3.20))

3.22 $$\begin{aligned} H^{(3)}_1= H^{(3, \ge 2)}{,} \qquad H_1^{(4)}=\frac{1}{2}\{F^{(3,\le 1)}, H^{(3, \le 1)}\}+\{ F^{(3,\le 1)}, H^{(3, \ge 2)}\}{.} \end{aligned}$$

In the second step we normalize the terms of total degree 4 and $\le 1$ in the variable *z*. The term $\Pi _{\mathrm{Ker}(H^{(2)})}H_1^{(4,\le 1)}$ is Fourier supported on the set of 4-resonances of order 4, which are trivial by Proposition 3.4. By Proposition 3.6$\Pi _{\mathrm{Ker}(H^{(2)})} H_1^{(4, 1)}=0$. Thus we have to compute only $\Pi _{\mathrm{Ker}(H^{(2)})} H_1^{(4, 0)}$. We have

3.23 $$\begin{aligned} Z_2^{(4,0)}\!=\! \Pi _{\mathrm{Ker}(H^{(2)})}H^{(4, 0)}_1\!=\!\frac{1}{8} \sum _{\begin{array}{c} j_1, j_2, j_3, j_4\in S,\\ j_1+j_2+j_3+j_4=0\\ j_1\!+\!j_2\ne 0,\, j_3+j_4\ne 0,\\ \sum _{k=1}^4 {\lambda }(j_k)=0 \end{array} }\frac{{\lambda }(j_1+j_2)}{{\lambda }(j_1)+{\lambda }(j_2)-{\lambda }(j_1+j_2)}\,u_{j_1} u_{j_2} u_{j_3} u_{j_4}. \end{aligned}$$

The remaining steps of this procedure do not affect the terms with degree of homogeneity less or equal than 4. Hence by (3.23), the fact that ${\lambda }(-j)=-{\lambda }(j)$ (see (1.8)) and the symmetry of *S* we obtain (3.9). $\quad \square$

## Action-Angle Variables

On the submanifold $\{ z=0\}$ we put the following action-angle variables

4.1 $$\begin{aligned} \mathbb {T}^{\nu }\times [0, \infty )^{\nu } \longrightarrow \{ z=0\}{,} \qquad (\theta , I) \longmapsto v=\sum _{j\in S} \sqrt{I_j} \,e^{\mathrm {i} \theta _j}\,e^{\mathrm {i} j x} . \end{aligned}$$

Note that this change of coordinates is real if and only if $I_{-j}=I_j$ and $\theta _{-j}=-\theta _j$. The symplectic form in (2.1) restricted to the subspace $H_S$ transforms into the 2-form $\sum _{j\in S^+}\frac{1}{{\lambda }(j)} d\theta _j\wedge \, d I_j{.}$ We have that the Hamiltonian $\mathcal {H}^{(\le 8)}(\theta , I, 0)=\sum _{j\in S^+} I_j +\mathcal {H}^{(4, 0)}(I)+\mathcal {H}^{(6, 0)}(I)+\mathcal {H}^{(8, 0)}(I)$ depends only by the actions *I* and its equations of motion read as

4.2 $$\begin{aligned} {\left\{ \begin{array}{ll} \dot{\theta }_j={\lambda }(j)\,\,\partial _{I_j} \mathcal {H}^{(\le 8)}(\theta , I, 0){,} \qquad \quad \quad j\in S^+{,}\\ \dot{I}_j=-\lambda (j)\partial _{\theta _j} \mathcal {H}^{(\le 8)}(\theta , I, 0)=0{,} \qquad \quad \,\,\,\, j\in S^+{,} \end{array}\right. } \end{aligned}$$

where, by (3.9),

4.3 $$\begin{aligned} \partial _{I_j} \mathcal {H}^{(\le 8)}(\theta , I, 0)&=1+\frac{ {\lambda }(2 j)}{4 {\lambda }(j)-2{\lambda }(2 j)}\, I_j+ \sum _{k\in S^+, k\ne j}\mathtt {b}_{j k}\,I_k+O(I^2)\, , \quad j\in S^+\, , \end{aligned}$$

4.4 $$\begin{aligned} \qquad \mathtt {b}_{j k}:&=\frac{2}{3} \frac{(1+k^2)(1+j^2)(2+k^2+j^2)}{(3+k^2+j^2+k j)(3+k^2+j^2-k j)}{.} \end{aligned}$$

In order to highlight the fact that we are working close to zero, we introduce a small parameter $\varepsilon >0$ and we rescale $I\mapsto \varepsilon ^2 I$, so that the *frequency–amplitude map* can be written as

4.5 $$\begin{aligned} \omega (I)\;\rightsquigarrow \; \alpha (I)=\overline{\omega }+\varepsilon ^2\mathbb {A}\,I+O(\varepsilon ^4){,} \end{aligned}$$

where $\overline{\omega }$ is the vector of the linear frequencies (see (1.10)),

4.6 $$\begin{aligned} \mathbb {A}:= & {} \frac{1}{2}\mathbb {D}\,\,\,\mathrm {diag} \left( \frac{{\lambda }(2 j)}{2 {\lambda }(j)-{\lambda }(2 j)}\right) _{j\in S^+}+\mathbb {D}\,\,\,\mathbb {B}{,} \quad \mathbb {D}:=\mathrm {diag} \big ({\lambda }(j)\big )_{j\in S^+}{,}\nonumber \\ \mathbb {B}_j^k:= & {} {\left\{ \begin{array}{ll} \mathtt {b}_{j k}\quad \,\,\text{ if }\,\,\,\,j\ne k{,}\\ 0 \qquad \,\, \text{ if }\,\,\,j=k{.} \end{array}\right. } \end{aligned}$$

The submanifold $\{z=0\}$ is foliated by tori, parameterized by the actions, supporting small amplitude quasi-periodic solutions for the truncated system with Hamiltonian $\mathcal {H}^{(\le 8)}$. We shall select some of them as starting point of the Nash–Moser scheme, by fixing $I=\xi$ (here $\xi$ is a parameter), so that appropriate non-resonance conditions on the frequency $\alpha (I)$ hold.

In order to work in a small neighbourhood of the prefixed torus $\{ I\equiv \xi \}$ it is advantageous to introduce a set of coordinates $(\theta , y, z)\in \mathbb {T}^{\nu }\times \mathbb {R}^{\nu }\times H_S^{\perp }$ adapted to it, defined by

4.7 $$\begin{aligned}&u=A_{\varepsilon }(\theta , y, z)= \varepsilon v_\varepsilon (\theta ,y)+\varepsilon ^{b}z\nonumber \\&\quad \Longleftrightarrow \qquad {\left\{ \begin{array}{ll} u_j:=\varepsilon \sqrt{ \xi _j+\varepsilon ^{2b-2}|{\lambda }(j) |y_j }\, e^{\mathrm {i}\theta _j}\,e^{\mathrm {i}\,j\,x}{,} \,\,\quad j\in S{,}\\ u_j:=\varepsilon ^b z_j{,} \qquad \qquad \qquad \qquad \qquad \qquad j\in S^c{,} \end{array}\right. } \end{aligned}$$

with $b>1$ and where (recall $\overline{u}_j=u_{-j}$)

$$\begin{aligned} \xi _{-j}=\xi _j{,} \quad \xi _j>0{,} \quad y_{-j}=y_{j}{,} \quad \theta _{-j}=-\theta _j{,} \quad \theta _j\in \mathbb {T}{,}\,\, y_j\in \mathbb {R}{,} \quad \forall j\in S{.} \end{aligned}$$

The parameter *b* will be chosen close to one, to this purpose we shall set

4.8 $$\begin{aligned} a := 2b -2{,} \end{aligned}$$

and fix $a>0$ appropriately small. For the tangential sites $S^+:=\{ \overline{\jmath }_1,\ldots , \overline{\jmath }_{\nu }\}$ we will also denote $\theta _{\overline{\jmath }_i}:=\theta _i$, $y_{\overline{\jmath }_i}:=y_{i}$, $\xi _{\overline{\jmath }_i}:=\xi _i$, $i=1,\ldots , \nu$. The symplectic 2-form $\Omega$ in (2.1), up to rescaling of time, becomes

4.9 $$\begin{aligned} \mathcal {W}:=\sum _{i=1}^{\nu } d\theta _i\wedge d y_i+\frac{1}{2} \sum _{j\in S^c} \frac{1}{\mathrm {i} {\lambda }(j)}\,d z_j\wedge d z_{-j}=\Big ( \sum _{i=1}^{\nu } d \theta _i\wedge d y_i \Big ) \oplus \Omega _{S^{\perp }}{,} \end{aligned}$$

where $\Omega _{S^{\perp }}$ is the symplectic form $\Omega$ in (2.1) restricted to the subspace $H_{S}^{\perp }$ in (3.2). The Hamiltonian system generated by $\mathcal {H}$ in (3.8) becomes

4.10 $$\begin{aligned} H_{\varepsilon }:=\varepsilon ^{-2 b}\,\mathcal {H}\circ A_{\varepsilon }{.} \end{aligned}$$

In the following lemma we prove that, under an appropriate choice of the tangential set (1.9), the function (4.5) is a diffeomorphism for $\varepsilon$ small enough and then the system (4.2) is integrable and non-isochronous.

### Lemma 4.1

(Twist condition). There exist $\mathtt {r}_0, \mathtt {c}_{*}>0$ such that, for any choice of the tangential sites $S^+\in \mathcal {V}(\mathtt {r})$ with $0<\mathtt {r}\le \mathtt {r}_0$ (see Definition 1.1), one has $|\det \mathbb {A} |\ge \mathtt {c}_* \,\, \overline{\jmath }_1^{3 \nu }{.}$

### Proof

The proof is postponed in “Appendix A”. $\quad \square$

As a consequence of the non-degeneracy condition in Lemma 4.1 the map in (4.5) is invertible and we denote

4.11 $$\begin{aligned} \xi :=\xi (\omega ) := \alpha ^{(-1)}(\omega ) = \varepsilon ^{-2}\mathbb A^{-1}(\omega -\overline{\omega }) +O( \varepsilon ^2){.} \end{aligned}$$

## The Nonlinear Functional Setting

We write the Hamiltonian in (4.10) (possibly eliminating constant terms depending only on $\xi$ which are irrelevant for the dynamics) as

5.1 $$\begin{aligned} H_{\varepsilon }&=\mathcal {N}+P{,} \nonumber \\ \mathcal {N}(\theta , y, z)&=\omega \cdot y+\frac{1}{2} (N(\theta ) z, z)_{L^2}{,} \quad \frac{1}{2}(N(\theta ) z, z)_{L^2}\!:=\! \frac{1}{2}((\mathtt {D}_z \nabla _{{z}} H_{\varepsilon }) (\theta , 0, 0)[z], z)_{L^2}{,} \end{aligned}$$

where $\mathcal {N}$ describes the linear dynamics normal to the torus, and $P:=H_{\varepsilon }-\mathcal {N}$ collects the nonlinear perturbative effects. Note that both *N* and *P* depend on $\omega$ through the map $\omega \mapsto \xi (\omega )$.

We consider $H_{\varepsilon }$ as a $(\omega , \varepsilon )$-parameter family of Hamiltonians and we note that, for $P=0$, $H_{\varepsilon }$ possess an invariant torus at the origin with frequency $\omega$, which we want to continue to an invariant torus for the full system.

We will select the frequency parameters from the following set (recall (4.11))

5.2 $$\begin{aligned} \Omega _{\varepsilon }:=\{\omega \in \mathbb {R}^\nu \,: \,\, \xi (\omega )\in [1, 2]^{\nu }\}{.} \end{aligned}$$

Setting (see (4.7))

5.3 $$\begin{aligned} \gamma =\varepsilon ^{2b}{,} \quad \tau :=2\nu +6, \end{aligned}$$

we define the non-resonant sets

5.4 $$\begin{aligned} \mathcal {G}^{(0)}_0&:= \big \{ \omega \in \Omega _{\varepsilon } : |\omega \cdot \ell |\ge \gamma \,\langle \ell \rangle ^{-\tau }, \,\, \forall \ell \in \mathbb {Z}^{\nu }{\setminus }\{0\}\big \}{,} \end{aligned}$$

5.5 $$\begin{aligned} \mathcal {G}_0^{(1)}&:= \Big \{ \omega \in \Omega _{\varepsilon } : |\overline{\omega }\cdot \ell +\varepsilon ^2 \mathbb {A}\xi (\omega )\cdot \ell + {\lambda }(j')-{\lambda }(j)+\varepsilon ^2 ({\lambda }(j')\mathfrak {l}_{j'}-{\lambda }(j)\mathfrak {l}_j)|> C\gamma ,\nonumber \\&\quad \sum _{i=1}^{\nu } \overline{\jmath }_i \ell _i+j'-j=0,\,\, \forall |\ell |\le 3,\,\, \ell \in \mathbb {Z}^{\nu }{\setminus }\{0\}\,\,j, j'\in S^c, \,\,\, (\ell , j, j')\!\ne \! (0, j, j)\Big \}{,} \end{aligned}$$

for some constant *C* depending on *S*, where $\mathbb {A}$ is defined in (4.6) and

5.6 $$\begin{aligned} \mathfrak {l}_j:=\frac{2}{3} \sum _{j_2\in S^+} \, \frac{(1+j_2^2)(1+j^2)(2+j_2^2+j^2)}{(3+j_2^2-j_2 j+j^2) (3+j_2^2+j_2 j +j^2)}\xi _{j_2}(\omega ){.} \end{aligned}$$

We require that

5.7 $$\begin{aligned} \omega \in \mathcal {G}_0:=\mathcal {G}^{(0)}_0\cap \mathcal {G}^{(1)}_0{.} \end{aligned}$$

### Lemma 5.1

We have that $|\Omega _{\varepsilon }{\setminus }\mathcal {G}_0 |\le C_{*}\varepsilon ^{2(\nu -1)} \gamma \,$ for some $C_*=C_*(S)>0\,$.

### Proof

The proof is postponed in “Appendix A”. $\quad \square$

### Remark 5.2

The diophantine condition $\omega \in \mathcal {G}_0^{(0)}$ is typical of KAM scheme. The lower bound in $\mathcal {G}_0^{(1)}$ involves resonances of order five with two normal modes. As explained in the introduction, in order to impose such lower bounds we need to take into account also the corrections of order $\varepsilon ^{2}$. The matrix $\mathbb {A}$ comes from the weak BNF of Sect. 3. The terms $\mathfrak {l}_{j}$ come from the *linear* BNF procedure of Sect. 7.2. In particular they are evaluated explicitly using the identification argument of Theorem 7.9.

### Remark 5.3

Note that the definition of $\gamma$ in (5.3) is slightly stronger than the minimal condition for which is possible to prove that $\mathcal {G}_0^{(0)}$ has large measure, namely $\gamma \le c\,\varepsilon ^2$, with $c>0$ small enough. Our choice turns out to be useful for proving that the Cantor set of frequencies of the expected quasi-periodic solutions has asymptotically full measure (as $\varepsilon \rightarrow 0$).

We look for an embedded invariant torus

5.8 $$\begin{aligned} i :\mathbb {T}^{\nu }\rightarrow \mathbb {T}^{\nu }\times \mathbb {R}^{\nu }\times H_S^{\perp }{,} \quad \varphi \mapsto i(\varphi ):=(\theta (\varphi ), y(\varphi ), z(\varphi )) \end{aligned}$$

of the Hamiltonian vector field $X_{H_{\varepsilon }}$ (see (5.1)) supporting quasi-periodic solutions with diophantine frequency $\omega \in \mathcal {G}_0$.

For technical reason, it is useful to consider the modified Hamiltonian

5.9 $$\begin{aligned} H_{\varepsilon , \zeta }(\theta , y, z):=H_{\varepsilon }(\theta , y, z) +\zeta \cdot \theta , \quad \zeta \in \mathbb {R}^{\nu }{.} \end{aligned}$$

More precisely, we introduce $\zeta$ in order to control the average in the *y*-component in our Nash Moser scheme. The vector $\zeta$ has no dynamical consequences since an invariant torus for the Hamiltonian vector field $X_{H_{\varepsilon , \zeta }}$ is actually invariant for $X_{H_{\varepsilon }}$ itself.

Thus, we look for zeros of the nonlinear operator $\mathcal {F}(i, \zeta )\equiv \mathcal {F}(i, \zeta , \omega , \varepsilon ):= \omega \cdot \partial _{\varphi } i(\varphi )-X_{\mathcal {N}}(i(\varphi ))-X_P(i(\varphi ))+(0, \zeta , 0)$ defined as

5.10 $$\begin{aligned} \mathcal {F}(i, \zeta ) =\begin{pmatrix} \omega \cdot \partial _{\varphi } \Theta (\varphi )-\partial _y P(i(\varphi ))\\ \omega \cdot \partial _{\varphi } y(\varphi )+\frac{1}{2} \partial _{\theta } (N(\theta (\varphi ))z(\varphi ))_{L^2(\mathbb {T})}+\partial _{\theta } P(i(\varphi ))+\zeta \\ \omega \cdot \partial _{\varphi } z(\varphi )-J N(\theta (\varphi ))\,z(\varphi )-J\nabla _z P(i(\varphi )) \end{pmatrix} \end{aligned}$$

where $\Theta (\varphi ):=\theta (\varphi )-\varphi$ is $(2\pi )^{\nu }$-periodic. We define the Sobolev norm of the periodic component of the embedded torus

5.11 $$\begin{aligned} \mathfrak {I}(\varphi ):=i(\varphi )-(\varphi , 0, 0):=(\Theta (\varphi ), y(\varphi ), z(\varphi )) \;\quad \Vert \mathfrak {I} \Vert _{s}:=\Vert \Theta \Vert _{s}+\Vert y \Vert _{s}+\Vert z \Vert _s, \end{aligned}$$

where $z \in H^s_{S^{\perp }}:= H^s \cap H_{S}^\perp$ (recall (3.2)) with norm defined in (2.7) and with abuse of notation, we are denoting by $\Vert \cdot \Vert _{s}$ the Sobolev norms of functions in $H^{s}(\mathbb {T}^{\nu },\mathbb {R}^{\nu })$. From now on we fix $s_0:=[\nu /2]+4$.

Notice that in the coordinates (4.7), a quasi-periodic solution corresponds to an embedded invariant torus (5.8). Therefore we can reformulate the main Theorem 1 as follows.

### Theorem 5.4

There exists a small constant $\mathtt {r}>0$ such that, for any $S^{+}\in \mathcal {V}(\mathtt {r})$ (see (1.9) and Definition 1.1), there exists $\varepsilon _0>0$, small enough, such that the following holds. For all $\varepsilon \in (0, \varepsilon _0)$ there exist positive constants $C=C(\nu )$, $\mu =\mu (\nu )$ and a Cantor-like set $\mathcal {C}_{\varepsilon }\subseteq \Omega _{\varepsilon }$ (see (5.2)), with asymptotically full measure as $\varepsilon \rightarrow 0$, namely

5.12 $$\begin{aligned} \lim _{\varepsilon \rightarrow 0} \dfrac{|\mathcal {C}_{\varepsilon } |}{|\Omega _{\varepsilon } |}=1{,} \end{aligned}$$

such that, for all $\omega \in \mathcal {C}_{\varepsilon }$, there exists a solution $i_{\infty }(\varphi ):=i_{\infty }(\omega , \varepsilon )(\varphi )$ of the equation $\mathcal {F}(i_{\infty }, 0, \omega , \varepsilon )=0$ (see (5.10)). Hence the embedded torus $\varphi \mapsto i_{\infty }(\varphi )$ is invariant for the Hamiltonian vector field $X_{H_{\varepsilon }}$, and it is filled by quasi-periodic solutions with frequency $\omega$. The torus $i_{\infty }$ satisfies

$$\begin{aligned} \Vert i_{\infty }(\varphi )-(\varphi , 0, 0) \Vert _{s_0+\mu }^{\gamma , \mathcal {C}_{\varepsilon }}\le C\,\varepsilon ^{9-2 b}\,\gamma ^{-1}{.} \end{aligned}$$

Moreover the torus $i_{\infty }$ is linearly stable.

We can deduce Theorem 1 from Theorem 5.4, indeed the quasi-periodic solution *u* in (1.11) is

$$\begin{aligned} u(t, x)=\Big (\Phi _B\circ A_{\varepsilon }\Big ) i_{\infty } (\omega t) \end{aligned}$$

for $\omega =\omega (\xi )\in \mathcal {C}_{\varepsilon }$, where $\omega (\xi )$ is the frequency amplitude map (4.5).

The rest of the paper is devoted to the proof of Theorem 5.4.

### Tame estimates of the nonlinear vector field

We give tame estimates for the composition operator induced by the Hamiltonian vector fields $X_{\mathcal {N}}$ and $X_{P}$ in (5.10). Since the functions $y\rightarrow \sqrt{\xi +\varepsilon ^{2(b-1)} y}, \theta \rightarrow e^{\mathrm {i}\,\theta }$ are analytic for $\varepsilon$ small enough and $|y |\le C$, classical composition results (see for instance Lemma 6.2 in [3]) imply that, for all $\Vert \mathfrak {I} \Vert _{s_0}^{\gamma , \mathcal {O}}\le 1$,

$$\begin{aligned} \Vert A_{\varepsilon }(\theta (\varphi ), y(\varphi ), z(\varphi )) \Vert _s^{\gamma , \mathcal {O}}\lesssim _s \varepsilon (1+\Vert \mathfrak {I} \Vert _s^{\gamma , \mathcal {O}}){.} \end{aligned}$$

In the following lemma we collect tame estimates for the Hamiltonian vector fields $X_{\mathcal {N}}, X_{P}, X_{H_{\varepsilon }}$, see (5.1). These bounds rely on tame estimates for composition operators and their proof is completely analogous to the one in Sect. 5 of [4].

#### Lemma 5.5

Let $\mathfrak {I}(\varphi )$ in (5.11) satisfy $\Vert \mathfrak {I} \Vert _{s_0+1}^{\gamma , \mathcal {O}}\lesssim \,\varepsilon ^{9-2 b}\gamma ^{-1}$. Then we have

5.13 $$\begin{aligned} \Vert \partial _y P(i) \Vert _s^{\gamma , \mathcal {O}}&\lesssim _s \varepsilon ^7+\varepsilon ^{2 b} \Vert \mathfrak {I} \Vert _{s+1}^{\gamma , \mathcal {O}}{,} \nonumber \\ \Vert \partial _{\theta } P(i) \Vert _s^{\gamma , \mathcal {O}}&\lesssim _s \varepsilon ^{9-2 b}(1+\Vert \mathfrak {I} \Vert _{s+1}^{\gamma , \mathcal {O}}){,} \nonumber \\ \Vert \nabla _z P(i) \Vert _s^{\gamma , \mathcal {O}}&\lesssim _s \varepsilon ^{8-b}+\varepsilon ^{9-b}\gamma ^{-1} \Vert \mathfrak {I} \Vert _{s+1}^{\gamma , \mathcal {O}}{,} \nonumber \\ \Vert X_P (i) \Vert _s^{\gamma , \mathcal {O}}&\lesssim _s \varepsilon ^{9-2 b}+\varepsilon ^{2 b} \Vert \mathfrak {I} \Vert _{s+1}^{\gamma , \mathcal {O}}{,} \nonumber \\ \Vert \partial _{\theta }\partial _y P(i) \Vert _s^{\gamma , \mathcal {O}}&\lesssim _s \varepsilon ^7+\varepsilon ^8\gamma ^{-1} \Vert \mathfrak {I} \Vert _{s+1}^{\gamma , \mathcal {O}}{,}\nonumber \\ \Vert \partial _y\nabla _z P(i) \Vert _s^{\gamma , \mathcal {O}}&\lesssim _s \varepsilon ^{6+b}+\varepsilon ^{2 b-1} \Vert \mathfrak {I} \Vert _{s+1}^{\gamma , \mathcal {O}}{,}\nonumber \\ \Vert \partial _{y y} P(i)-\frac{\varepsilon ^{2 b}}{2} \mathbb {A}\Omega \Vert _s^{\gamma , \mathcal {O}}&\lesssim _s \varepsilon ^{5+2 b}+\varepsilon ^{6+2 b}\gamma ^{-1} \Vert \mathfrak {I} \Vert _{s+1}^{\gamma , \mathcal {O}}{,} \end{aligned}$$

and for all $\widehat{\imath }:=(\widehat{\Theta }, \widehat{y}, \widehat{z})$,

$$\begin{aligned} \Vert \partial _y \mathtt {D}_i X_P(i)[\widehat{\imath }] \Vert _s^{\gamma , \mathcal {O}}&\lesssim _s \varepsilon ^{2 b-1} (\Vert \widehat{\imath } \Vert _{s+1}^{\gamma , \mathcal {O}} +\Vert \mathfrak {I} \Vert _{s+1}^{\gamma , \mathcal {O}}\Vert \widehat{\imath } \Vert ^{{\gamma }, \mathcal {O}}_{s_0+1}){,}\\ \Vert \mathtt {D}_i X_{H_{\varepsilon }}(i)[\widehat{\imath }]+(0, 0, J\, \widehat{z})\Vert _s^{\gamma , \mathcal {O}}&\lesssim _s \varepsilon (\Vert \widehat{\imath } \Vert _{s+1}^{\gamma , \mathcal {O}} +\Vert \mathfrak {I} \Vert _{s+1}^{\gamma , \mathcal {O}}\Vert \widehat{\imath } \Vert ^{{\gamma }, \mathcal {O}}_{s_0+1}){,}\\ \Vert \mathtt {D}_i^2 X_{H_{\varepsilon }} (i) [\widehat{\imath }, \widehat{\imath }]\Vert _s^{\gamma , \mathcal {O}}&\lesssim _s \varepsilon (\Vert \widehat{\imath } \Vert _{s+1}^{\gamma , \mathcal {O}}\Vert \widehat{\imath } \Vert _{s_0+1}^{\gamma , \mathcal {O}} +\Vert \mathfrak {I} \Vert _{s+1}^{\gamma , \mathcal {O}}(\Vert \widehat{\imath } \Vert ^{{\gamma }, \mathcal {O}}_{s_0+1})^2){.} \end{aligned}$$

In the sequel we will use that, by the diophantine condition (5.7), the operator $(\omega \cdot \partial _{{\varphi }})^{-1}$ is defined for all functions *u* with zero $\varphi$-average, and satisfies

$$\begin{aligned} \Vert (\omega \cdot \partial _{\varphi })^{-1} u \Vert _s \lesssim _s \gamma ^{-1}\,\Vert u \Vert _{s+\tau }, \quad \Vert (\omega \cdot \partial _{\varphi })^{-1} u \Vert _s^{\gamma , \mathcal {O}}\lesssim _s \gamma ^{-1} \Vert u \Vert ^{\gamma , \mathcal {O}}_{s+2\tau +1}{.} \end{aligned}$$

## Approximate Inverse

We want to solve the nonlinear functional equation (see (5.10))

6.1 $$\begin{aligned} \mathcal {F}(i, \zeta )=0 \end{aligned}$$

by applying a Nash–Moser scheme. It is well known that the main issue in implementing this algorithm concerns the approximate inversion of the linearized operator of $\mathcal {F}$ at any approximate solution $(i_n, \zeta _n)$, namely $\mathtt {D}\mathcal {F}(i_n, \zeta _n)$. Note that $\mathtt {D}\mathcal {F}(i_n, \zeta _n)$ is independent of $\zeta _n$. One of the main problems is that the $(\theta , y, z)$-components of $\mathtt {D}\mathcal {F}(i_n, \zeta _n)$ are coupled and then the linear system

6.2 $$\begin{aligned} \mathtt {D}\mathcal {F}(i_n, \zeta _n)[\widehat{\imath }, \widehat{\zeta }]=\omega \cdot \partial _{\varphi } \widehat{\imath }-\mathtt {D}_{i} X_{H_\varepsilon } (i_n)[\widehat{\imath }]-(0, \widehat{\zeta }, 0)=g=(g^{(\theta )}, g^{(y)}, g^{(z)}) \end{aligned}$$

is quite involved. In order to approximately solve (6.2) we follow the scheme developed by Berti–Bolle in [9] which describe a way to approximately triangularize (6.2). This method has been applied in [4, 39]. Since the strategy is identical to [39] we only summarize it and underline the differences which mainly come from the symplectic structure. For a fully detailed expository presentation see [40].

We now study the solvability of Eq. (6.2) at an approximate solution, which we denote by $(i_0, \zeta _0)$, $i_0(\varphi )=(\theta _0(\varphi ), y_0(\varphi ), z_0(\varphi ))$ in order to keep the notations of [4, 39] . Assume the following hypothesis, which we shall verify at any step of the Nash–Moser iteration,

- **Assumption** The map $\omega \mapsto i_0(\omega )$ is a Lipschitz function defined on some subset $\mathcal {O}_0\subseteq \mathcal {G}_0 \subseteq \Omega _{\varepsilon }$ (recall (5.7),(5.2)) and, for some $\mathfrak {p}_0:=\mathfrak {p}_0( \nu )>0$,
  6.3 $$\begin{aligned} \Vert \mathfrak {I}_0 \Vert _{s_0+\mathfrak {p}_0}^{\gamma , \mathcal {O}_0}\le \varepsilon ^{9-2b} \gamma ^{-1}, \quad \Vert Z \Vert _{s_0+\mathfrak {p}_0}^{\gamma , \mathcal {O}_0}\le \varepsilon ^{9-2 b}, \quad \gamma =\varepsilon ^{2b}, \end{aligned}$$
  where $\mathfrak {I}_0(\varphi ):=i_0(\varphi )-(\varphi , 0, 0)$ and *Z* is the error function
  6.4 $$\begin{aligned} Z(\varphi ):=(Z_1, Z_2, Z_3)(\varphi ):=\mathcal {F}(i_0, \zeta _0)(\varphi )=\omega \cdot \partial _{\varphi } i_0 (\varphi )-X_{H_{\varepsilon , \zeta _0}}(i_0(\varphi )). \end{aligned}$$

By estimating the Sobolev norm of the function *Z* we can measure how the embedding $i_0$ is close to being invariant for $X_{H_{\varepsilon , \zeta _0}}$. If $Z=0$ then $i_0$ is a solution. In general we say that $i_0$ is “approximately invariant” up to order *O*(*Z*). We observe that by Lemma 6.1 in [4] we have that if $i_0$ is a solution, then the parameter $\zeta _0$ has to be naught, hence the embedded torus $i_0$ supports a quasi-periodic solution of the “original” system with Hamiltonian $H_{\varepsilon }$ (see (5.1)).

By [9] we know that it is possible to construct an embedded torus $i_{\delta }(\varphi )=(\theta _0(\varphi ), y_{\delta }(\varphi ), z_0(\varphi ))$, which differs from $i_0$ only for a small modification of the *y*-component, such that the 2-form $\mathcal {W}$ (recall (4.9)) vanishes on the torus $i_{\delta }(\mathbb {T}^{\nu })$, namely $i_{\delta }$ is isotropic. In particular $i_{\delta }(\varphi )$ is approximately invariant up to order *O*(*Z*) (see Lemma 7 in [9]) and, more precisely, there exists $\tilde{\mathfrak {p}}:=\tilde{\mathfrak {p}}(\nu )>0$ such that

6.5 $$\begin{aligned} \Vert i_{\delta }-i_0 \Vert ^{{\gamma }, \mathcal {O}_0}_s\lesssim _s \Vert \mathfrak {I}_0 \Vert ^{{\gamma }, \mathcal {O}_0}_{s+\tilde{\mathfrak {p}}}. \end{aligned}$$

The strategy is to construct an approximate inverse for $\mathtt {D}\mathcal {F}(i_0, \zeta _0)$ by starting from an approximate inverse for the linear operator $\mathtt {D}\mathcal {F}(i_\delta , \zeta _0)$. The advantage of analyzing the linearized problem at $i_{\delta }$ is that it is possible to construct a *symplectic* change of variable which approximately triangularizes the linear system thanks to the isotropicity of $i_{\delta }$. For the details we refer to [9] and [4], here we only give the relevant definitions and state the main result. We define the symplectic change of coordinates

6.6 $$\begin{aligned} \begin{pmatrix} \theta \\ y \\ z \end{pmatrix} :=G_{\delta }\begin{pmatrix} \varphi \\ \eta \\ w \end{pmatrix} :=\begin{pmatrix} \theta _0(\varphi ) \\ y_{\delta }(\varphi )+[\partial _{\varphi }\theta _0(\varphi )]^{-T}\eta +[(\partial _{\theta } \tilde{z}_0)(\theta _0(\varphi ))]^T\,J^{-1}w\\ z_0(\varphi )+w \end{pmatrix} \end{aligned}$$

where $\tilde{z}_0:=z_0 (\theta _0^{-1} (\theta ))$. We denote the transformed Hamiltonian by $K:=K(\varphi , \eta , w, \zeta _0)$. We then define

6.7 $$\begin{aligned} \mathcal {L}_{\omega }:=\omega \cdot \partial _{\varphi }-J K_{02}(\varphi ){,} \end{aligned}$$

where $K_{02}$ is the linear operator representing the terms quadratic in *w* of *K*, i.e.

6.8 $$\begin{aligned} \frac{1}{2} (K_{02}(\varphi )[w],w):= \Pi ^{d_w=2}K = \Pi ^{d_w=2} H_\varepsilon \circ G_\delta {.} \end{aligned}$$

$\mathcal {L}_{\omega }$ corresponds to the *w*-component of the linearized operator after the change of variable $G_\delta$.

In [9] (see also [4, 39]) the following result is proved.

### Theorem 6.1

Assume (6.3) and the following

**Inversion Assumption** There exist $\mathfrak {p}_1:=\mathfrak {p}_1(\nu )>0$ and a set $\Omega _{\infty }\subset \mathcal {G}_0\subseteq \Omega _{\varepsilon }$ such that for all $\omega \in \Omega _{\infty }$ and every function $g\in H^{s+2\tau +1} \cap H_{S}^\perp$, there exists a solution $h:=\mathcal {L}_{\omega }^{-1} g$ of the linear equation $\mathcal {L}_{\omega } h=g$ which satisfies

6.9 $$\begin{aligned} \Vert \mathcal {L}_{\omega }^{-1} g \Vert _s^{\gamma , \Omega _{\infty }} \lesssim _s \gamma ^{-1} (\Vert g \Vert _{s+2\tau +1}^{\gamma , \Omega _{\infty }} +\varepsilon \gamma ^{-5/2} \Vert \mathfrak {I}_\delta \Vert _{s+\mathfrak {p}_1}^{\gamma , \mathcal {O}_0} \Vert g \Vert _{s_0}^{\gamma , \Omega _{\infty }}){.} \end{aligned}$$

Then there exists $\mu :=\mu ( \nu )$ such that, for all $\omega \in \Omega _{\infty }$ there exists a linear operator $\mathbf {T}_0$ such that:

1. For all $g:=(g^{(\theta )}, g^{(y)}, g^{(z)})$, one has
  6.10 $$\begin{aligned} \Vert \mathbf {T}_0 g \Vert _s^{\gamma , \Omega _{\infty }}\lesssim _s\gamma ^{-1}(\Vert g \Vert _{s+\mu }^{\gamma , \Omega _{\infty }} +\varepsilon \gamma ^{-5/2} \Vert \mathfrak {I}_0 \Vert _{s+\mu }^{\gamma , \mathcal {O}_0} \Vert g \Vert _{s_0+\mu }^{\gamma , \Omega _{\infty }}){.} \end{aligned}$$
2. $\mathbf {T}_0$ is an approximate inverse of $\mathtt {D}\mathcal {F}(i_0)$, namely
  6.11 $$\begin{aligned}&\Vert (\mathtt {D}\mathcal {F}(i_0)\circ \mathbf {T}_0-\mathrm {I}) g \Vert _s^{\gamma , \Omega _{\infty }} \lesssim _s \varepsilon ^{2 b-1}{\gamma }^{-2} \Big ( \Vert \mathcal {F}(i_0, \zeta _0) \Vert _{s_0+\mu }^{\gamma , \mathcal {O}_0} \Vert g \Vert _{s+\mu }^{\gamma , \Omega _{\infty }}\nonumber \\&\qquad \qquad \qquad +\{\Vert \mathcal {F}(i_0, \zeta _0)\Vert _{s+\mu }^{\gamma , \mathcal {O}_0} +\varepsilon \gamma ^{-5/2} \Vert \mathcal {F}(i_0, \zeta _0) \Vert _{s_0+\mu }^{\gamma , \mathcal {O}_0} \Vert \mathfrak {I}_0 \Vert _{s+\mu }^{\gamma , \mathcal {O}_0}\} \Vert g \Vert _{s_0+\mu }^{\gamma , \Omega _{\infty }} \Big ){.} \end{aligned}$$

### The linearized operator in the normal directions

Recalling the assumption (6.3), in the sequel we assume that $\mathfrak {I}_{\delta }:=\mathfrak {I}_{\delta }(\varphi ; \omega ) =i_{\delta }(\varphi ;\, \omega )-(\varphi ,\, 0,\, 0)$ satisfies, for some $\mathfrak {p}_1>0$,

6.12 $$\begin{aligned} \Vert \mathfrak {I}_{\delta } \Vert _{s_0+\mathfrak {p}_1}^{{\gamma },\mathcal {O}_0} \lesssim \,\varepsilon ^{9-2 b}\gamma ^{-1}{.} \end{aligned}$$

We note moreover that $G_\delta$ in (6.6) is the identity plus a translation plus a finite rank linear operator; moreover, assuming (6.12), one has that $G_{\delta }$ is $O(\varepsilon ^{9-2 b}\gamma ^{-1})$-close to the identity in low norm. Returning to the initial variables we set (see (4.7),(6.6))

6.13 $$\begin{aligned} T_{\delta }:=A_{\varepsilon }(G_{\delta }(\varphi , 0, 0)) = \varepsilon v_\delta +\varepsilon ^b z_0{,} \quad v_\delta = \sum _{j\in S} \sqrt{\xi _j + \varepsilon ^{2b-2}|\lambda (j)|y_{\delta j}(\varphi )} e^{\mathrm {i} (j x + \theta _{0 j}(\varphi )) } \end{aligned}$$

and we have, for some $\sigma :=\sigma (\nu )>0$,

6.14 $$\begin{aligned} \Vert \Phi _B(T_{\delta }) \Vert _s^{\gamma , \mathcal {O}_0} \lesssim _s \varepsilon \, (1+\Vert \mathfrak {I}_{\delta } \Vert ^{\gamma , \mathcal {O}_0}_{s+\sigma }){,} \qquad \Vert \mathtt {D}_i \Phi _B(T_{\delta }) [\widehat{\imath }] \Vert _s \lesssim _s \varepsilon (\Vert i\Vert _{s+\sigma } + \Vert \mathfrak {I}_{\delta } \Vert _{s+\sigma }\Vert i \Vert _{s_0+\sigma }){.} \end{aligned}$$

By following Sect. 7 in [4] (see Lemma 7.1), $K_{02}$ in (6.8) has rather explicit estimates.

#### Proposition 6.2

Assume (6.12). Then there exists $\sigma _0=\sigma _0(\nu )>0$ such that the following holds. The Hamiltonian operator $\mathcal {L}_{\omega }$ in (6.7) has the form

6.15 $$\begin{aligned} \mathcal {L}_{\omega }=\Pi _S^{\perp }\big (\omega \cdot \partial _{\varphi }-J\circ (1+a_0(\varphi , x))+\mathcal {Q}_0\big ), \qquad a_0(\varphi , x):=-(\Phi _B(T_{\delta })+\partial _{u}^{2}f(\Phi _{B}(T_{\delta }))){.} \end{aligned}$$

Recall that $T_{\delta }$ is defined in (6.13), $\Phi _B$ is the Birkhoff map given in Proposition 3.2, *f* is the Hamiltonian density in (1.3). The operator $\mathcal {Q}_0$ is finite rank and has the form

6.16 $$\begin{aligned} \mathcal {Q}_0(\varphi ) w=\sum _{|j |\le C} \int _0^1 (w, g_j(\tau , \varphi ))_{L^2(\mathbb {T})}\,\chi _j(\tau , \varphi )\,d\tau . \end{aligned}$$

In particular we divide $\mathcal {Q}_0= \sum _{i=1}^5 \varepsilon ^i\mathcal {R}_i +\mathcal {R}_{>5}$, where the $\mathcal {R}_i$, $\mathcal {R}_{>5}$ are finite rank operators. Moreover we have

6.17 $$\begin{aligned} \Vert a_0 \Vert _{s}^{\gamma , \mathcal {O}_0}\lesssim _s \varepsilon (1+\Vert \mathfrak {I}_{\delta }\Vert ^{\gamma , \mathcal {O}_0}_{s+\sigma _0}){,} \qquad \Vert \mathtt {D}_i a_0 [\widehat{\imath }] \Vert _s\lesssim _s \varepsilon (\Vert \widehat{\imath } \Vert _{s+\sigma _0} +\Vert \mathfrak {I}_{\delta }\Vert _{s+\sigma _0}\Vert \widehat{\imath }\Vert _{s_0}){.} \end{aligned}$$

The remainders $\mathcal {R}_i$ do not depend on $\mathfrak {I}_\delta$ and satisfy

6.18 $$\begin{aligned} \Vert g^{(i)}_j \Vert _s^{\gamma , \mathcal {O}_0} +\Vert \chi _j^{(i)} \Vert _s^{\gamma , \mathcal {O}_0}\lesssim _s 1{,} \end{aligned}$$

while $$\mathcal {R}_{>5}$$ satisfies

6.19 $$\begin{aligned}&\Vert g^{>5}_j \Vert _s^{\gamma , \mathcal {O}_0} \Vert \chi _j^{>5} \Vert _{s_0}^{\gamma , \mathcal {O}_0} +\Vert g^{>5}_j \Vert _{s_0}^{\gamma , \mathcal {O}_0} \Vert \chi _j^{>5} \Vert _{s}^{\gamma , \mathcal {O}_0}\lesssim _s \varepsilon ^6+\varepsilon ^{2}\Vert \mathfrak {I}_{\delta } \Vert _{s+\sigma }^{\gamma , \mathcal {O}_0}{,} \end{aligned}$$

6.20 $$\begin{aligned}&\Vert \mathtt {D}_i g^{>5}_j [\widehat{\imath }] \Vert _s\Vert \chi _j^{>5} \Vert _{s_0} +\Vert \mathtt {D}_i g^{>5}_j [\widehat{\imath }] \Vert _{s_0}\Vert \chi _j^{>5} \Vert _{s} +\Vert g^{>5}_j \Vert _{s_0} \Vert \mathtt {D}_i \chi ^*_j \Vert _{s} +\Vert g^{>5}_j \Vert _{s} \Vert \mathtt {D}_i \chi ^{>5}_j \Vert _{s_0} \nonumber \\&\qquad \qquad \qquad \qquad \lesssim _s \varepsilon ^{2} \Vert \widehat{\imath } \Vert _{s+\sigma } +\varepsilon ^{b}\Vert \mathfrak {I}_{\delta } \Vert _{s+\sigma } \Vert \widehat{\imath } \Vert _{s_0+\sigma }{.} \end{aligned}$$

Finally, recalling the Definition 2.3, we have

6.21 $$\begin{aligned} \mathfrak {M}^{\gamma }_{\mathcal {Q}_0}(0,s)&\lesssim _s \varepsilon ^2 (1+\Vert \mathfrak {I}_{\delta } \Vert ^{\gamma , \mathcal {O}_0}_{s+\sigma _0}){,} \end{aligned}$$

6.22 $$\begin{aligned} \mathfrak {M}_{ \mathtt {D}_i \mathcal {Q}_0 [\widehat{\imath }] }(0,s)&\lesssim _s \varepsilon ^2 \Vert \widehat{\imath } \Vert _{s+\sigma _0}+\varepsilon ^{2b-1} \Vert \mathfrak {I}_{\delta }\Vert _{s+\sigma _0} \Vert \widehat{\imath } \Vert _{s_0+\sigma _0}{.} \end{aligned}$$

#### Proof

The expression (6.15) follows from the definition (6.8) by remarking that $G_\delta$ and the weak BNF transformation $\Phi _B$ is the identity plus a finite rank operator, while the action angle change of coordinates is a rescaling plus a finite rank operator (acting only on the *v*). Then, in applying the chain rule, we get

6.23 $$\begin{aligned} \mathtt {D}_{w} \nabla _w (H_\varepsilon \circ G_\delta )&{\mathop {=}\limits ^{(4.10)}} \varepsilon ^{-2b} (\mathtt {D}_{z} \nabla _z (\mathcal {H}\circ A_\varepsilon ))\circ G_\delta + R_1 = (\mathtt {D}_{z} \nabla _z \mathcal {H})\circ A_\varepsilon \circ G_\delta + R_1 \nonumber \\&{\mathop {=}\limits ^{(3.8)}} (\mathtt {D}_{z} \nabla _z (H\circ \Phi _{B}))\circ A_\varepsilon \circ G_\delta + R_1 \nonumber \\&= (\mathtt {D}_{z} \nabla _z H)\circ \Phi _{B}\circ A_\varepsilon \circ G_\delta + R_1 + R_2 \end{aligned}$$

where the finite rank part contains all the terms where a derivative falls on the change of variables. Then (6.15) follows from the definition of *H* in (1.4). Regarding the estimates, (6.17) follows from (6.14); regarding the bounds (6.18), (6.19), we split the finite rank part $R_1+R_2$ as follows. The operator $R_1$ contains all terms arising form derivatives of $G_\delta$. By tame estimates on the map $G_{\delta }$ (see for instance Lemma 6.7 in [4]), it satisfies the bounds (6.19) and we put it in $\mathcal {R}_{>5}$. The finite rank term $R_2$ comes from the Birkhoff map. This is an analytic map so we consider the Taylor expansion

6.24 $$\begin{aligned} \Phi _B(u)=u+\sum _{i=2}^5\Psi _i(u)+\Psi _{\ge 6}(u){,} \end{aligned}$$

where each $\Psi _i(u)$ is homogeneous of degree *i* in *u*, while $\Psi _{\ge 6}=O(u^6)$ and all map $H^1_0(\mathbb {T})$ in itself. We have to evaluate $\Phi _B$ and its derivatives (up to order two) at $u=T_{\delta }=\varepsilon v_{\delta }+\varepsilon ^b z_0$. We denote by $\overline{v}$ the function

6.25 $$\begin{aligned} \overline{v}(\varphi , x):=\sum _{j\in S} \sqrt{\xi _j} e^{\mathrm {i} (j x+\mathtt {l}(j)\cdot \varphi )}= A_\varepsilon ({\varphi },0,0) \end{aligned}$$

where $\mathtt {l}(\overline{\jmath }_i)$ is the *i*-th vector of the canonical basis of $\mathbb {Z}^{\nu }$ and is such that $\mathtt {l}(-\overline{\jmath }_i)=-\mathtt {l}(\overline{\jmath }_i)$.

We observe that^2^

$$\begin{aligned} \Vert v_{\delta }-\overline{v}\Vert ^{\gamma , \mathcal {O}_0}_s \lesssim \Vert \mathfrak {I}_{\delta }\Vert ^{\gamma , \mathcal {O}_0}_{s}{,} \end{aligned}$$

and hence we can expand

6.26 $$\begin{aligned} \Phi _B(T_{\delta })=\varepsilon \overline{v}+\sum _{i=2}^5\varepsilon ^i\Psi _i( \overline{v}) +\tilde{q}= \Phi _B^{\le 5} + \tilde{q} {,} \end{aligned}$$

where $\tilde{q}$ is a remainder which satisfies

6.27 $$\begin{aligned} \Vert \tilde{q} \Vert _s^{\gamma , \mathcal {O}_0}\lesssim _s \varepsilon ^6+\varepsilon \Vert \mathfrak {I}_{\delta } \Vert _s^{\gamma , \mathcal {O}_0}{,} \quad \Vert \mathtt {D}_i \tilde{q} [\widehat{\imath }] \Vert _s \lesssim _s \varepsilon (\Vert \widehat{\imath } \Vert _s+\Vert \mathfrak {I}_{\delta } \Vert _s \Vert \widehat{\imath } \Vert _{s_0}){.} \end{aligned}$$

Then in $\mathcal {R}_i$ we include all the terms homogeneous of degree *i* coming from derivatives of $\Phi _B-\mathrm {I}$ , evaluated at $\tilde{q}=0$; we put in $\mathcal {R}_{>5}$ all the rest. The (6.21), (6.22) follows by (6.18), (6.19) and (6.20). $\quad \square$

#### Remark 6.3

The motivation for separating the $\mathcal {R}_i$ and $\mathcal {R}_{>5}$ is the following. Consider the Hamiltonian $H_\varepsilon$ as a function of $\xi$ instead of $\omega$. Then in all our expressions we can, and shall, evidence a *purely polinomial* term $\sum _{i=0}^5 \varepsilon ^i f_i$ (where the $f_i$ are $\varepsilon$ independent) plus a *remainder*, which is not analytic in $\varepsilon$, of size $\varepsilon ^6+\varepsilon \Vert \mathfrak {I}_{\delta } \Vert _s^{\gamma , \mathcal {O}_0}$. By the assumption (6.3), this means that in *low norm*$s=s_0+\mathfrak {p}_1$ all these remainders are negligible w.r.t. terms of order $\varepsilon ^5$. This distinction is needed because, due to the resonant nature of the DP equation, we need to perform (see Sects. 7.1 and 7.2) five steps of the order reduction and of the linear BNF by hand, before entering in a perturbative regime.

In this framework $\mathcal {R}_{>5}$ is purely a remainder, while the $\mathcal {R}_i$ are homogeneous polynomial terms. One could apply the same division to the non finite rank terms, one would get

6.28 $$\begin{aligned}&\Pi _S^{\perp } \big (\omega (\xi )\cdot \partial _{\varphi } h -J\,[(1-\Phi _B(T_{\delta })- \partial _{u }^2 f(\Phi _B(T_{\delta })))\nonumber \\&\quad = \Pi _S^{\perp } \big (\overline{\omega }\cdot \partial _{\varphi } h + \varepsilon ^2 \mathbb A\xi \cdot \partial _{\varphi } h -J\,(1-\Phi ^{\le 5}_B(\varepsilon \overline{v}) )h + g h \end{aligned}$$

where *g* satisfies the same estimates as (6.19).

#### Hamiltonian of the linearized operator

Following Remark 6.3, we evidence the terms homogeneous in the Hamiltonian of $\mathcal {L}_{\omega }$, let us call it $\mathsf {H}$, whose Hamiltonian vector fields have degree $\le 5$, since they are NOT perturbative. As explained in (6.26) this entails expanding the map $\Phi _B(T_{\delta })$ in powers of $\varepsilon$ up to order five plus a *small remainder*$\tilde{q}$.

We consider the symplectic form in the extended phase space $({\varphi },\eta ,z)\in \mathbb {R}^{\nu }\times \mathbb {R}^{\nu }\times H_S^{\perp }$

6.29 $$\begin{aligned} \Omega _{e}(\varphi , \eta , z):=d\varphi \wedge d\eta +\sum _{j\in S^c} \frac{1}{\mathrm {i} \lambda (j)} d z_j\wedge d z_{-j} \end{aligned}$$

with the Poisson brackets (recalling $\{ \cdot , \cdot \}$ defined in (2.2))

6.30 $$\begin{aligned} \{ F, G \}_{e}:=\partial _{\varphi } F \partial _{\eta } G -\partial _{\eta } F \partial _{\varphi } G+\{ F, G\}{.} \end{aligned}$$

The Hamiltonian of the operator (6.15) respect to the symplectic form (6.29) is (see (6.28))

6.31 $$\begin{aligned} \mathsf {H}:=\mathsf {H}_0+\sum _{i=1}^5\varepsilon ^i\mathsf {H}_i+ H_{>5}+\sum _{i=2}^5\varepsilon ^i\mathsf {H}_{\mathcal {R}_i}+\mathsf {H}_{\mathcal {R}_{>5}} \end{aligned}$$

with

6.32 $$\begin{aligned}&\mathsf {H}_0=\overline{\omega }\cdot \eta +\frac{1}{2}\int _{\mathbb {T}} z^2\,dx{,} \quad \mathsf {H}_1=-\frac{1}{2}\int _{\mathbb {T}} \overline{v}\,z^2\,dx{,} \quad \mathsf {H}_2=\mathbb {A}\xi \cdot \eta -\frac{1}{2}\int _{\mathbb {T}} \Psi _2(\overline{v})\,z^2\,dx{,}\nonumber \\&\mathsf {H}_i=-\frac{1}{2} \int _{\mathbb {T}} \Psi _i (\overline{v})\,{z^2}\,dx{,} \quad 3\le i\le 5{,}\;\; \mathrm{and} \;\; \Vert X_{\mathsf {H}_{>5}} \Vert ^{\gamma , \mathcal {O}_0}_s, \Vert X_{\mathsf {H}_{\mathcal {R}_{>5}}} \Vert ^{\gamma , \mathcal {O}_0}_s\nonumber \\&\lesssim _s \varepsilon ^6+\varepsilon \Vert \mathfrak {I}_{\delta } \Vert ^{\gamma , \mathcal {O}_0}_{s+\sigma } \end{aligned}$$

for some $\sigma >0$. The functions $\mathsf {H}_{\mathcal R_i}$, $\mathsf {H}_{\mathcal {R}_{>5}}$ are the quadratic forms associated to the corresponding linear operators, thus the estimates on the Hamiltonian vector fields can be deduced from (6.19), (6.18). It is easily seen that

$$\begin{aligned} \mathsf {H}_0+\sum _{i=1}^5\varepsilon ^i(\mathsf {H}_i+{\mathsf {H}_{\mathcal {R}_i}}) = \Pi ^{2d_y + d_z=2} (\mathcal {H}^{(\le 7)}\circ A_\varepsilon ) \vert _{\begin{array}{c} y=\eta \\ \theta =\varphi \end{array} }{.} \end{aligned}$$

Of course one can be even more explicit and write everything in terms of the original Hamiltonian (1.4) and of the generating functions of the weak BNF, for example one has

6.33 $$\begin{aligned}&\mathsf {H}_0 = (H^{(2)}\circ A_1)\vert _{\begin{array}{c} y=\eta \\ \theta =\varphi \end{array} }{,} \qquad \mathsf {H}_1 {\mathop {=}\limits ^{(3.22)}} (H^{(3,2)}\circ A_1)\vert _{\begin{array}{c} y=\eta \\ \theta =\varphi \end{array} }{,}\nonumber \\&\mathsf {H}_2 +\mathsf {H}_{\mathcal {R}_2}= (\Pi ^{d_y=1}(Z^{(4,0)}_2\circ A_1) + (\Pi ^{d_z=2} \{F^{(3,\le 1)}, H^{(3)}\})\circ A_1) \vert _{\begin{array}{c} y=\eta \\ \theta =\varphi \end{array} }{.} \end{aligned}$$

The terms $\mathsf {H}_i$ can be computed explicitly, however we only need to prove that they fit the following definitions.

##### Definition 6.4

We say that a matrix $\mathtt {B}:=\Big ((\mathtt {B})_{j}^{j'}(l-l')\Big )_{j, j'\in \mathbb {Z}, l, l'\in \mathbb {Z}^{\nu }}$ is *almost diagonal* if there exists a constant $\mathtt {C}>0$ such that, if $(\mathtt {B})_{j}^{j'}(l-l')\ne 0$, then $\langle j-j', l-l'\rangle \le \mathtt {C}\,$ for all $j, j'\in S^c$, $l, l'\in \mathbb {Z}^{\nu }$.

Let $B(\varphi ):H^s(\mathbb {T})\rightarrow H^s(\mathbb {T})$ be a Töpliz in time operator (recall (2.12)). We say that $B(\varphi )$ is almost diagonal if its associated matrix is almost diagonal.

Let $H:=H(\varphi )$ be a quadratic Hamiltonian of the form $H=( A(\varphi ) z, z)_{L^2}$, where $A(\varphi )$ is a Töpliz in time operator. We say that *H* and its vector field are almost diagonal if $A(\varphi )$ is almost diagonal.

##### Remark 6.5

It is easy to verify that if *X* and *Y* are almost diagonal operators then $X+Y$, $X \circ Y$ are almost diagonal.

##### Definition 6.6

Let $p\in \mathbb {N}$ and $m\in \mathbb {R}$. We say that a pseudo differential operator $\mathfrak {B}=\mathrm{Op}(b({\varphi },x,j))$ (recall Definition 2.8) is *homogenenous* of degree *p* in the function $\overline{v}$ in (6.25) if its symbol $b({\varphi },x,j)\in S^{m}$ has the form

6.34 $$\begin{aligned} b({\varphi },x,j):=\sum _{j_1,\ldots ,j_{p}\in S}C_{j_1,\ldots ,j_{p}}(j) \sqrt{\xi _{j_1}\cdots \xi _{j_p}}e^{\mathrm{i}(j_1+\ldots +j_p)x} e^{\mathrm{i}(\mathtt {l}(j_1)+\ldots +\mathtt {l}(j_p))\cdot {\varphi }}. \end{aligned}$$

##### Definition 6.7

Let $p\in \mathbb {N}$. We say that a Hamiltonian is pseudo differential and *p*-homogeneous if it has the form

6.35 $$\begin{aligned} H_p(z)=\frac{1}{2}\int _{\mathbb {T}}f_p(\overline{v})z\cdot zdx +\frac{1}{2}\int _{\mathbb {T}}\mathfrak {B}_p z\cdot z +\frac{1}{2}\int _{\mathbb {T}}\mathcal {R}_pz\cdot z{,} \end{aligned}$$

where $f_{p}$ is a homogeneous real valued function of $\overline{v}$ (of degree *p*) of the form

6.36 $$\begin{aligned} f_p(\overline{v}):=\sum _{j_1,\ldots ,j_{p}\in S}(f_p)_{j_1,\ldots ,j_{p}} \sqrt{\xi _{j_1}\cdots \xi _{j_p}}e^{\mathrm{i}(j_1+\ldots +j_p)x} e^{\mathrm{i}(\mathtt {l}(j_1)+\ldots +\mathtt {l}(j_p))\cdot {\varphi }}, \end{aligned}$$

$\mathfrak {B}_p\in OPS^{-2}$ is a p-homogeneous pseudo differential operator according to Definition 6.6 which is self-adjoint w.r.t. to $(\cdot ,\cdot )_{L^2}$; finally $\mathcal {R}_{p}$ is a *finite dimensional* operator of the form (6.16) with $g_j, \chi _j$*p*-homogeneous functions of $\overline{v}$.

##### Lemma 6.8

The Hamiltonians $\mathsf {H}_i + \mathsf {H}_{\mathcal {R}_i}$ in (6.31) are almost diagonal according to Definition 6.4 and pseudo differential homogeneous Hamiltonians according to Definition 6.7.

##### Proof

It follows by Proposition 6.2 and Remark 6.3. $\quad \square$

##### Lemma 6.9

Let $p,q\in \mathbb {N}$ and consider $H_p$, $G_q$ two pseudo differential and homogenous Hamiltonians of degree respectively *p* and *q*. Then there is a pseudo differential and $(p+q)$-homogeneous Hamiltonian $\widetilde{H}$ such that $X_{\widetilde{H}}=X_{\{H_p,G_{q}\}_{e}}$, where $\{\cdot ,\cdot \}_e$ are defined in (6.30) and $X_{H}$ denotes the Hamiltonian vector field generated by *H*.

##### Proof

By assumption $H_p$ and $G_{q}$ have the form (6.35) for some $f_{p},f_q$ real valued and some self-adjoint pseudo differential homogenenous operators $\mathfrak {B}_p$ and $\mathfrak {B}_q$. Then we have (recalling (2.2), (1.4) and (3.5))

$$\begin{aligned} \{H_p,G_q\}_{e}&=\int _{\mathbb {T}} A_1 z\cdot zdx +\int _{\mathbb {T}} A_2 z\cdot zdx, \qquad A_1:=f_{p}\circ \partial _x\circ f_{q}{,}\\ A_2&:=f_p\circ J\circ \mathfrak {B}_q+\mathfrak {B}_p\circ J\circ f_q+ \mathfrak {B}_p\circ J\circ \mathfrak {B}_q+3f_p\circ \Lambda \partial _x\circ f_q{.} \end{aligned}$$

One has that the Hamiltonian

$$\begin{aligned} \widetilde{H}:=\frac{1}{2}\int _{\mathbb {T}}(A_1+A_1^{*})z\cdot zdx +\frac{1}{2}\int _{\mathbb {T}}(A_2+A_2^{*})z\cdot zdx \end{aligned}$$

is equivalent to $\{H_p,G_q\}_e$ in the sense that they generate the same vector field. Here $A_i^{*}$, $i=1,2$, denotes the adjoint of $A_i$ w.r.t. the $L^{2}$ scalar product. Notice that

$$\begin{aligned} A_1+A_1^{*}= & {} f_{p} f_{q}\partial _x+f_p(f_q)_x-f_q\circ \partial _x\circ f_p= f_{p} f_{q}\partial _x+f_p(f_q)_x-f_qf_p\partial _x\\&-f_q(f_p)_x= f_p(f_q)_x-f_q(f_p)_x, \end{aligned}$$

which is an homogeneous function of $\overline{v}$ of degree $p+q$. Using the results on compositions of pseudo differential operators in Sect. 2 of [33], the fact that *J* is skew-self-adjoint, $\mathfrak {B}_i$, $i=p,q$, are self-adjoint, and $f_q,f_p$ are real valued, we deduce that the operator $A_2$ is a skew-self-adjoint operator in $OPS^{-1}$. Hence, using the formula (2.13) in [33] for the adjoint, we have that ${A}_2+{A}_2^*$ is pseudo differential homogeneous operator (according to Definition 6.6) in $OPS^{-2}$. $\quad \square$

## Reduction and Inversion of the Linearized Operator

The aim of the section is to prove the claim in (6.9). As explained in the introduction, first one should reduce the unbounded parts of $\mathcal {L}_{\omega }$ and then use classical KAM reducibility results to diagonalize. The difficulties arise from the fact that a few steps of this procedure must be done by hand, since they do not fit the typical smallness conditions, see [33].

The key result of this section is the following.

### Theorem 7.1

Consider $\mathcal {L}_{\omega }=\mathcal {L}_{\omega }(\mathfrak {I}_{\delta })$ in (6.15) and fix

7.1 $$\begin{aligned} \tau = 2\nu +6,\quad \mathtt {b}_0:=6\tau +6, \quad \mathtt {b}=\mathtt {b}_0+s_0. \end{aligned}$$

There exist $\mathcal S>s_0$ and $\mu _1=\mu _1(\nu )>0$ such that, if condition (6.12) is satisfied with $\mathfrak {p}_1=\mu _1$, then the following holds. There exists a constant $m(\omega )$ defined for $\omega \in \Omega _{\varepsilon }$ with

7.2 $$\begin{aligned} |m-1-\varepsilon ^{2}c(\omega ) |^{\gamma , \Omega _{\varepsilon }}\lesssim \varepsilon ^4{,} \quad |m |^{lip}\lesssim 1{,} \quad c(\omega ):=\vec {v}\cdot \xi ,\quad \vec {v}_k=\frac{2}{3} (1+\overline{\jmath }_{k}^2)\, , \;\; k=1,\ldots , \nu {,} \end{aligned}$$

^3^ such that for all $\omega$ in the set $\mathcal {O}_{\infty }^{2\gamma }$, where (recall that $\mathcal {O}_0\subseteq \mathcal {G}_0$, see (5.7))

7.3 $$\begin{aligned} \mathcal {O}_{\infty }^{2\gamma }=\mathcal {O}_{\infty }^{2\gamma }(i):= \{\omega \in \mathcal {O}_0: \; |\omega \cdot \ell - m(\omega ) j |>\frac{2{\gamma }}{\langle \ell \rangle ^{\tau }}{,} \;\forall \ell \in \mathbb {Z}^\nu , \,\,\forall j\in S^{c}\}{,} \end{aligned}$$

there exists a real, bounded linear operator $\Upsilon =\Upsilon (\omega ) : H^{s}_{S^{\perp }}\rightarrow H^{s}_{S^{\perp }}$, for all $s_0\le s\le \mathcal {S}$, such that

7.4 $$\begin{aligned} \mathcal {L}:=\Upsilon \mathcal {L}_{\omega }\Upsilon ^{-1}=\Pi _S^{\perp }\big (\omega \cdot \partial _{{\varphi }}-m J-\varepsilon ^2 \mathfrak {D}(\omega ) +\mathcal {P}_0 \big ) \end{aligned}$$

where $\mathfrak {D}(\omega )$ is the diagonal operator of order $-1$ defined as $\mathfrak {D}:=\mathfrak {D}(\omega )= \mathrm {diag}(\mathrm {i}\kappa _j)_{j\in S^c}$, with

7.5 $$\begin{aligned} \kappa _{j}:=\kappa _j(\omega ):= \lambda ( j)\big (\mathfrak {l}_j(\omega )-c(\omega )\big )\in \mathbb {R}{,} \quad |\kappa _{j}|^{ sup}\lesssim |j|^{-1}{,} \end{aligned}$$

where $\mathfrak {l}_{j}$ is defined in (5.6). The constant *m* depends on *i* and for $\omega \in \mathcal {O}_{\infty }^{2\gamma }(i_1)\cap \mathcal {O}_{\infty }^{2\gamma }(i_2)$ one has

7.6 $$\begin{aligned} |\Delta _{12}m |\lesssim \varepsilon \Vert i_1-i_2 \Vert _{s_0+\mu _1}{,} \end{aligned}$$

where $\Delta _{12}m:=m(i_1)-m(i_2)$. The remainder $\mathcal {P}_0$ in (7.4) is defined and Lipschitz in $\omega$ belonging to the set $\mathcal {O}^{2\gamma }_\infty$ and is Lip-$-1$-modulo tame (see Definition 2.7) with

7.7 $$\begin{aligned}&\mathfrak {M}^{\sharp , \gamma ^{3/2}}_{\mathcal {P}_0}(-1,s,\mathtt {b}_0) \lesssim _s \varepsilon ^{4-3 a} +\varepsilon \gamma ^{-1}\Vert \mathfrak {I}_{\delta }\Vert _{s+{\mu _1}}^{\gamma , \mathcal {O}_0}{,} \end{aligned}$$

7.8 $$\begin{aligned}&\Vert {\langle D_x\rangle }^{1/2}\underline{\Delta _{12}\mathcal {P}_0 }{\langle D_x\rangle }^{1/2}\Vert _{\mathcal L(H^{s_0})}, \,\, \Vert {\langle D_x\rangle }^{1/2}\underline{\Delta _{12} \langle \partial _{{\varphi }}\rangle ^{{\mathtt {b}_0}} \mathcal {P}_0 }{\langle D_x\rangle }^{1/2}\Vert _{\mathcal L(H^{s_0})}\nonumber \\&\quad \lesssim \varepsilon {\gamma }^{-1} \Vert i_1-i_2\Vert _{s_0+\mu _1}{,} \end{aligned}$$

for all $\omega \in \mathcal {O}_{\infty }^{2{\gamma }}(i_1)\cap \mathcal {O}_{\infty }^{2{\gamma }}(i_2)$. Moreover if $u=u(\omega )$ depends on the parameter $\omega \in \mathcal {O}_{\infty }^{2 \gamma }$ in a Lipschitz way then

7.9 $$\begin{aligned} \Vert \Upsilon ^{\pm 1} u \Vert _s^{{\gamma }, \mathcal {O}_{\infty }^{2 \gamma }} \lesssim _s \Vert u \Vert _s^{{\gamma },\mathcal {O}_{\infty }^{2\gamma }} +\varepsilon \gamma ^{-1} \Vert \mathfrak {I}_{\delta } \Vert ^{{\gamma }, \mathcal {O}_0}_{s+\mu _1} \Vert u \Vert ^{\gamma , \mathcal {O}_{\infty }^{2\gamma }}_{s_0}{,} \;\;\; s_0\le s\le \mathcal {S}{.} \end{aligned}$$

The result above has two relevant consequences. Firstly it shows that the operator $\mathcal {L}_{\omega }$ in (6.15) can be conjugated to an operator (see (7.4)) which is “diagonal”, at the highest order of derivatives, plus a remainder which is $-1$-smoothing. In addition to this, thanks to a *linear BNF* procedure (performed in Sect. 7.2), the non-diagonal term $\mathcal {P}_0$ in (7.4) has a size much smaller than $\varepsilon$ (see estimates (7.7), (7.8)). In particular it is “perturbative” w.r.t. the constant $\gamma$ in (5.3). This allows us to apply the reducibility scheme of [33] in order to complete the diagonalization of the operator $\mathcal {L}$ (see Theorem 7.13). Then the inversion assumption (6.9) follows directly from Proposition 7.14).

*Strategy of the Proof of Theorem*7.1.

- **Reduction at the highest order** The first step is to exploit the pseudo differential structure of the operator $\mathcal {L}_{\omega }$ in order to conjugate it to an operator which has constant coefficients up to a smoothing remainder of order $-1$. To this purpose we use changes of variables generated as the time-one flow map $\Phi ^{\tau }\vert _{\tau =1}$ of Hamiltonians of the form
  7.10 $$\begin{aligned} S(\tau ,{\varphi },z)= & {} \int b(\tau ,{\varphi },x) z^2 dx{,} \qquad b(\tau ,{\varphi },x):=\frac{\beta ({\varphi },x)}{1+\tau \beta _{x}({\varphi },x)}, \end{aligned}$$
  7.11 $$\begin{aligned} \partial _{\tau } \Phi ^{\tau } u= & {} \Pi _S^{\perp }[(J\circ b) \Pi _S^{\perp }[\Phi ^{\tau }u]]{,}\qquad \Phi ^0 u=u{,} \end{aligned}$$
  where $\beta$ is some smooth function. In Proposition C.2 we show that $\Phi ^{\tau }$ is well defined as symplectic map on $H^{s}_{S^{\perp }}$ (see Lemma C.1) and study the structure of $\Phi ^{\tau }\mathcal {L}_\omega (\Phi ^{\tau })^{-1}$. Proposition C.2 gives an explicit formula for the new coefficient at the highest order (see (C.17)). Then Corollary 3.6 of [31] (see also Proposition 3.6 in [33]) provides the solution for the Eq. (C.17)=const provided that some smallness condition is satisfied. This smallness condition has the form
  7.12 $$\begin{aligned} C(s_1)\gamma ^{-1}\Vert a_0\Vert _{s_1}^{\gamma ,\mathcal {O}}\ll 1 \end{aligned}$$

for some $s_0+\mathfrak {p}_1>s_1>s_0$ and some constant $C(s_1)>0$. As shown in [33], due to the Hamiltonian structure, this reduces $\mathcal {L}_{\omega }$ to constant coefficients up to a correction of order $-1$.

Unfortunately, since here $\gamma = \varepsilon ^{2+a}$, $a>0$, by (6.17), the coefficient $a_0({\varphi },x)$ in $\mathcal {L}_{\omega }$ does not satisfy (7.12). This is why we have to perform some preliminary steps in order to enter in the perturbative regime where we apply the scheme described in the proof of Corollary 3.6 in [31].

We first “regularize” the purely polynomial terms $\mathsf {H}_i$ (see (6.32)) by hand, by exploiting their homogeneity according to Definition 6.7. After that we are left with only unbounded terms which satisfy the smallness conditions of [33]. We “regularize” them by applying the results of [33] adapted to our slightly more general setting, see Proposition C.2.

### Remark 7.2

In order to determine the correct change of variables in the regularization of $\mathsf {H}_i$, it will be convenient to use the *Formal Lie expansions*. We recall that $H\circ (\Phi ^{\tau })^{-1}$ satisfies, for $\tau \in [0,1]$,

7.13 $$\begin{aligned} \partial _{\tau }(H\circ (\Phi ^{\tau })^{-1}) =\{S(\tau ),H\circ (\Phi ^{\tau })^{-1}\}. \end{aligned}$$

By setting $S:=S(0)$, the Lie expansion of the conjugated Hamiltonian $H\circ \Phi ^{-1}$ is the following:

7.14 $$\begin{aligned} H\circ (\Phi ^\tau )^{-1}=H+\tau \{S,H\}_{e} +\frac{\tau ^2}{2}\Big (\{S,\{S,H\}_{e}\}_{e} +\{(\partial _{\tau }S)(0),H\}_{e}\Big )+\cdots {,} \end{aligned}$$

where the Poisson brackets $$\{\cdot ,\cdot \}_{e}$$ are in (6.30). Recall that $$\Phi $$ is a $$C^k$$ map from $$H^s $$ to $$H^{s-k}$$. Therefore the Taylor expansion of the conjugated Hamiltonian coincides with the Lie series of the generator up to any order $$\tau ^k$$.

- **Linear BNF** The second step is to diagonalize the bounded terms. Here we diagonalize “by hand” the terms up to order $\varepsilon ^3$, by exploiting the fact that they are almost diagonal according to Definition 6.4 and applying a linear BNF. Once this is done, the full diagonalization follows by a standard KAM reducibility theorem (see Theorem 7.13).

### Reduction at the highest order

In the following we shall assume that the (6.12) holds with some $\mathfrak {p}_1\gg 1$. The loss of regularity $\mathfrak {p}_1$ will be determined explicitly at the end of the section. In order to perform the *non-perturbative* steps, we construct changes of coordinates ${\mathcal {B}}_{i}$, $i=1,2,3,4,5$, as the time-one flow maps generated by Hamiltonians as in (7.10). Then we set $\mathcal L_0:= \mathcal L_{\omega }$ and define iteratively $\mathcal {L}_i:={\mathcal {B}}_i \mathcal {L}_{i-1} {\mathcal {B}}_i^{-1}$. Note that $\mathcal L_0$ is pseudo differential plus a finite rank operator. Even though the ${\mathcal {B}}_{i}$ preserve the pseudo differential structure, in order to have a good quantitative control on the symbols we shall fix appropriate values

7.15 $$\begin{aligned} p \ge s_0{,}\quad \rho \ge s_0+6\tau +9{,} \end{aligned}$$

and write

7.16 $$\begin{aligned} \mathcal {L}_i={\mathcal {B}}_i \mathcal {L}_{i-1} {\mathcal {B}}_i^{-1}=\Pi _S^{\perp } \Big ( \omega \cdot \partial _{\varphi }-J\circ \big (1+\varepsilon ^2 c_i(\omega )+a_i(\varphi , x) \big )+\mathrm{Op}(\mathtt {q}_i)+\widehat{\mathcal {Q}}_i\Big ) \end{aligned}$$

where $c_i(\omega )$ is a constant, $a_i,\mathtt {q}_i$ are symbols, $\mathrm{Op}(\mathtt {q}_i)$ is of order $-1$ and $\widehat{\mathcal {Q}}_i\in \mathfrak {L}_{\rho ,p}$. This is a class of operators of order $-\rho$ which we introduced in [33] (we recall it in Definition C.5). Note that by Lemma C.7$\mathcal {Q}_0=:\widehat{\mathcal {Q}}_0$ belongs to $\mathcal {L}_{\rho , p}$ for all $\rho ,p$, with bounds on $\mathbb {M}^{\gamma }_{\widehat{\mathcal {Q}}_0}(s, \mathtt {b})$ given in the same lemma. Then one proves iteratively that

7.17 $$\begin{aligned} \Vert a_i \Vert _s^{\gamma , \mathcal {O}_0}&\lesssim _s \varepsilon ^{i+1} +\varepsilon \Vert \mathfrak {I}_{\delta }\Vert _{s+\sigma _0+\sigma _{i+3}}^{\gamma , \mathcal {O}_0}{,}\; s\ge s_0, \nonumber \\ \Vert \Delta _{12} a_i \Vert _{p}&\lesssim _{p} \varepsilon (1+\Vert \mathfrak {I}_{\delta } \Vert _{p+\sigma _0+\sigma _{i+3}})\Vert i_1-i_2\Vert _{p+\sigma _0+\sigma _{i+3}}. \end{aligned}$$

7.18 $$\begin{aligned} |\mathtt {q}_i |^{\gamma , \mathcal {O}_0}_{-1, s, \alpha }&\lesssim _{s, \alpha , \rho } \varepsilon (1+\Vert \mathfrak {I}_{\delta }\Vert ^{\gamma , \mathcal {O}_0}_{s+\sigma _0+\sigma _{i+3}}), \quad s\ge s_0{,}\nonumber \\ |\Delta _{12} \mathtt {q}_i |_{-1, p, \alpha }&\lesssim _{p, \alpha , \rho } \varepsilon (1+\Vert \mathfrak {I}_{\delta } \Vert _{p+\sigma _0+\sigma _{i+3}})\Vert i_1-i_2\Vert _{p+\sigma _0+\sigma _{i+3}}. \end{aligned}$$

Note that the size of $a_{i}$ (in the low norm) is decreasing in *i*. Regarding the remainders, the numbers $\mathbb {M}^{\gamma }_{\widehat{\mathcal {Q}}_i}(s, \mathtt {b})$ control the norm of the corresponding operator, see Definition C.5. We have

7.19 $$\begin{aligned} \mathbb {M}^{\gamma }_{\widehat{\mathcal {Q}}_i}(s, \mathtt {b})&\lesssim _{s, \rho } \varepsilon (1+\Vert \mathfrak {I}_{\delta }\Vert ^{\gamma , \mathcal {O}_0}_{s+\sigma _0+\sigma _{i+3}}), \quad s_0\le s\le \mathcal {S}\, ,\qquad 0\le \mathtt {b}\le \rho -2, \nonumber \\ {\mathbb {M}_{\Delta _{12} \widehat{\mathcal {Q}}_i }(p, \mathtt {b}) }&\lesssim _{p, \rho } \varepsilon (1+\Vert \mathfrak {I}_{\delta } \Vert _{p+\sigma _0+\sigma _{i+3}})\Vert i_1-i_2\Vert _{p+\sigma _0+\sigma _{i+3}},\qquad 0\le \mathtt {b}\le \rho -3, \end{aligned}$$

with $$\sigma _0$$ defined in Proposition 6.2 and $$\sigma _{i+3}>0$$$$i=1,\ldots ,5$$, depending only on $$\nu $$ (essentially $$\sigma _{i+3}$$ are the losses coming from the application of Proposition C.2). Note that we can obtain (7.16) for any $$\rho ,p$$ satisfying (7.15); however, if we want (7.18) to hold for some given *p*, we have to assume a smallness condition (6.12) with $$p+\sigma _0+\sigma _{i+3}<\mathfrak {p}_1$$.

**Step** ($\varepsilon$). Consider the Hamiltonian

7.20 $$\begin{aligned} S(\tau )&:=\frac{1}{2} \int _{\mathbb {T}} b_1(\tau ,\varphi , x)\,z^2\,dx =\varepsilon S_1+\varepsilon ^2\tau S_2+\varepsilon ^3\tau ^{2} S_3+S_4(\tau ), \quad b_1:=\frac{\varepsilon \beta _1}{1+\tau \varepsilon (\beta _1)_x}, \end{aligned}$$

7.21 $$\begin{aligned} S_1&:=\frac{1}{2} \int _{\mathbb {T}} \beta _1\,z^2\,dx, \quad S_2:=-\frac{1}{4}\int _{\mathbb {T}}\partial _x (\beta _1^2)\,z^2\,dx, \quad S_3:=\frac{1}{2} \int _{\mathbb {T}}\beta _1 (\beta _1)_x^2z^{2}\,dx, \end{aligned}$$

with $S_4(\tau )\sim O(\tau ^3\varepsilon ^{4})$ and for some function $\beta _1$ of the form (6.36) with $p=1$ and some coefficients $(\beta _1)_{j}$, $j\in S$, to be determined. The Hamiltonian system associated to $S(\tau )$ is of the form (7.11) with $b\rightsquigarrow b_1$. We call ${\mathcal {B}}_1$ the flow at time-one generated by $S(\tau )$, then the Hamiltonian of the conjugated linearized operator ${\mathcal {B}}_1 \mathcal {L}_{\omega } {\mathcal {B}}_1^{-1}$ is (recall (6.31), (6.32) and Remark 7.2)

7.22 $$\begin{aligned} \mathsf {H}\circ {\mathcal {B}}_1^{-1} \,&= \mathsf {H}_0+\varepsilon \mathsf {H}^{(1)}_1+\varepsilon ^{2}\mathsf {H}^{(1)}_2+\varepsilon ^{3}\mathsf {H}_3^{(1)} +o(\varepsilon ^{3}){,} \qquad \mathsf {H}_1^{(1)}:= \{ S_1, \mathsf {H}_0\}_{e}+\mathsf {H}_1{,} \end{aligned}$$

7.23 $$\begin{aligned} \mathsf {H}_2^{(1)}&:=\frac{1}{2}\{ S_1,\{ S_1, \mathsf {H}_0\}_e\}_{e} + \{S_1,\mathsf {H}_1\}_{e}+\frac{1}{2}\{S_2,\mathsf {H}_0\}_{e} +\mathsf {H}_2+\mathsf {H}_{\mathcal {R}_2}{,} \end{aligned}$$

where, by Lemma 6.9, $\mathsf {H}_3^{(1)}$ is some pseudo differential 3-homogeneous Hamiltonian of the form (6.35). Notice that also $H_1^{(1)}$ and $\mathsf {H}_2^{(1)}$ (for $\eta =0$) are pseudo differential and 1-homogeneous, resp. 2-homogenenous, Hamiltonians according to Definition 6.7. We want to solve the following equation

7.24 $$\begin{aligned} \mathsf {H}_1^{(1)}=\mathsf {H}_1+\{ S_1, \mathsf {H}_0\}_{e}=\mathsf {H}_1+\{ S_1, \mathsf {H}_0\}+\overline{\omega }\cdot \partial _{\varphi } S_1= \int \mathfrak {B}_1( z)\,z\,dx{,} \end{aligned}$$

where $\mathfrak {B}_1$ is some pseudo differential operator of order $-2$. Recalling (6.32), and expanding $\{S_1,\mathsf {H}_0\}$ as in the proof of Lemma 6.9, we note that the Eq. (7.24) is equivalent to the following one

7.25 $$\begin{aligned} \overline{\omega }\cdot \partial _{\varphi } \beta _1-(\beta _1)_x-\overline{v}=0. \end{aligned}$$

Hence we choose $\beta _1=\frac{1}{3} (\Lambda \partial _x)^{-1} \overline{v}$ and we note that

7.26 $$\begin{aligned} \Vert \beta _1 \Vert ^{\gamma , \mathcal {O}_0}_s \lesssim _s 1{,} \quad \forall s\ge s_0{.} \end{aligned}$$

With the choice in (7.25) we have

7.27 $$\begin{aligned} \mathfrak {B}_1:=[3 \Lambda \partial _x, \beta _1]. \end{aligned}$$

In this way the Hamiltonians in (7.22), (7.23) become

7.28 $$\begin{aligned} \mathsf {H}^{(1)}_1:= & {} \int \mathfrak {B}_1( z)\,z\,dx{,}\qquad \mathsf {H}^{(1)}_2:=\mathsf {H}_2+\frac{1}{2}\{S_{2},\mathsf {H}_0\}_{e} +\mathsf {H}_{\mathcal {R}_2} +\frac{1}{2}\{ S_1, \mathsf {H}_1\}\nonumber \\&+\frac{1}{2}\{S_1, \int _{\mathbb {T}} \mathfrak {B}_1(z)\,z\,dx\} {.} \end{aligned}$$

By (7.26) the smallness assumption of Lemma C.1 is satisfied. By (6.17), (6.21), (6.22) and using the assumption (6.12) with $\mathfrak {p}_1$ sufficiently large the condition (C.15) holds. In this case $\mathtt {q}\rightsquigarrow 0$ (see (C.13)).

Then Proposition C.2 applies and the new linearized operator $\mathcal {L}_1:={\mathcal {B}}_1 \mathcal {L}_{\omega } {\mathcal {B}}_1^{-1}$ has the form in (7.16) with $i=1$ and $c_1=0$. By (7.26), (6.21), (C.19), (C.17), (6.15), we have that $\widehat{\mathcal {Q}}_1\in \mathfrak {L}_{\rho , p}(\mathcal {O})$ (see Definition C.5) (with $\rho$, *p* satisfying (7.15)) and (7.18), (7.19) hold for $i=1$.

The only estimates that are not given by Lemma C.2 are (7.17). The coefficient $a_1$ is given by (C.17) with $m\rightsquigarrow 1$, $a\rightsquigarrow a_0$, $a_+\rightsquigarrow a_1$ and $\tilde{\beta }$ such that $x\mapsto x+\tilde{\beta }$ is the inverse of $x\mapsto x+\beta _1$. By the choice of $\beta _1$ in (7.25) we have eliminated the $\varepsilon$-terms from $a_1$. Hence by (7.26) and (6.17) we get (7.17) for $i=1$.

**Step** ($\varepsilon ^2$). Now we deal with the terms of order $\varepsilon ^2$ of the Hamiltonian (6.31). We consider the auxiliary Hamiltonian

7.29 $$\begin{aligned}&\tilde{S}(\tau )=\frac{1}{2} \int _{\mathbb {T}} b_2( x,\varphi )\,z^2\,dx=\varepsilon ^2\tilde{S}_2+\tilde{S}_4(\tau ), \quad b_2 :=\frac{\varepsilon ^2\beta _2}{1+\tau \varepsilon ^2 (\beta _2)_x}, \nonumber \\&\quad \tilde{S}_2:=\frac{1}{2} \int _{\mathbb {T}} \beta _2\,z^2\,dx, \end{aligned}$$

where $\tilde{S}_4(\tau ):=\tilde{S}(\tau )-\varepsilon ^2\tilde{S}_2 \sim O(\tau \varepsilon ^{4})$ and $\beta _2$ is some function of the form (6.34), with $p=2$, to be determined. Notice that $(\partial _{\tau }\tilde{S})(0)\sim O(\varepsilon ^{4})$. The Hamiltonian system associated to $\tilde{S}(\tau )$ is of the form (7.11) with $b\rightsquigarrow b_2$. If ${\mathcal {B}}_2$ is the flow at time-one generated by $\tilde{S}(\tau )$, then the Hamiltonian of the conjugated linearized operator $\mathcal {L}_2:={\mathcal {B}}_2\mathcal {L}_1 {\mathcal {B}}_2^{-1}$ is (recall (7.22), (7.28), (7.14))

7.30 $$\begin{aligned}&\mathsf {H}\circ {\mathcal {B}}_1^{-1}\circ {\mathcal {B}}_2^{-1}= \mathsf {H}_0+\varepsilon \mathsf {H}^{(1)}_1+\varepsilon ^{2}\mathsf {H}^{(2)}_2 +\varepsilon ^{3}\mathsf {H}^{(2)}_3+o(\varepsilon ^{3})\nonumber \\&\mathsf {H}^{(2)}_2:=\mathsf {H}^{(1)}_2+\{ \tilde{S}_2, \mathsf {H}_0\}_{e}{,} \qquad \mathsf {H}^{(2)}_3:=\mathsf {H}_3^{(1)}+\{\tilde{S}_2,\mathsf {H}_1^{(1)}\}_{e}{.} \end{aligned}$$

We want to solve the equation

7.31 $$\begin{aligned} \mathsf {H}^{(2)}_2=\mathsf {H}^{(1)}_2 +\overline{\omega }\cdot \partial _{\varphi } \tilde{S}_2+\{\tilde{S}_2 ,\mathsf {H}_0\} = c+\int _{\mathbb {T}} \mathfrak {B}_2( z)\,z\,dx\,+\mathsf {H}_{\mathcal {R}_2}, \end{aligned}$$

where $\mathfrak {B}_2$ is some pseudo differential and 2-homogeneous operator of order $-2$ (see Definition 6.6), *c* is some constant to be determined and $\mathsf {H}_{\mathcal {R}_2}$ (possibly different from the one in (6.31)) is a Hamiltonian with the form (6.35). By Lemma 6.9 we have that $\mathsf {H}^{(1)}_2 +\{\tilde{S}_2 ,\mathsf {H}_0\}$ can be written in the form (6.35) with, in particular (see (6.24) for the definition of $\Psi _2$)

7.32 $$\begin{aligned} f_2(\overline{v}):=-\Psi _2(\overline{v}) +\frac{1}{4}\partial _{xx}(\beta _1^2)-\frac{1}{2}\beta _1 \overline{v}_x+\frac{1}{2}\overline{v} (\beta _1)_x, \end{aligned}$$

and some $\mathfrak {B}_2\in OPS^{-2}$, as in Definition 6.6, up to a finite rank remainder. Hence the Eq. (7.31) is equivalent to

7.33 $$\begin{aligned} \overline{\omega }\cdot \partial _{\varphi } \beta _2 - (\beta _2)_x +f_2(\overline{v})=c{.} \end{aligned}$$

Since $f_2$ in (7.32) has the form (6.36) with $p=2$, we look for a function $\beta _2$ of the same form in (6.36) with some coefficients $(\beta _2)_{j_1,j_2}\in \mathbb {C}$. Hence Eq. (7.33) reads

7.34 $$\begin{aligned}&\big [{\lambda }(j_1)\!+\!{\lambda }(j_2)-(j_1+j_2)\big ](\beta _2)_{j_1,j_2}+(f_2)_{j_1,j_2}\!=\!0, \quad \mathrm{for}\quad {\lambda }(j_1)+{\lambda }(j_2)\!-\!(j_1+j_2)\ne 0{,}\nonumber \\&(f_2)_{j_1,j_2}\!=\!c,\quad \mathrm{for}\quad {\lambda }(j_1)\!+\!{\lambda }(j_2)-(j_1+j_2)=0{.} \end{aligned}$$

We have that, for $j_1, j_2\in S$, ${\lambda }(j_1)+{\lambda }(j_2)-(j_1+j_2)=0$ if and only if $j_1+j_2=0$, since $j_1j_2\ne -1$. The terms with $j_1=-j_2$ corresponds to the average in *x* of the function $f_2(\overline{v})$. Hence we set

7.35 $$\begin{aligned} c:=\frac{1}{2\pi }\int _{\mathbb {T}}f_{2}(\overline{v})dx. \end{aligned}$$

and we evaluate explicitly it. The functions $\Psi _2(\overline{v})$ and $\partial _{xx}(\beta _1^2)$ do not contribute since they have zero average in space.

Recalling that $\beta _1=\frac{1}{3} (\Lambda \partial _x)^{-1} \overline{v}$ we have

$$\begin{aligned} \int _{\mathbb {T}}f_{2}(\overline{v})dx&= \frac{1}{6}\int _{\mathbb {T}}\Big ((\Lambda ^{-1}\partial _{x}^{-1}\partial _x\overline{v})\cdot \overline{v} -(\Lambda ^{-1}\partial _x^{-1}\overline{v})\cdot \overline{v}_x\Big ) dx=\frac{1}{3}\int _{\mathbb {T}}(\Lambda ^{-1}\overline{v})\cdot \overline{v}dx\\&{\mathop {=}\limits ^{(3.5)}}\frac{1}{3}\int _{\mathbb {T}}(\overline{v}^2+\overline{v}^{2}_x){.} \end{aligned}$$

Then the constant $c=c(\omega )$ (recall the (4.11)) in (7.35) is given by

7.36 $$\begin{aligned} c(\omega )=\frac{1}{3} \sum _{j\in S} (1+j^2)\,\xi _j=\frac{2}{3} \sum _{j\in S^+}(1+j^2)\,\xi _j. \end{aligned}$$

By noting that

7.37 $$\begin{aligned} \Vert \varepsilon ^2\beta _2 \Vert ^{\gamma , \mathcal {O}}_s\lesssim _s \varepsilon ^2 \quad \forall s\ge s_0, \end{aligned}$$

by (7.18)–(7.17) with $i=1$ and using the assumption (6.12) with $\mathfrak {p}_1$ sufficiently large the smallness assumption of Lemma C.1 and the condition (C.15) are satisfied. In this case $\mathtt {q}\rightsquigarrow \mathtt {q}_1$, hence by (7.18), (7.19) the bounds (C.13), (C.14) hold with $\mathtt {k}_1\rightsquigarrow \varepsilon$, $\mathtt {k}_2\rightsquigarrow \varepsilon$, $\mathtt {k}_3\rightsquigarrow \varepsilon$, $f\rightsquigarrow \mathfrak {I}_{\delta }$. Then Proposition C.2 applies and $\mathcal {L}_2:=\Phi _2 \mathcal {L}_1 \Phi _2^{-1}$ with $i=2$ and $c_2(\omega ):=c(\omega )$ given in (7.36). By (7.37), (6.21), (C.19), (C.17) we have that $\widehat{\mathcal {Q}}_2\in \mathfrak {L}_{\rho , p}(\mathcal {O})$ (with $\rho$, *p* as in (7.15)) and (7.18), (7.19) hold for $i=2$. By (7.37), (7.18)–(7.17) for $i=1$, (C.20), we have that (7.18)–(7.17) holds for $i=2$.

**Steps**$(\varepsilon ^{3})$-$(\varepsilon ^4)$-$(\varepsilon ^5)$. Consider $i=3,4, 5$. We proceed exactly as in the previous steps. We consider a change of coordinates ${\mathcal {B}}_i$ as the time-one flow map of

7.38 $$\begin{aligned} u_{\tau }=\Pi _S^{\perp }[\big (J\circ b_i(\tau )\big )\,u], \quad b_i:=\frac{\varepsilon ^i\beta _i}{1+\varepsilon ^i\tau (\beta _i)_x} \end{aligned}$$

for some smooth function $\beta _i$ of the form (6.36) (with $p=i$) to be determined. Using Lemma 6.9 for the Hamiltonians of order $\varepsilon ^{i}$, $i=3,4,5$, we can choose $\beta _i$ in order to solve an equation like the following

7.39 $$\begin{aligned} \overline{\omega }\cdot \partial _{\varphi } \beta _i-(\beta _i)_x=f_i(\overline{v}), \end{aligned}$$

where $f_i$ is a homogeneous function as in (6.36) (with $p=i$). The condition (1.13) implies that the Eq. (7.39) for $i=4$ is solved up to remainders of the form

7.40 $$\begin{aligned} d(\omega ):=d(\xi (\omega ))=\sum _{j_1, j_2\in S} \mathtt {d}(j_1, j_2)\xi _{j_1}\xi _{j_2}{.} \end{aligned}$$

By (A.3) there are no small divisors for (7.39) if $i=3$ or $i=5$. By (7.18), (7.19) and by noting that

7.41 $$\begin{aligned} \Vert \varepsilon ^i\beta _i \Vert ^{\gamma , \mathcal {O}}_s \lesssim _s \varepsilon ^i \quad i=3,4{,} 5{,}\,\, \forall s\ge s_0{,} \end{aligned}$$

the smallness assumption of Lemma C.1 and the condition (C.15) are satisfied for the system (7.38). Arguing as in the previous steps we obtain that $\mathcal {L}_5:={\mathcal {B}}_5{\mathcal {B}}_4 \mathcal {L}_3 {\mathcal {B}}_4^{-1}{\mathcal {B}}_5^{-1}$ has the form (7.16) with $i=5$ and $c_5=c_2+\varepsilon ^2 d(\xi )$. Moreover the bounds (7.18)–(7.17) hold for $i=5$.

#### Remark 7.3

Since the symplectic maps $\mathcal {B}_i$, $i=1, \ldots , 5$ are smooth in $\varepsilon \,\overline{v}$ (see (6.25) and Remark 7.2) and the Hamiltonian $\mathsf {H}$ has the Taylor expansion (6.31), then the operators $\mathrm{Op}(\mathtt {q}_5), \widehat{\mathcal {Q}_5}$ in (7.16) may be expanded, in degree of homogeneity of $\varepsilon \overline{v}$ , in the following way (see Remark C.3)

$$\begin{aligned} \mathrm{Op}(\mathtt {q}_5)=\sum _{i=1}^3 \varepsilon ^i \,\mathtt {q}_5^{(i)}+\mathtt {q}_5^{(\ge 4)}, \quad \widehat{\mathcal {Q}}_5:=\sum _{i=1}^3 \varepsilon ^i \widehat{\mathcal {Q}}^{(i)}_5+\widehat{\mathcal {Q}}^{(\ge 4)}_5 \end{aligned}$$

with

7.42 $$\begin{aligned} |\mathtt {q}_5^{(\ge 4)}|^{\gamma , \mathcal {O}_0}_{-1, s, \alpha }\lesssim _{s, \alpha , \rho } \varepsilon ^4+\varepsilon \Vert \mathfrak {I}_{\delta } \Vert ^{\gamma , \mathcal {O}_0}_{s+\sigma }, \quad \mathbb {M}^{\gamma }_{\widehat{\mathcal {Q}}^{(\ge 4)}_5}(s)\lesssim _{s, \rho } \varepsilon ^4+\varepsilon \Vert \mathfrak {I}_{\delta } \Vert ^{\gamma , \mathcal {O}_0}_{s+\sigma } \end{aligned}$$

for some $\sigma >0$. Following Remark C.3 the $\widehat{\mathcal {Q}}^{(i)}_5, \widehat{\mathcal {Q}}^{(\ge 4)}_5$ are in $\mathfrak {L}_{\rho ', p'}$, as is habitual we rename them $\rho ,p$.

By (6.31) and the fact that the generators $\beta _i$ in (7.10) are $\mathfrak {I}_{\delta }$-independent, it is clear that $\mathtt {q}_5^{(\ge 4)}$ and $\widehat{\mathcal {Q}}^{(\ge 4)}_5$ contain terms of size $\varepsilon ^4$, which are functions just of $\overline{v}$, and terms dependent also on $\mathfrak {I}_{\delta }$ of "size" $O(\varepsilon \Vert \mathfrak {I}_{\delta } \Vert _{s+\sigma } )$, see the estimates (7.17), (7.18), (7.19). By the uniqueness of the Taylor expansion we have that $\sum _{i=1}^3 \varepsilon ^i \,\big ( \,\mathtt {q}_5^{(i)}+\widehat{\mathcal {Q}}^{(i)}_5\big )$ coincide with the vector field $-\sum _{i=1}^3 \varepsilon ^i\,J\,\nabla \,\mathcal {K}_i$ where, recalling (7.22), (7.30), (7.23), (7.36),

7.43 $$\begin{aligned} \mathcal {K}_1:=\mathsf {H}_1^{(1)},\qquad \mathtt {Z}_0+ \mathcal {K}_2:=\mathsf {H}_2^{(2)} {,}\qquad \mathtt {Z}_0:=\mathbb {A} \xi \cdot \eta +\frac{c(\omega )}{2}\int _{\mathbb {T}} z^2\,dx{,} \end{aligned}$$

and $\mathcal {K}_3$ is some pseudo differential 3-homogeneous Hamiltonian as in (6.35) with the corresponding function $f_{3}(\overline{v})=0$.

Now we apply Proposition 3.6 in [33] (or Corollary 3.6 in [31]) in order to make constant the coefficient $a_5$ of the linearized operator $\mathcal {L}_5$, namely we find $\beta$ such that

7.44 $$\begin{aligned} \omega \cdot \partial _{\varphi } \beta -(1+\varepsilon ^2 c(\omega )+\varepsilon ^4 d(\omega )+a_5(\varphi , x))(1+\beta _x)=\text{ constant }. \end{aligned}$$

Note that, by (7.17) with $i=5$ and (6.12), the smallness condition (7.12) is satisfied by the function $a_{5}$. We have the following.

#### Proposition 7.4

There exists $\beta ^{(\infty )}(\varphi , x)$ such that $(\varphi , x)\mapsto (\varphi , x+\beta ^{(\infty )}(\varphi , x))$ is a diffeomorphism of the torus $\mathbb {T}^{\nu +1}$ with the following estimates (recall (7.3)),

7.45 $$\begin{aligned}&\Vert \beta ^{(\infty )}\Vert _s^{{\gamma }, \mathcal {O}_{\infty }^{2\gamma }} \lesssim _s {\gamma }^{-1}\Vert a_5 \Vert _{s+2\tau +4}^{{\gamma }, \mathcal {O}_{0}}{,} \quad \forall s\ge s_0{,} \quad \nonumber \\&\quad \Vert \Delta _{12} \beta ^{(\infty )} \Vert _{p} \lesssim _{p} \varepsilon \gamma ^{-1} (1+\Vert \mathfrak {I}_{\delta } \Vert _{p+\widehat{\sigma }})\Vert i_1-i_2\Vert _{p+\widehat{\sigma }}{,} \end{aligned}$$

for some $\widehat{\sigma }>0$, and the following holds. If $\beta$ is the function such that $(\varphi , x)\mapsto (\varphi , x+\beta (\varphi , x))$ is the inverse of the above diffeomorphism and ${\mathcal {B}}_6$ is the flow of the Hamiltonian PDE

7.46 $$\begin{aligned} u_{\tau }=\Pi _S^{\perp }[\big (J \circ b(\tau )\big )\,u)]{,} \qquad b(\tau ):=b(\tau , \varphi , x)=\frac{\beta }{1+\tau \beta _x}{,} \end{aligned}$$

then the conjugated of the operator $\mathcal {L}_5$ in (7.16) with $i=5$ is

7.47 $$\begin{aligned} \mathcal {L}_6:={\mathcal {B}}_{6}\,\mathcal {L}_5\,{\mathcal {B}}^{-1}_{6} = \Pi _S^{\perp }\Big (\omega \cdot \partial _{\varphi }-m J+\mathcal {Q}_6 \Big ){,} \end{aligned}$$

where $\mathcal {Q}_6=\mathrm{Op}(\mathtt {q}_6)+\widehat{\mathcal {Q}}_6$ is of order $-1$, as in Proposition C.2, and *m* is a constant such that

7.48 $$\begin{aligned}&|m-1-\varepsilon ^2 c(\omega )-\varepsilon ^4 d(\omega ) |^{\gamma }\lesssim \varepsilon ^{10}{\gamma }^{-2}{,} \quad |m|^{lip}\lesssim 1{,} \qquad \nonumber \\&\quad |\Delta _{12} m |\lesssim \varepsilon \Vert i_1-i_2 \Vert _{s_0+2}{,} \quad \forall \omega \in \mathcal {O}^{2\gamma }_{\infty }{.} \end{aligned}$$

Moreover, for any $s\ge s_0$,

7.49 $$\begin{aligned} |\mathtt {q}_6 |^{\gamma , \mathcal {O}^{2\gamma }_{\infty }}_{-1, s, \alpha } \lesssim _s \varepsilon (1+\Vert \mathfrak {I}_{\delta } \Vert ^{\gamma , \mathcal {O}_0}_{s+\widehat{\sigma }}){,} \qquad |\Delta _{12} \mathtt {q}_6 |_{-1, p, \alpha }\lesssim _{p} \varepsilon \gamma ^{-1} (1+\Vert \mathfrak {I}_{\delta } \Vert _{p+\widehat{\sigma }}) \Vert i_1-i_2 \Vert _{p+\widehat{\sigma }}{,} \end{aligned}$$

and $\widehat{\mathcal {Q}}_{6}\in \mathfrak {L}_{\rho , p}$, for $s_0\le s\le \mathcal {S}$, satisfies

7.50 $$\begin{aligned}&\mathbb {M}^{\gamma }_{\widehat{\mathcal {Q}}_6}(s, \mathtt {b}) \lesssim _s \varepsilon (1+\Vert \mathfrak {I}_{\delta } \Vert _{s+\widehat{\sigma }}^{\gamma , \mathcal {O}_0}){,} \quad 0\le \mathtt {b}\le \rho -2{,} \end{aligned}$$

7.51 $$\begin{aligned}&\mathbb {M}_{ \Delta _{12} \widehat{\mathcal {Q}}_6 }(p, \mathtt {b}) \lesssim _{p} \varepsilon \gamma ^{-1} (1+\Vert \mathfrak {I}_{\delta } \Vert _{p+\widehat{\sigma }}) \Vert i_1-i_2 \Vert _{p+\widehat{\sigma }}{,}\qquad 0\le \mathtt {b}\le \rho -3 \end{aligned}$$

with $\widehat{\sigma }=\sigma _0+\sigma _9+\rho +s_1-s_0$ for some $\sigma _9$, possibly larger than $\sigma _8$ (recall (7.16) with $i=5$ and $s_1$ given in Proposition 3.6 in [33]).

#### Proof

The first order linear differential operator (recall (7.36), (7.40))

7.52 $$\begin{aligned} \omega \cdot \partial _{{\varphi }}-\big (1+\varepsilon ^2 c(\omega )+\varepsilon ^4 d(\xi )+a_5(\varphi , x) \big )\partial _x \end{aligned}$$

defined on $H_{S^{\perp }}^s(\mathbb {T}^{\nu +1})$ is associated to the vector field on $\mathbb {T}^{\nu +1}$

7.53 $$\begin{aligned} X_0:=\omega \cdot \frac{\partial }{\partial \varphi } - \big (1+\varepsilon ^2 c(\omega )+\varepsilon ^4 d(\xi ) +a_5(\varphi , x) \big )\frac{\partial }{\partial x}{.} \end{aligned}$$

For $\mathfrak {p}_{1}$ in (6.12) large enough, i.e. if $\mathfrak {p}_{1}\gg \sigma _0+s_1+\sigma _8$, and by (7.17) with $i=5$, we have

$$\begin{aligned} C(s_1)\,\gamma ^{-1} \Vert a_5 \Vert ^{\gamma , \mathcal {O}_{\infty }^{2 \gamma }}_{s_1}\le C(s_1) \varepsilon ^{4-3 a}=\delta ^* \ll 1, \end{aligned}$$

provided that $\varepsilon$ is small enough. This is the condition (7.12), hence Proposition 3.6 in [33] applies to the vector field (7.53). Thus there exist $\beta ^{(\infty )}$ and *m* such that the bounds (7.45), (7.48) hold. In particular the second bound in (7.45) follows by Lemma 3.7 in [33]. Moreover the operator (7.52) conjugated by the transformation

$$\begin{aligned} \mathcal {T}_{\beta ^{(\infty )}}:u(\varphi , x)\mapsto u(\varphi , x+\beta ^{(\infty )}(\varphi , x)){,} \end{aligned}$$

is associated to the vector field

$$\begin{aligned}&(\mathcal {T}_{\beta ^{(\infty )}})_* X_0=\omega \cdot \frac{\partial }{\partial \varphi }+ (\mathcal {T}_{\beta ^{(\infty )}})^{-1}\nonumber \\&\quad \Big (\omega \cdot \partial _{\varphi } \beta ^{(\infty )} -(1+\varepsilon ^2 c(\omega )+\varepsilon ^4 d(\xi )+a_5(\varphi , x)(1+\beta _x^{(\infty )})\Big )\frac{\partial }{\partial x}{,} \end{aligned}$$

and by Proposition 3.6 in [33] we have that

7.54 $$\begin{aligned} \omega \cdot \partial _{\varphi } \beta ^{(\infty )}-(1+\varepsilon ^2 c(\omega )+\varepsilon ^4 d(\omega )+a_5(\varphi , x))(1+\beta _x^{(\infty )})=-m{.} \end{aligned}$$

By Lemma 11.4 in [1] the function $\beta ^{(\infty )}$ satisfies the bound (7.45). By (7.45) and $\mathfrak {p}_1$ large enough, for $\varepsilon$ small enough, the function $\beta ^{(\infty )}$ satisfies the smallness condition of Lemma C.1, indeed

7.55 $$\begin{aligned}&\Vert \beta ^{(\infty )} \Vert ^{{\gamma }, \mathcal {O}_0}_{s_0+\sigma _1} \le C(s_1) \gamma ^{-1} \Vert a_5 \Vert ^{{\gamma }, \mathcal {O}_{\infty }^{2 \gamma }}_{s_0+\sigma _1+2\tau +4} {\mathop {\le }\limits ^{(7.17)}} C(s_1)\,\gamma ^{-1} \big ( \varepsilon ^{6} +\varepsilon \Vert \mathfrak {I}_{\delta }\Vert ^{{\gamma }, \mathcal {O}_0}_{s_0+\mathfrak {p}_1} \big )\nonumber \\&\quad {\mathop {\le }\limits ^{(6.12)}} C(s_1)\varepsilon ^{4- 3 a}{.} \end{aligned}$$

Hence ${\mathcal {B}}_6$ is well defined. By (7.17), (7.18), (7.19), $i=5$, the bounds (C.13), (C.14) hold with $\mathtt {k}_1\rightsquigarrow \varepsilon ^6$, $\mathtt {k}_2\rightsquigarrow \varepsilon$, $\mathtt {k}_3\rightsquigarrow \varepsilon \gamma ^{-1}$ and Proposition C.2 applies and the thesis follows. $\quad \square$

Let us define

7.56 $$\begin{aligned} {\mathcal {B}}:={\mathcal {B}}_{6}\circ {\mathcal {B}}_5\circ {\mathcal {B}}_4\circ {\mathcal {B}}_3\circ {\mathcal {B}}_2\circ {\mathcal {B}}_1{.} \end{aligned}$$

Then the Hamiltonian of the operator $\mathcal {L}_6$ is (recall (6.31), (7.43) and (7.47))

7.57 $$\begin{aligned} \mathcal {K}:=\mathsf {H}\circ {\mathcal {B}}^{-1}=\mathsf {H}_0+\varepsilon \,\mathcal {K}_1 +\varepsilon ^2\Big (\mathtt {Z}_0+\,\mathcal {K}_2\Big ) +\varepsilon ^3 \,\mathcal {K}_3+o(\varepsilon ^3) {.} \end{aligned}$$

Notice also that $\{\mathtt {H}_0, \mathtt {Z}_0\}_e=0$. The expansion (7.57) allows us, together with Remark 7.3, to give a more precise expression of the remainder $\mathcal {Q}_{6}$ in (7.47). This is the content of Lemma 7.6 in the next section.

### Linear Birkhoff normal form

The aim of this section is to eliminate $\mathcal {K}_1$, $\mathcal {K}_3$ and normalize the Hamiltonian $\mathcal {K}_2$ from (7.57). Our first point is that the $-1$ smoothing remainder $o(\varepsilon ^3)$ belongs to a special class of operators defined in Definition C.6 and denoted by $\mathfrak {C}_{-1}$ . It turns out that this class is preserved under the changes of variables used in the linear Birkhoff normal form procedure (see Lemmata C.8, C.9).

#### Remark 7.5

In the following steps of linear Birkhoff normal form we shall use the relation

7.58 $$\begin{aligned} \sum _{i=1}^{\nu } \overline{\jmath }_i\ell _i+j'-j=0 {,} \qquad \text{ if }\,\,\,\,|\ell |\le 3,\,\,\,\,\, \forall j, \,j'\in S^c{,} \end{aligned}$$

which holds by the conservation of momentum.

#### Lemma 7.6

Recall (7.47). We have

7.59 $$\begin{aligned} \mathcal {L}_6 =\Pi _S^{\perp }\big (\omega \cdot \partial _{\varphi }-m J-\varepsilon X_{\mathcal {K}_1}-\varepsilon ^2 X_{\mathcal {K}_2}-\varepsilon ^3 X_{\mathcal {K}_3}+\mathfrak {R}\big ) \end{aligned}$$

where $$X_{\mathcal {K}_i}:=J \nabla \mathcal {K}_i$$, $$i=1, 2, 3, $$ are almost diagonal and in $$\mathfrak {C}_{-1}(\mathcal {O}^{2\gamma }_{\infty })$$ (recall Definitions 6.4 and C.6) satisfying

7.60 $$\begin{aligned} \mathbb {B}^{{\gamma }}_{\varepsilon ^{k} J \nabla \mathcal {K}_k}(s) \le \varepsilon ^{k} C(s){,} \quad k=1,2,3{.} \end{aligned}$$

The remainder $\mathfrak {R}$ belongs to $\mathfrak {C}_{-1}(\mathcal {O}^{2\gamma }_{\infty })$ and satisfies

7.61 $$\begin{aligned} \mathfrak {R}&:=\mathcal {Q}_6 +\varepsilon X_{\mathcal {K}_1} +\varepsilon ^2 X_{\mathcal {K}_2} +\varepsilon ^3 X_{\mathcal {K}_3}{.} \end{aligned}$$

7.62 $$\begin{aligned} \mathbb {B}^{\gamma }_{ \mathfrak {R}}(s)&\lesssim _s \varepsilon ^{4-3 a}+\varepsilon \gamma ^{-1}\Vert \mathfrak {I}_{\delta } \Vert ^{\gamma , \mathcal {O}_0}_{s+\widehat{\sigma }}{,} \qquad \mathbb {B}_{ \Delta _{12} \mathfrak {R} }(s_0)\lesssim \varepsilon \gamma ^{-1} \Vert i_1-i_2 \Vert _{s_0+\widehat{\sigma }}{,} \end{aligned}$$

for $\widehat{\sigma }$ given in Proposition 7.4.

#### Proof

By the discussion of Sect. 7.1$\mathcal {K}_i$, $i=1,2,3$, are of the form (6.35) with $f_{i}=0$ for $i=1,2,3$. Hence the vector field $X_{\mathcal {K}_i}$ are pseudo differential of order $-1$ up to a finite rank term. In addition, they are almost diagonal by (6.34) and the momentum condition (7.58). By Lemma C.8-(ii) $\varepsilon X_{\mathcal {K}_1}, \varepsilon ^2 X_{\mathcal {K}_2}, \varepsilon ^3 X_{\mathcal {K}_3}$ belong to $\mathfrak {C}_{-1}$ and, by (7.27), (7.26), (7.35), (7.37), (7.41), satisfy (7.60). By Proposition 7.4, the choice of $\rho$ as in (7.15) and by Lemma C.8-(*i*) taking $p=s_0$ and $\mathfrak {p}_1$ large enough, $\mathcal {Q}_6\in \mathfrak {C}_{-1}$. Thus $\mathfrak {R}\in \mathfrak {C}_{-1}$.

Note that only $\mathcal {Q}_6$ in (7.61) depend on the torus embedding $i_{\delta }$, then the second bound in (7.62) follows by Lemma C.8-(*i*), (*ii*), (7.49) and (7.51). To prove the first bound in (7.62) we reason as follows.

By (7.45) and (7.17) with $i=5$ we have that

7.63 $$\begin{aligned} \Vert \beta ^{(\infty )}\Vert _s^{{\gamma }, \mathcal {O}_{\infty }^{2 {\gamma }}} \lesssim _s \varepsilon ^6 \gamma ^{-1}+\varepsilon \gamma ^{-1}\Vert \mathfrak {I}_{\delta } \Vert ^{{\gamma }, \mathcal {O}_{0}}_{s+2\tau +4+\sigma _0+\sigma _8}, \quad \Vert \beta ^{(\infty )}\Vert _{s_0}^{{\gamma }, \mathcal {O}_{\infty }^{2 {\gamma }}}\lesssim \varepsilon ^{4-3 a}. \end{aligned}$$

Then the map $\mathcal {B}_6$ leaves invariant (using Remark 7.2) the terms of size $\varepsilon , \varepsilon ^2, \varepsilon ^3$ in $\mathcal {L}_5$, and hence, by Remark 7.3, those terms in $\mathcal {Q}_6$ are given by $-\varepsilon J \nabla \, \mathcal {K}_1, -\varepsilon ^2 J \nabla \, \mathcal {K}_2, -\varepsilon ^3 J \nabla \, \mathcal {K}_3$.

From the proof of the bounds (C.18), (C.20) in Proposition C.2 one can notice that the operators $\mathrm{Op}(\mathtt {q}_6)$ and $\widehat{\mathcal {Q}}_6$ admit a “formal” expansion in $\beta ^{(\infty )}$ (by expanding the flow in $\tau$). Of course, by the discussion above, the biggest term in $\mathfrak {R}$ are the ones which are linear in $\beta ^{(\infty )}$. Such term comes from the conjugation of $\mathcal {L}_5$ under the map $\mathcal {B}_6$, more precisely from the conjugation of

$$\begin{aligned} (J-\partial _x)\circ (1+\varepsilon ^2 c(\omega )+\varepsilon ^4 d(\omega )+a_5(\varphi , x)). \end{aligned}$$

We refer to the formula (3.11) in Proposition 3.1 of [33] to see the term bounded by the norm of $\beta ^{(\infty )}$. Comparing the bounds (7.63) and (7.42) one can deduce the first bound in (7.62). $\quad \square$

In order to normalize the vector fields $\varepsilon ^i J \nabla \mathcal {K}_i$ we will look for changes of coordinates $\Upsilon _i$ generated as one-time flow of quadratic Hamiltonians $H_{\mathtt {A}_i}$ described by almost diagonal matrices $\mathtt {A}_i$ (see (C.47), (C.48) ,(C.49), (C.50)). We remark that the Hamiltonian $\varepsilon ^2\mathtt {Z}_0$ is left invariant by these changes of coordinates, since $\{ \mathtt {H}_0, \mathtt {Z}_0\}_e=0$. At any step of the procedure we shall verify that $J \mathtt {B}_i$ (see (C.49), (C.50)) are almost diagonal and belong to $\mathfrak {C}_{-1}$ in order to apply Lemma C.13, which guarantees well-posedness and tame estimates of $\Upsilon _i$.

**Step one (order**$\varepsilon$**)** At this step we want to eliminate $\varepsilon X_{\mathcal {K}_1}$ from (7.59). We have

7.64 $$\begin{aligned} \mathcal {K}^{(1)}&:=\mathcal {K}\circ \Upsilon _1^{-1}= \,\mathsf {H}_0+\varepsilon \mathcal {K}^{(1)}_1 +\varepsilon ^2\Big (\mathtt {Z}_0+ \mathcal {K}^{(1)}_2\Big )+\varepsilon ^3 \mathcal {K}_3^{(1)}+o(\varepsilon ^3){,} \end{aligned}$$

7.65 $$\begin{aligned} \mathcal {K}^{(1)}_1&:= \mathcal {K}_1+\{H_{\mathtt {A}_1},\mathsf {H}_0\}_e= \mathcal {K}_1+\overline{\omega }\cdot \partial _{\varphi } H_{\mathtt {A}_1} +\{ H_{\mathtt {A}_1}, \mathsf {H}_0 \}{,}\nonumber \\ \mathcal {K}^{(1)}_2&:= \mathcal {K}_2+\frac{1}{2}\{ H_{\mathtt {A}_1}, \{H_{\mathtt {A}_1},\mathsf {H}_0\}_e\}_e +\{H_{\mathtt {A}_1}, \mathcal {K}_1\}_e\nonumber \\ \,&=\mathcal {K}_2+\frac{1}{2}\{ H_{\mathtt {A}_1}, \mathcal {K}_1^{(1)}\} +\frac{1}{2}\{H_{\mathtt {A}_1}, \mathcal {K}_1\}{.} \end{aligned}$$

We choose $\mathtt {A}_1$ such that

7.66 $$\begin{aligned} \mathcal {K}_1^{(1)}=\overline{\omega }\cdot \partial _{\varphi } H_{\mathtt {A}_1} +\{ H_{\mathtt {A}_1}, \mathsf {H}_0 \}+\mathcal {K}_1=0. \end{aligned}$$

Recalling that $\mathcal {K}_1:=\mathsf {H}_1^{(1)}$, we have (see (7.27), (7.28))

7.67 $$\begin{aligned} \mathcal {K}_1(u)=\sum _{j, j'\in S^c} (\mathfrak {B}_1)_j^{j'}(\varphi )\,u_{j'}\,u_{-j}. \end{aligned}$$

Then we choose $\mathtt {B}_1=\mathfrak {B}_1$ in (C.49). By recalling the definition of $\mathfrak {B}_1$ in (7.27) it is easy to see that $J \mathtt {B}_1\in \mathfrak {C}_{-1}$, since it is a pseudo differential operator of order $-1$. Moreover it is almost diagonal because *J*, $3\Lambda \partial _x$ are diagonal operators and $\beta _1$ is a function supported on the finite set *S*.

Given $X\in \mathfrak {C}_{-1}$ to shorten the notation in the following lemma we write (recall (2.3))

7.68 $$\begin{aligned} \mathrm {ad}_{X}[\cdot ]:=[X,\cdot ]{.} \end{aligned}$$

Under this notation we have the following lemma.

#### Lemma 7.7

The transformed operator is (recall (7.59), (7.64))

7.69 $$\begin{aligned} \mathcal {L}_7 h:=\Upsilon _1 \mathcal {L}_6 \Upsilon _1^{-1} =\Pi _S^{\perp } \Big (\omega \cdot \partial _{\varphi }-m J-\varepsilon ^2 X_{\mathcal {K}_2^{(1)}}-\varepsilon ^3 X_{\mathcal {K}_3^{(1)}}+\mathcal {R}_7\Big ) \end{aligned}$$

where

7.70 $$\begin{aligned} X_{\mathcal {K}_2^{(1)}}&:=J\nabla \mathcal {K}_2^{(1)} =X_{\mathcal {K}_2}+\mathrm {ad}_{X_{\mathtt {A}_1}}[X_{\mathcal {K}_1}]+\frac{1}{2}\mathrm {ad}^2_{X_{\mathtt {A}_1}}[\omega \cdot \partial _{\varphi }-m J], \end{aligned}$$

7.71 $$\begin{aligned} X_{\mathcal {K}_3^{(1)}}&:=J\nabla \mathcal {K}_3^{(1)} = X_{\mathcal {K}_3}+\mathrm {ad}_{X_{\mathtt {A}_1}}[X_{\mathcal {K}_2}]+\frac{1}{2}\mathrm {ad}^2_{X_{\mathtt {A}_1}}[X_{\mathcal {K}_1}]+\frac{1}{6}\mathrm {ad}^3_{X_{\mathtt {A}_1}}[\omega \cdot \partial _{\varphi }-m J], \end{aligned}$$

the operator $\mathcal {R}_{7}\in \mathfrak {C}_{-1}$ with

7.72 $$\begin{aligned} \mathbb {B}^{{\gamma }}_{\mathcal {R}_7}(s) \! \lesssim _s \! \varepsilon ^{4-3 a}\!+\!\varepsilon {\gamma }^{-1} \Vert \mathfrak {I}_{\delta }\Vert _{s+\tilde{\sigma }}^{\gamma , \mathcal {O}_0}, \quad \mathbb {B}_{\Delta _{12} \mathcal {R}_7 }(s_0) \!\lesssim \! \varepsilon \gamma ^{-1}(1+\Vert \mathfrak {I}_{\delta }\Vert _{s_0+\tilde{\sigma }}) \Vert i_1\!-\! i_2\Vert _{s_0+\tilde{\sigma }}, \end{aligned}$$

for some $\tilde{\sigma }\ge \widehat{\sigma }$ (recall the loss of regularity in (7.49), (7.50), (7.51)).

#### Proof

By using (C.58) we have that formulæ (7.70), (7.71) hold and that

7.73 $$\begin{aligned} \mathcal {R}_7:&=\mathfrak {R}+\varepsilon \,\mathrm {ad}_{X_{\mathtt {A}_1}}[\varepsilon ^3 X_{\mathcal {K}_3}+\mathfrak {R}]+\frac{\varepsilon ^2}{2}\mathrm {ad}^2_{X_{\mathtt {A}_1}}[-\varepsilon ^2 X_{\mathcal {K}_2}-\varepsilon ^3 X_{\mathcal {K}_3}+\mathfrak {R}]\nonumber \\&+\frac{\varepsilon ^3}{6}\mathrm {ad}^3_{X_{\mathtt {A}_1}}[-\varepsilon X_{\mathcal {K}_1} -\varepsilon ^2 X_{\mathcal {K}_2}-\varepsilon ^3 X_{\mathcal {K}_3} +\mathfrak {R}]+\sum _{k\ge 4} \frac{\varepsilon ^k}{k!}\mathrm {ad}^k_{X_{\mathtt {A}_1}}[\mathcal {L}_6]. \end{aligned}$$

For $Y \in \mathfrak {C}_{-1}$ define $\mathcal {Z}_n:=\sum _{k\ge n} \frac{\varepsilon ^k}{k!}\mathrm {ad}_{X_{\mathtt {A}_1}}^k[Y]$ for any $n\ge 1$. By Lemma C.9, and using (C.54), we deduce that

7.74 $$\begin{aligned} \mathbb {B}^{{\gamma }}_{\mathcal {Z}_n}(s)&\lesssim _s C(s, n)\mathbb {B}^{{\gamma }}_Y(s_0)+ C(s_0, n) \mathbb {B}^{{\gamma }}_Y(s)\nonumber \\ \mathbb {B}_{\Delta _{12} \mathcal {Z}_n}(s_0)&\lesssim C(s_0, n)\,\Big (\mathbb {B}_{Y}(s_0)+\mathbb {B}_{\Delta _{12} Y}(s_0) \Big ) \end{aligned}$$

for $\varepsilon$ small enough. In (7.73) there are terms of the form $\mathrm {ad}^k_{X_{\mathtt {A}_1}}[Y]$, for some $k\ge 1$, with $Y=X_{\mathcal {K}_1}, X_{\mathcal {K}_2}, X_{\mathcal {K}_3}, \mathfrak {R}$ which belong to $\mathfrak {C}_{-1}$ by Lemmata C.8 and 7.6. We note that by (7.66)

7.75 $$\begin{aligned} \mathrm {ad}_{X_{\mathtt {A}_1}}[\omega \cdot \partial _{\varphi }-m J]=-\varepsilon X_{\mathcal {K}_1}-(\omega -\overline{\omega })\cdot \partial _{\varphi } X_{\mathtt {A}_1}-(m-1) [X_{\mathtt {A}_1}, J]\in \mathfrak {C}_{-1} \end{aligned}$$

since $\mathtt {A}_1$ is almost diagonal. Hence $(\omega -\overline{\omega })\cdot \partial _{\varphi }X_{\mathtt {A}_1}, [X_{\mathtt {A}_1}, J] \in \mathfrak {C}_{-1}$ (see the proof of Lemma C.13) and by Lemma C.8-(*iii*) the remainder $\mathcal {R}_7\in \mathfrak {C}_{-1}$. By (7.73), (C.43), (C.44), (7.74), (7.75), (7.60), (7.62) and the fact that $|\omega -\overline{\omega } |\lesssim \varepsilon ^2$ we get the bounds (7.72). $\quad \square$

**Step two (order**$\varepsilon ^2$**)** At this step we want to normalize $\varepsilon ^2 X_{\mathcal {K}_2^{(1)}}$ from (7.69). We have

7.76 $$\begin{aligned} \mathcal {K}^{(2)}:=\mathcal {K}^{(1)}\circ \Upsilon _2^{-1}= \,\mathsf {H}_0+\varepsilon ^2\Big ( \mathtt {Z}_0+ \mathcal {K}^{(2)}_2\Big ) +\varepsilon ^3 \mathcal {K}_3^{(2)}+o(\varepsilon ^3){,} \qquad \mathcal {K}^{(2)}_2:=\{ H_{\mathtt {A}_2}, \mathsf {H}_0\}_e +\mathcal {K}^{(1)}_2{,} \end{aligned}$$

where $\mathcal {K}_2^{(1)}$ is given in (7.65) (see also (7.70)). We choose $\mathtt {A}_2$ in order to solve the following equation

7.77 $$\begin{aligned} \overline{\omega }\cdot \partial _{\varphi } H_{\mathtt {A}_2}+\{H_{\mathtt {A}_2}, \mathsf {H}_0\}=\mathcal {K}^{(1)}_2-\Pi _{\mathrm{Ker}(\mathsf {H}_0)}\mathcal {K}^{(1)}_2{.} \end{aligned}$$

Hence we choose $\mathtt {B}_2= \nabla \Pi _{\mathrm{Rg}(\mathsf {H}_0)} \mathcal {K}^{(1)}_2(u)$ in (C.49). Note that $X_{\mathcal {K}_2}$ is pseudo differential of order $-1$ and $J \mathtt {A}_1$, $X_{\mathcal {K}_1}$ belong to $\mathfrak {C}_{-1}$ and so also their Poisson brackets. Hence $J \mathtt {B}_2\in \mathfrak {C}_{-1}$. By Remark 6.5 we have that $J \mathtt {B}_2$ is also almost diagonal.

In order to perform the third step in the linear BNF we need to explicitly compute the corrections $O(\varepsilon ^2)$ coming from $\Pi _{\mathrm{Ker}(\mathsf {H}_0)}\mathcal {K}^{(1)}_2$. The point is that a priori, it is not clear whether the resonant terms $\Pi _{\mathrm{Ker}(\mathsf {H}_0)}\mathcal {K}^{(1)}_2$ are supported only on trivial resonances. Our approach is then to show that the normal form we obtain must necessarily coincide with the formal one, which is relatively easy to compute.

#### Definition 7.8

Recalling the notations used in Sect. 3, we denote by $\Pi ^{d_z\le k}$, respectively $\Pi ^{d_z=k}$, the projector of a homogenous Hamiltonian of degree *n* on the monomials with degree less or equal than *k*, respectively equal *k*, in the normal variable *z*, i.e.

$$\begin{aligned} \Pi ^{d_z \le k} H^{(n)}:=H^{(n, \le k)}, \quad \Pi ^{d_z = k} H^{(n)}:=H^{(n, k)}. \end{aligned}$$

We denote by $\Pi _{\mathrm {triv}}$ the projection onto trivial resonances (of the form (3.11)), i.e. monomials of the form

$$\begin{aligned} u_{j}u_{-j}u_i u_{-i}\ldots u_{k}u_{-k}. \end{aligned}$$

The following proposition allows to easily compute the resonant terms $\Pi _{\mathrm{Ker}(\mathsf {H}_0)}\mathcal {K}^{(1)}_2$ in (B.17).

#### Theorem 7.9

(Normal form identification). Consider the symplectic change of coordinates $A_{\varepsilon }$ in (4.7). Then

7.78 $$\begin{aligned} \Pi ^{d_z=2}\Pi _{\mathrm{Ker}(\mathtt {H}_0)} \Big (\mathtt {Z}_0+ \mathcal {K}^{(1)}_2\Big )= \Big [\Pi _{\mathrm{\mathrm triv}}\Pi ^{d_z=2} \Big ( \frac{1}{2}\{ \mathfrak {F}^{(3)} , H^{(3)}\} \Big )\Big ]\circ A_{{1{|_{ \begin{array}{c} y=0\\ \theta ={\varphi } \end{array}}}}}{,} \end{aligned}$$

where $A_1:=A_{{\varepsilon }_{|_{\varepsilon =1}}}$, $\mathtt {H}_0$ is in (6.32) and we set (recalling (2.4)) $\mathfrak {F}^{(3)}:=[\mathrm {ad}_{H^{(2)}}]^{-1}H^{(3)}$ with $H^{(3)}$ in (3.1).

#### Proof

The proof is postponed to the “Appendix B”. $\quad \square$

As a consequence of the identification above, we have, by (7.78), (3.21), (3.1),

7.79 $$\begin{aligned} \Big (\frac{c(\omega )}{2}\sum _{j\in S^c} |u_j |^2+\Pi _{\mathrm{Ker}(\mathsf {H}_0)}\mathcal {K}^{(1)}_2\Big )=\frac{1}{2}\sum _{j\in S^c} \mathfrak {l}_j |u_j |^2 \end{aligned}$$

where (recall (1.8))

7.80 $$\begin{aligned} \mathfrak {l}_j:= & {} \sum _{j_2\in S}\frac{{\lambda }(j_2+j)}{{\lambda }(j_2)+{\lambda }(j)-{\lambda }(j_2+j)}\,\xi _{j_2}\nonumber \\= & {} \frac{2}{3} \sum _{j_2\in S^+} \,\frac{(1+j_2^2)(1+j^2)(2+j_2^2+j^2)}{(3+j_2^2-j_2 j+j^2)(3+j_2^2+j_2 j +j^2)}\xi _{j_2}. \end{aligned}$$

We define the diagonal operator (recall (7.36))

7.81 $$\begin{aligned} \mathfrak {D}:=\mathfrak {D}(\xi )=\mathrm {diag}\,\,(\mathrm {i}\kappa _j)_{j\in S^c}, \qquad \kappa _j={\lambda }(j)\,\big (\mathfrak {l}_j-c(\omega )\big )\in \mathbb {R}. \end{aligned}$$

#### Lemma 7.10

We have

7.82 $$\begin{aligned} \kappa _j={\lambda }(j)\,\big (\mathfrak {l}_j-c(\omega )\big ){,}\quad |j |\,|\kappa _j |\le C \qquad \forall j\in S^c, \end{aligned}$$

for an appropriate constant $C>0$ depending on the set *S*.

#### Proof

Recalling the definitions (7.36) and (7.80) we have, for $j\in S^c$,

7.83 $$\begin{aligned} \mathfrak {l}_j-c(\omega )=-\frac{2}{3}\sum _{j_0\in S^+} \,\frac{(1+j_0^2)(7+5 j_0^2+j_0^4+3 j^2)}{(3+j_0^2-j_0 j+j^2)(3+j_0^2+j_0 j +j^2)}\xi _{j_0}=\frac{P(j)}{Q(j)}. \end{aligned}$$

It is easy to prove that $$|{\lambda }(j) |\le 4 |j |$$, $$3+j_0^2+j^2\pm j_0 j\ge \frac{3}{4} j^2 $$ and $$(1+j_0^2)(7+5 j_0^2+j_0^4+3 j^2)\le 14 j_0^6 j^2$$. Hence $$ |\kappa (j) |\lesssim \frac{|j |}{j^2} \sum _{j_0\in S^+} j_0^6. $$$$\quad \square $$

#### Lemma 7.11

The transformed operator is (recall (7.69))

7.84 $$\begin{aligned} \mathcal {L}_8 :=\Upsilon _2 \mathcal {L}_7 \Upsilon _2^{-1} =\Pi _S^{\perp }\Big (\omega \cdot \partial _{\varphi }-m J-\varepsilon ^2 \mathfrak {D}(\xi )-\varepsilon ^3 X_{\mathcal {K}_3^{(2)}} +\mathcal {R}_8 \Big ) \end{aligned}$$

where $\mathcal {K}_3^{(2)}=\mathcal {K}_3^{(1)}$, $\mathfrak {D}(\xi )$ is the diagonal operator of order $-1$ defined in (7.81), $\mathcal {R}_8\in \mathfrak {C}_{-1}$ satisfies

7.85 $$\begin{aligned} \mathbb {B}^{{\gamma }}_{\mathcal {R}_8}(s) \lesssim _s \varepsilon ^{4-3 a}+\varepsilon {\gamma }^{-1} \Vert \mathfrak {I}_{\delta }\Vert _{s+\tilde{\sigma }}^{\gamma , \mathcal {O}_0}, \quad \mathbb {B}_{\Delta _{12} \mathcal {R}_8 }(s_0) \lesssim \varepsilon \gamma ^{-1}(1+\Vert \mathfrak {I}_{\delta }\Vert _{s_0+\tilde{\sigma }}) \Vert i_1-i_2\Vert _{s_0+\tilde{\sigma }}, \end{aligned}$$

for some $\tilde{\sigma }$ possibly larger than the one in Lemma 7.7.

#### Proof

The proof follows by using the same arguments of the proof of Lemma 7.7. In particular, expanding the left hand side of (7.84) using (C.58) we get

7.86 $$\begin{aligned} \mathcal {R}_8 :=\mathcal {R}_7+\varepsilon ^2\mathrm {ad}_{X_{\mathtt {A}_2}}[-\varepsilon ^2 \mathfrak {D}(\xi )-\varepsilon ^3 X_{\mathcal {K}_3^{(2)}} +\mathcal {R}_8]+\sum _{k\ge 2}\frac{\varepsilon ^{2 k}}{k!}\mathrm {ad}_{X_{\mathtt {A}_2}}[\mathcal {L}_7]{.} \end{aligned}$$

By (7.77) and Theorem 7.9 we have that

$$\begin{aligned} \mathrm {ad}_{X_{\mathtt {A}_2}}[\omega \cdot \partial _{\varphi }-m J]+\mathcal {K}^{(1)}_2=-\mathfrak {D}(\xi )-(\omega -\overline{\omega })\cdot \partial _{\varphi }X_{\mathtt {A}_2}-(m-1)[X_{\mathtt {A}_2}, J]. \end{aligned}$$

By (7.10) $\mathfrak {D}(\xi )\in \mathfrak {C}_{-1}$ and by the fact that $\mathtt {A}_2$ is almost diagonal we have that $(\omega -\overline{\omega })\cdot \partial _{\varphi }X_{\mathtt {A}_2}, [X_{\mathtt {A}_2}, J]\in \mathfrak {C}_{-1}$. Then $\mathcal {R}_{8}\in \mathfrak {C}_{-1}$. The bounds (7.85) are obtained by using the estimates (C.43), (C.44), (7.74), (C.54) and (7.72). $\quad \square$

**Step three (order**$\varepsilon ^3$**)** At this step we eliminate $\varepsilon ^3 X_{\mathcal {K}^{(2)}_3}$ from (7.84). Recalling that $\mathcal {K}^{(2)}_3$ is given in Lemma 7.11, we have

7.87 $$\begin{aligned} \mathcal {K}^{(3)}&:=\mathcal {K}^{(2)}\circ \Upsilon ^{-1}_3= \,\mathsf {H}_0+\varepsilon ^2 \mathcal {K}^{(2)}_2+\varepsilon ^3 \mathcal {K}^{(3)}_3+o(\varepsilon ^3), \nonumber \\ \mathcal {K}^{(3)}_3&:=\{ H_{\mathtt {A}_3}, \mathsf {H}_0+\varepsilon ^2 \mathbb {A}\xi \cdot \eta +\frac{\varepsilon ^2}{2}\sum _{j\in S^c} \mathfrak {l}_j(\xi ) z_j \,z_{-j}\}_e+\mathcal {K}^{(2)}_3{.} \end{aligned}$$

Note that we consider in the normal form also the $\varepsilon ^2$-terms. We want to solve the equation

7.88 $$\begin{aligned} \mathcal {D}_{\overline{\omega } +\varepsilon ^2 \mathbb {A}\xi } H_{\mathtt {A}_3} +\{ H_{\mathtt {A}_3}, \mathsf {H}_0 + \frac{\varepsilon ^2}{2}\sum _{j\in S^c} \mathfrak {l}_j(\xi ) z_j \,z_{-j}\}+ \mathcal {K}_3^{(2)}=0{.} \end{aligned}$$

Hence we choose the matrix $\mathtt {B}_3:=\nabla \mathcal {K}^{(1)}_3(u)$ (note that $\mathcal {K}^{(1)}_3=\mathcal {K}^{(2)}_3$). Recalling (7.71) it is easy to see that $J \mathtt {B}_3$ is sum of Lie brackets of elements of $\mathfrak {C}_{-1}$, hence by Lemma C.8 it belongs to $\mathfrak {C}_{-1}$. By the fact that $\mathtt {A}_1$ is almost diagonal and by Remark 6.5 we have that $J \mathtt {B}_3$ is almost diagonal.

#### Lemma 7.12

The transformed operator is (recall (7.69))

7.89 $$\begin{aligned} \mathcal {L}_9 :=\Upsilon _3 \mathcal {L}_8 \Upsilon _3^{-1} = \Pi _S^{\perp }\Big (\omega \cdot \partial _{\varphi } -m J-\varepsilon ^2 \mathfrak {D}(\xi ) +\mathcal {R}_9 \Big ) \end{aligned}$$

where $\mathfrak {D}(\xi )$ is the diagonal operator of order $-1$ defined in (7.81), $\mathcal {R}_9\in \mathfrak {C}_{-1}$ satisfies

7.90 $$\begin{aligned} \mathbb {B}^{{\gamma }}_{\mathcal {R}_9}(s) \lesssim _{s} \varepsilon ^{4- 3 a}+ \varepsilon {\gamma }^{-1} \Vert \mathfrak {I}_{\delta }\Vert _{s+\tilde{\sigma }}^{\gamma , \mathcal {O}_0}{,} \qquad \mathbb {B}_{\Delta _{12} \mathcal {R}_9 }(s_0) \lesssim \varepsilon {\gamma }^{-1}\Vert i_1-i_2\Vert _{s_0+\tilde{\sigma }}{,} \end{aligned}$$

for some $\tilde{\sigma }$ possibly larger than the one in Lemma 7.11.

#### Proof

The proof follows the same arguments used for proving Lemma 7.7. By (C.58) we deduce

7.91 $$\begin{aligned} \mathcal {R}_9 :=\mathcal {R}_8+\varepsilon ^3\mathrm {ad}_{X_{\mathtt {A}_3}}[-\varepsilon ^3 X_{\mathcal {K}_3^{(2)}}+\mathcal {R}_8]+\sum _{k\ge 2} \frac{1}{k!} \mathrm {ad}_{X_{\mathtt {A}_3}}^k[\mathcal {L}_8]{.} \end{aligned}$$

We note that by (7.88) we have (recall (4.5) and (7.36))

$$\begin{aligned} \mathrm {ad}_{X_{\mathtt {A}_3}}[\omega \cdot \partial _{\varphi }-mJ-\varepsilon ^2 \mathfrak {D}(\xi )]= & {} \varepsilon ^3 X_{\mathcal {K}_3^{(2)}}+(\omega -\overline{\omega }-\varepsilon ^2 \mathbb {A}\xi )\cdot \partial _{\varphi } \mathtt {A}_{3}\\&-(m-1-\varepsilon ^2 c(\omega ))[X_{\mathtt {A}_3}, J] \in \mathfrak {C}_{-1}{,} \end{aligned}$$

since $\mathtt {A}_3$ is almost diagonal . Hence the bounds (7.90) follows by (C.54), (7.85) and by using Lemma C.9. $\quad \square$

#### Proof of Theorem 7.1

We choose $\mu _1=\tilde{\sigma }$ given in Lemma 7.12. We consider *p* and $\mathfrak {p}_1$ so that

7.92 $$\begin{aligned} s_0+\mathfrak {p}_1-\widehat{\sigma }\ge p\ge s_0{,}\quad \sigma _{9}+\sigma _0+(s_1-s_0)+\sigma _1+\rho +1\le \tilde{\sigma }\, , \qquad \tilde{\sigma }\le \mathfrak {p}_1{,} \end{aligned}$$

where $\tilde{\sigma }$ is the loss of regularity in Lemma 7.12, $\sigma _0$ has been introduced in Sect. 7, see estimates (6.17)–(6.22), $\sigma _1>0$ and $s_1$ are given respectively in Lemma C.1 and in Proposition 3.6 in [33].

We define the map (recall (C.48), (7.56))

$$\begin{aligned} \Upsilon :=\Upsilon _3 \circ \Upsilon _2 \circ \Upsilon _1\circ \mathcal {B}{.} \end{aligned}$$

By Proposition 7.4 the map $\mathcal {B}$ is defined for $\omega \in \mathcal {O}^{2{\gamma }}_{\infty }$ (see (7.3)), and so also $\Upsilon$. By (7.26), (7.37), (7.41), (C.55), (C.54), (7.45), (7.17) (with $i=5$) and Lemma C.1 we have (7.9).

The result follows by setting $\mathcal {L}:=\mathcal {L}_9$ (see (7.89)), $\mathcal {P}_0:=\mathcal {R}_9$, and *m* is the constant given by Proposition 7.4. Indeed (7.48) implies (7.2) and (7.6). Moreover, by Lemma 7.12, we have that $\mathcal {P}_0\in \mathfrak {C}_{-1}$ and satisfies (7.90). Lemma C.10 implies that $\mathcal {P}_0$ is $-1$-modulo tame together with $\mathtt {b}_0$ derivatives in the variable $\varphi$ (recall the Definition C.32 and the fact that $\gamma ^{3/2}<{\gamma }$). The bounds (7.7), (7.8) follow from (C.45)–(C.46) , the definition of $\mathbb {B}^{{\gamma }}_{\mathcal {P}_0}(s)$ (see (C.32),(C.34)) and (7.90). By Lemma 7.10 we deduce (7.5).

$$\square $$

### KAM reducibilty and Inversion of the linearized operator

In this subsection we prove the claim (6.9) by diagonalizing the operator $\mathcal {L}$ in (7.4). We first write

7.93 $$\begin{aligned} \mathcal {L}:= & {} \omega \cdot \partial _{{\varphi }}-\mathbf {M}_0, \quad \mathbf {M}_0 := \mathcal {D}_0+\mathcal {P}_0{,}\quad \mathcal {D}_0 := \mathrm{diag}(\mathrm {i} \, d_j^{(0)})_{j\in S^c}{,}\nonumber \\ d_j^{(0)}:= & {} d_j^{(0)}(\omega ) = m(\omega ){\lambda }(j)+\varepsilon ^2 \kappa _j(\omega ). \end{aligned}$$

Notice that (by the smallness condition (7.94)) Proposition 4.1 in [33] applies to the operator $\mathcal {L}$ in (7.4). Hence by following almost word by word the proof of Theorem 1.7 in [33] one has the following.

#### Theorem 7.13

(Reducibility). Fix $\tilde{{\gamma }}\in [\gamma ^{3/2}/4, 4\gamma ^{3/2}]$. Assume that $\omega \mapsto i_{\delta }(\omega )$ is a Lipschitz function defined on $\mathcal {O}_0\subseteq \Omega _{\varepsilon }$ (recall (6.3)), satisfying (6.12) with $\mathfrak {p}_1 \ge \mu _1$ where $\mu _1:=\mu _1(\nu )$ is given in Proposition 7.1. There exist $\delta _0\in (0, 1)$, $N_0>0$, $C_0>0$, such that, if (recall that by (4.8) ${\gamma }= \varepsilon ^{2+a}$)

7.94 $$\begin{aligned} N_0^{C_0} \varepsilon ^{4-3 a} \gamma ^{-3/2}=N_0^{C_0} \varepsilon ^{1-(9/2) a}\le \delta _0, \end{aligned}$$

then the following holds.

(i) **(Eigenvalues)** For all $\omega \in \Omega _{\varepsilon }$ there exists a sequence^4^
  7.95 $$\begin{aligned} d_j^{\infty }(\omega ):=d_j^{\infty }(\omega , i_{\delta }(\omega )):=m(\omega , i_{\delta }(\omega ))\,{\lambda }(j)+\varepsilon ^2 \kappa _j (\omega ) + r_j^{\infty }(\omega , i_{\delta }(\omega )), \quad j\in S^c, \end{aligned}$$
  with *m* and $\kappa _j$ in (7.48) and (7.81) respectively. Furthermore, for all $j\in S^c$
  7.96 $$\begin{aligned} \sup _j \langle j\rangle |r_j^{(\infty )}|^{\gamma ^{3/2}} \lesssim \varepsilon ^{4-3 a}, \qquad r_j^{\infty }=-r^{\infty }_{-j}. \end{aligned}$$
  All the eigenvalues $\mathrm {i} d_j^{\infty }$ are purely imaginary.
(ii) **(Conjugacy)** For all $\omega$ in the set
  7.97 $$\begin{aligned}&\Omega ^{2 {\tilde{\gamma }}}_{\infty } :=\Omega ^{2 \tilde{\gamma }}_{\infty }(i_{\delta })\nonumber \\&\quad :=\left\{ \omega \in \mathcal {O}^{2\gamma }_{\infty } : |\omega \cdot \ell \!+\! d_j^{\infty }(\omega )\!-\! d_k^{\infty }(\omega )|\ge \frac{2 {\tilde{\gamma }}}{\langle \ell \rangle ^{\tau }}, \,\,\forall \ell \!\in \!\mathbb {Z}^{\nu }, \,\,\forall j, k\in S^c,\, j\ne k \right\} \nonumber \\ \end{aligned}$$
  there is a real, bounded, invertible, linear operator $\Phi _{\infty }(\omega ):H^s_{S^{\perp }}(\mathbb {T}^{\nu +1})\rightarrow H_{S^{\perp }}^s(\mathbb {T}^{\nu +1})$, with bounded inverse $\Phi _{\infty }^{-1}(\omega )$, that conjugates $\mathcal {L}$ in (7.4) to constant coefficients, namely
  7.98 $$\begin{aligned} \mathcal {L}_{\infty }(\omega )&:=\Phi _{\infty } (\omega ) \circ \mathcal {L} \circ \Phi _{\infty }^{-1}(\omega )=\omega \cdot \partial _{\varphi }-\mathcal {D}_{\infty }(\omega ),\nonumber \\ \mathcal {D}_{\infty }(\omega )&:=\mathrm {diag}_{j\in S^c} \{ \mathrm {i} d_j^{\infty }(\omega ) \}. \end{aligned}$$
  The transformations $\Phi _{\infty }, \Phi _{\infty }^{-1}$ are tame and they satisfy for $s_0\le s\le \mathcal {S}$ (recall $\mu _1$ in Theorem 7.1)
  7.99 $$\begin{aligned} \Vert (\Phi ^{\pm 1}_{\infty }-\mathrm {I}) h \Vert ^{\gamma ^{3/2}, \Omega _{\infty }^{2 \tilde{\gamma }}}_{s}&\lesssim _s \big (\varepsilon ^{4-3a} \gamma ^{-3/2}+\varepsilon \gamma ^{-5/2} \Vert \mathfrak {I}_{\delta } \Vert _{s+\mu _1}^{\gamma , \mathcal {O}_0}\big ) \Vert h \Vert ^{\gamma ^{3/2}, \Omega _{\infty }^{2 \tilde{\gamma }}}_{s_0}\nonumber \\&\quad +\varepsilon ^{4-3a} \gamma ^{-3/2} \Vert h \Vert ^{\gamma ^{3/2}, \Omega _{\infty }^{\tilde{\gamma }}}_s. \end{aligned}$$
  Moreover $\Phi _{\infty }, \Phi _{\infty }^{-1}$ are symplectic, and $\mathcal {L}_{\infty }$ is a Hamiltonian operator.
(iii) **(Dependence on**$i_{\delta }(\omega )$) Let $i_1(\omega )$ and $i_2(\omega )$ be two Lipschitz maps satisfying (6.12) with $\mathfrak {I}_{\delta }\rightsquigarrow i_k(\varphi )-(\varphi , 0, 0)$, $k=1, 2$, and such that
  7.100 $$\begin{aligned} \Vert i_1-i_2 \Vert _{s_0+\mu _1}\lesssim \rho N^{-(\tau +1)} \end{aligned}$$
  for *N* sufficiently large and $0<\rho <\gamma ^{3/2}/4$. Fix ${\gamma }_1\in [\gamma ^{3/2}/2, 2 \gamma ^{3/2}]$ and ${\gamma }_2:={\gamma }_1-\rho$. Let $r_j^{(\infty )}(\omega , i_k(\omega ))$ be the sequence in (7.95) with $\tilde{{\gamma }}\rightsquigarrow {\gamma }_k$ for $k=1, 2$. Then for all $\omega \in \Omega _{\infty }^{{\gamma }_1}(i_1)$ we have, for some $\kappa >(3/2) \tau$,
  7.101 $$\begin{aligned} {\gamma }^{-1}|\Delta _{12} m |+ \sup _j \langle j\rangle |\Delta _{12}r_j^{(\infty )}| \le \varepsilon {\gamma }^{-1} \Vert i_1-i_2 \Vert _{s_0+\mu _1}+\varepsilon ^{4- 3 a} N^{-\kappa }. \end{aligned}$$

#### Proof

The proof of items (*i*)–(*ii*) follow by Theorem 1.9 in [33]. The only point left to prove is item (*iii*). We apply Theorems 1.4, 1.5 in [33]. We have that

7.102 $$\begin{aligned} {\gamma }^{-1}|m^{(N)}(i_2)-m(i_1) |+\langle j \rangle |r_j^{(N)}(i_2)-r_j^{(\infty )}(i_1) |\lesssim \varepsilon {\gamma }^{-1} \Vert i_1-i_2 \Vert _{s_0+\mu _1}+\varepsilon ^{4- 3 a} N^{-\kappa }, \end{aligned}$$

where $\kappa >\tau$. Here $m^{(N)}(i_2)$, $r_j^{(N)}(i_2)$ are defined in (1.39) of [33] and they are an approximation of $m(i_2)$ and $r_j^{(\infty )}(i_2)$ satisfying

7.103 $$\begin{aligned} {\gamma }^{-1}|m^{(N)}(i_2)-m(i_2) |+ \langle j \rangle |r_j^{(N)}(i_2)-r_j^{(\infty )}(i_2) |\lesssim \varepsilon ^{4- 3 a} N^{-\kappa }{.} \end{aligned}$$

The bounds (7.102), (7.103) imply the (7.101). $\quad \square$

### Proof of the inversion assumption (6.9)

We are in position to give estimates on the inverse of the operator $\mathcal {L}_{\omega }$ in (6.15). Let us now define (recall (5.3))

7.104 $$\begin{aligned} \mathcal {F}_{\infty }^{2\gamma }(i_{\delta }):=\{ \omega \in \mathcal {O}_0 : |\omega \cdot \ell -\,d_j^{\infty }(\omega ) |\ge \frac{2\gamma }{\langle \ell \rangle ^{\tau }} , \quad \forall \ell \in \mathbb {Z}^{\nu } , \forall j\in S^c\}. \end{aligned}$$

We deduce the inversion assumption (6.9) by the following result.

#### Proposition 7.14

Assume the hypothesis of Theorem 7.13, (6.12) with $\mathfrak {p}_1\ge \mu _1+2\tau +1$, where $\mu _1$ is given in Theorem 7.1. Then for all $\omega \in \Omega _{\infty }:=\Omega _{\infty }^{2 \gamma ^{3/2}}(i_{\delta }) \cap \mathcal {F}_{\infty }^{2\gamma }(i_{\delta })$ (see (7.97)), for any function $g\in H^{s+2\tau +1}_{S^{\perp }}(\mathbb {T}^{\nu +1})$ the equation $\mathcal {L}_{\omega } h=g$ has a solution $h=\mathcal {L}_{\omega }^{-1} g\in H_{S^{\perp }}^s(\mathbb {T}^{\nu +1})$, satisfying

7.105 $$\begin{aligned} \Vert \mathcal {L}_{\omega }^{-1} g \Vert _s^{\gamma , \Omega _{\infty }} \lesssim _s \gamma ^{-1} (\Vert g \Vert _{s+2\tau +1}^{\gamma , \Omega _{\infty }}+\varepsilon \gamma ^{-5/2}\Vert \mathfrak {I}_{\delta } \Vert _{s+\mathfrak {p}_1}^{\gamma , \Omega _{\infty }}\Vert g \Vert _{s_0}^{\gamma , \Omega _{\infty }}). \end{aligned}$$

#### Proof

We conjugated the operator $\mathcal {L}_{\omega }$ in (6.15) to a diagonal operator $\mathcal {L}_{\infty }=\chi \mathcal {L}_{\omega } \chi ^{-1}$, see (7.98), with (recall (7.99) and Theorem 7.1) $\chi :=\Phi _{\infty }\circ \Upsilon$. Moreover, by (7.9) and (7.99) we have the following estimates

$$\begin{aligned} \Vert \chi ^{\pm 1} h \Vert _s^{\gamma , \Omega _{\infty }}\lesssim _s \Vert h \Vert ^{\gamma , \Omega _{\infty }}_s+\varepsilon \gamma ^{-5/2}\Vert \mathfrak {I}_{\delta } \Vert _{s+\mu _1}^{\gamma , \mathcal {O}_0} \Vert h \Vert ^{\gamma , \Omega _{\infty }}_{s_0}. \end{aligned}$$

We have

7.106 $$\begin{aligned} \mathcal {L}_{\infty }^{-1} g=\sum _{j\ne 0} \frac{g_{\ell j}}{\mathrm {i}\big (\omega \cdot \ell -d_j^{\infty }(\omega )\big )}\,e^{\mathrm {i}(\ell \cdot \varphi +j x)} \end{aligned}$$

and then $\Vert \mathcal {L}_{\infty }^{-1} g \Vert ^{\gamma , \Omega _{\infty }}_s\le \gamma ^{-1} \Vert g \Vert _{s+2\tau +1}^{\gamma , \Omega _{\infty }}.$ Thus we get the estimate (7.105). $\quad \square$

## The Nash–Moser Nonlinear Iteration

In this section we prove Theorem 5.4. It will be a consequence of the Nash–Moser theorem 8.1.

Consider the finite-dimensional subspaces

$$\begin{aligned} E_n:=\{ \mathfrak {I}(\varphi )=(\Theta , y, z)(\varphi ) : \Theta =\Pi _n\Theta , y=\Pi _n y, z=\Pi _n z \} \end{aligned}$$

where

8.1 $$\begin{aligned} N_n:=N_0^{\chi ^n}{,} \quad n=0, 1, 2, \ldots {,} \quad \chi :=3/2{,} \quad N_0>0\, \end{aligned}$$

and $\Pi _n$ are the projectors (which, with a small abuse of notation, we denote with the same symbol)

8.2 $$\begin{aligned}&\Pi _n \Theta (\varphi ):=\sum _{|\ell |<N_n} \Theta _{\ell }\, e^{\mathrm {i} \ell \cdot \varphi }{,} \,\, \Pi _n y(\varphi ):=\sum _{|\ell |<N_n} y_{\ell }\, e^{\mathrm {i} \ell \cdot \varphi }{,}\nonumber \\&\qquad \,\,\text{ where }\,\, \Theta (\varphi )=\sum _{\ell \in \mathbb {Z}^{\nu }} \Theta _{\ell }\,e^{\mathrm {i} \ell \cdot \varphi }{,} \,\, y(\varphi )=\sum _{\ell \in \mathbb {Z}^{\nu }} y_{\ell } \, e^{\mathrm {i} \ell \cdot \varphi }{,}\nonumber \\&\Pi _n z(\varphi , x):=\sum _{|(\ell , j) |<N_n} z_{\ell j}\,e^{\mathrm {i}(\ell \cdot \varphi +j x)}{,} \,\,\text{ where }\quad z(\varphi , x)= \sum _{\ell \in \mathbb {Z}^{\nu }, j\in S^c} z_{\ell j}\,e^{\mathrm {i} (\ell \cdot \varphi +j x)}{.} \end{aligned}$$

We define $\Pi _n^{\perp }=\mathrm {I}-\Pi _n$. The classical smoothing properties hold, namely, for all $\alpha , s\ge 0$,

8.3 $$\begin{aligned}&\Vert \Pi _n \mathfrak {I} \Vert _{s+\alpha }^{{\gamma }, \mathcal {O}}\!\le \! N_n^{\alpha } \Vert \mathfrak {I}_{\delta } \Vert _s^{{\gamma }, \mathcal {O}}, \quad \forall \,\mathfrak {I}(\omega )\in H^s{,} \quad \Vert \Pi _n^{\perp } \mathfrak {I} \Vert _s^{{\gamma }, \mathcal {O}}\nonumber \\&\quad \le N_n^{-\alpha } \Vert \mathfrak {I} \Vert _{s+\alpha }^{{\gamma }, \mathcal {O}}{,} \quad \forall \, \mathfrak {I}(\omega )\!\in \! H^{s+\alpha }{.} \end{aligned}$$

Recall (5.3), (4.8) for the definition of *b* we set $a:=2b-2$. We define the following constants

8.4 $$\begin{aligned} \alpha _0&:=3\mu +3{,} \qquad \qquad \qquad \alpha :=3\alpha _0+1{,} \qquad \qquad \qquad \alpha _1:=(\alpha -3 \mu )/2{,}\nonumber \\ k&:=3(\alpha _0+\rho ^{-1})+1{,} \qquad \beta _1:=6\alpha _0+3 \rho ^{-1} +3{,}\nonumber \\&\frac{1}{2}\left( \frac{1-(9/2)a}{C_1 (1+a)}\right)<\rho <\frac{1-(9/2)a}{C_1 (1+a)}\, \end{aligned}$$

where $\mu :=\mu ( \nu )>0$ is the “loss of regularity” given by the Theorem 6.1 and $C_1$ is fixed below.

### Theorem 8.1

(Nash–Moser). Assume that $f\in C^{\infty }$ (see (1.3)). Let $\tau :=2\nu +6$. Then there exist $C_1>\max \{ \alpha _0+\alpha , C_0 \}$ (where $C_0:=C_0( \nu )$ is the one in Theorem 7.13), $\delta _0:=\delta _0( \nu )>0$ such that, if

8.5 $$\begin{aligned} N_0^{C_1} \varepsilon ^{b_*+1} \gamma ^{-7/2}<\delta _0{,} \quad \gamma :=\varepsilon ^{2+a}=\varepsilon ^{2 b}{,} \quad N_0:=(\varepsilon \gamma ^{-1})^{\rho }{,} \quad b_*=9- 2 b{,} \end{aligned}$$

then there exists $C_{*}=C_{*}(S)>0$ such that for all $n\ge 0$ the following holds:

$$(\mathcal {P}1)_n$$
there exists a function $(\mathfrak {I}_n, \zeta _n):\mathcal {G}_n \subseteq \Omega _{\varepsilon } \rightarrow E_{n-1}\times \mathbb {R}^{\nu }, \omega \mapsto (\mathfrak {I}_n(\omega ), \zeta _n(\omega )), (\mathfrak {I}_0, \zeta _0):=(0, 0), E_{-1}:=\{0\}$, where the set $\mathcal {G}_0$ is defined in (5.7) and the sets $\mathcal {G}_n$ for $n\ge 1$ are defined inductively by:
  8.6 $$\begin{aligned}&\mathcal {G}_{n+1}:= \bigcap _{i=0}^2 \Lambda _{n+1}^{(i)}{,}\;\;\mathrm{with}\;\;\; \Lambda ^{(0)}_{n+1}:=\left\{ \omega \in \mathcal {G}_n : |\omega \cdot \ell +m(i_n) j |\ge \frac{2\,\gamma _n}{\langle \ell \rangle ^{\tau }}, \,\,\forall j\in S^c , \ell \in \mathbb {Z}^{\nu } \right\} {,} \nonumber \\&\Lambda ^{(1)}_{n+1}:=\left\{ \omega \in \mathcal {G}_n : |\omega \cdot \ell +d_j^{\infty }(i_n) |\ge \frac{2\,\gamma _n}{\langle \ell \rangle ^{\tau }}, \,\, \forall j\in S^c , \ell \in \mathbb {Z}^{\nu } \right\} {,}\nonumber \\&\Lambda ^{(2)}_{n+1}:=\left\{ \omega \in \mathcal {G}_n : |\omega \cdot \ell +d_j^{\infty }(i_n)-d_k^{\infty }(i_n) |\ge \frac{2\,\gamma ^{3/2}_n\,}{\langle \ell \rangle ^{\tau }}, \,\, \forall j, k\in S^c, j\ne k , \ell \in \mathbb {Z}^{\nu } \right\} {,} \end{aligned}$$
  where $\gamma _n:=\gamma (1+2^{-n})$, $\gamma ^{*}_n:=\gamma ^{3/2}(1+2^{-n})$ and $d_j^{\infty }(\omega ):=d_j^{\infty }(\omega , i_n(\omega ))$ are defined in (7.95) (and $d_0^{\infty }(\omega )=0$). Moreover $|\zeta _n |^{\gamma , \mathcal {G}_n} \lesssim \Vert \mathcal {F}(U_n) \Vert _{s_0}^{\gamma , \mathcal {G}_n}$ and
  8.7 $$\begin{aligned} \Vert \mathfrak {I}_n \Vert _{s_0+\mu }^{\gamma , \mathcal {G}_n} \le C_* \varepsilon ^{b_*} \gamma ^{-1}{,} \quad \Vert \mathcal {F}(U_n) \Vert _{s_0+\mu +3}^{\gamma , \mathcal {G}_n} \le C_* \varepsilon ^{b_*}{,} \end{aligned}$$
  where $U_n:=(i_n, \zeta _n)$ with $i_n(\varphi )=(\varphi , 0, 0)+\mathfrak {I}_n(\varphi )$. The differences $\widehat{\mathfrak {I}}_n:=\mathfrak {I}_n-\mathfrak {I}_{n-1}$ (where we set $\widehat{\mathfrak {I}}_0:=0$) is defined on $\mathcal {G}_n$, and satisfy
  8.8 $$\begin{aligned} \Vert \widehat{\mathfrak {I}}_1 \Vert _{s_0+\mu }^{\gamma , \mathcal {G}_1} \le C_* \varepsilon ^{b_*} \gamma ^{-1}{,} \quad \Vert \widehat{\mathfrak {I}}_n \Vert _{s_0+\mu }^{\gamma , \mathcal {G}_{n}} \le C_* \varepsilon ^{b_*} \gamma ^{-1} N_{n-1}^{-\alpha }{,} \quad \forall n\ge 2{.} \end{aligned}$$
$$(\mathcal {P} 2)_n$$
$\Vert \mathcal {F}(U_n) \Vert _{s_0}^{\gamma , \mathcal {G}_n} \le C_* \varepsilon ^{b_*} N_{n-1}^{-\alpha }$ where we set $N_{-1}:=1$.
$$(\mathcal {P}3)_n$$
(High Norms) $\Vert \mathfrak {I}_n \Vert _{s_0+\beta _1}^{\gamma , \mathcal {G}_n} \le C_* \varepsilon ^{b_*} \gamma ^{-1} N_{n-1}^k$ and $\Vert \mathcal {F}(U_n) \Vert _{s_0+\beta _1}^{\gamma , \mathcal {G}_n} \le C_* \varepsilon ^{b*} N_{n-1}^k$.
$$(\mathcal {P} 4)_n$$
(Measure) The measure of the “Cantor-like” sets $\mathcal {G}_n$ satisfies
  8.9 $$\begin{aligned} |\Omega _{\varepsilon }{\setminus } \mathcal {G}_0 |\le C_* \varepsilon ^{2 (\nu -1)} \gamma {,} \quad |\mathcal {G}_n {\setminus } \mathcal {G}_{n+1} |\le C_* \varepsilon ^{2 (\nu -1)} \gamma N_{n-1}^{-1}{.} \end{aligned}$$

### Proof

To simplify the notations we omit the index ${\gamma }, \mathcal {G}_n$ on the norm $\Vert \cdot \Vert _s$.

*Proof of*$(\mathcal {P}_1)_0, (\mathcal {P}_2)_0, (\mathcal {P}_3)_0$. Recalling (5.10), we have, by the second estimate in (5.13),

$$\begin{aligned} \Vert \mathcal {F}(U_0)\Vert _s =\Vert \mathcal {F}((\varphi , 0, 0), 0) \Vert _s =\Vert X_P(\varphi , 0, 0) \Vert _s\lesssim _s \varepsilon ^{9-2 b}{.} \end{aligned}$$

Hence the smallness conditions in $(\mathcal {P}_1)_0, (\mathcal {P}_2)_0, (\mathcal {P}_3)_0$ hold taking $C_*$ large enough.

*Assume that*$(\mathcal {P}_1)_n, (\mathcal {P}_2)_n, (\mathcal {P}_3)_n$*hold for some*$n\ge 0$, *and prove*$(\mathcal {P}_1)_{n+1}, (\mathcal {P}_2)_{n+1}, (\mathcal {P}_3)_{n+1}$. By (8.4) and (8.5)

$$\begin{aligned} N_0^{C_1} \varepsilon ^{b_*+1} \gamma ^{-7/2} =\varepsilon ^{1-(9/2) a-\rho \,C_1 (1+a)}<\delta _0 \end{aligned}$$

for $\varepsilon$ small enough. If we take $C_1$ bigger than $C_0$ in Theorem 7.13 then (7.94) holds. In (6.12) we consider $\mathfrak {p}_0=\mathfrak {p}_1+\tilde{\mathfrak {p}}$, where $\mathfrak {p}_1:=\mu _1+2\tau +1$ and $\tilde{\mathfrak {p}}$ appears in (6.5). Since $\mu \gg \mathfrak {p}_0$ (8.7) implies (6.3) and so (6.12), and Proposition 7.14 applies. Hence the operator $\mathcal {L}_{\omega }:=\mathcal {L}_{\omega }(\omega , i_n (\omega ))$ in (6.15) is defined on $\mathcal {O}_0= \mathcal {G}_n$ and is invertible for all $\omega \in \mathcal {G}_{n+1}$ since $\mathcal {G}_{n+1}\subseteq \Omega ^{2\gamma ^{*}_n}_{\infty }(i_n) \cap \mathcal {F}_{\infty }^{2{\gamma }_n}(i_n)$ and the (7.105) holds. This means that the assumption (6.9) of Theorem 6.1 is verified with $\Omega _{\infty }=\mathcal {G}_{n+1}$. By Theorem 6.1 there exists an approximate inverse $\mathbf{T} _n(\omega ):=\mathbf{T} _0(\omega , i_n(\omega ))$ of the linearized operator $L_n(\omega ):=\mathtt {D}\mathcal {F}(\omega , i_n(\omega ),\zeta _n) \equiv \mathtt {D}\mathcal {F}(\omega , i_n(\omega ),0)$, satisfying (6.10). By (8.5), (8.7)

8.10 $$\begin{aligned}&\Vert \mathbf{T} _n g\Vert _s\lesssim _s \gamma ^{-1} (\Vert g \Vert _{s+\mu } +\varepsilon \gamma ^{-5/2} \{ \Vert \mathfrak {I}_n \Vert _{s+\mu } +\gamma ^{-1} \Vert \mathfrak {I}_n \Vert _{s_0+\mu } \Vert \mathcal {F}(U_n) \Vert _{s+\mu } \} \Vert g \Vert _{s_0+\mu }){,} \end{aligned}$$

8.11 $$\begin{aligned}&\Vert \mathbf{T} _n g \Vert _{s_0}\lesssim \gamma ^{-1} \Vert g \Vert _{s_0+\mu } \end{aligned}$$

and, by (6.11), using also (8.5), (8.7), (8.3),

8.12 $$\begin{aligned} \Vert (L_n\circ \mathbf{T} _n-\mathrm {I}) g \Vert _s \lesssim _s&\varepsilon ^{2 b-1}\gamma ^{-2} (\Vert \mathcal {F}(U_n) \Vert _{s_0+\mu }\Vert g \Vert _{s+\mu } +\Vert \mathcal {F}(U_n) \Vert _{s+\mu }\Vert g \Vert _{s_0+\mu }\nonumber \\&+ \varepsilon \gamma ^{-5/2}\Vert \mathfrak {I}_n \Vert _{s+\mu } \Vert \mathcal {F}(U_n) \Vert _{s_0+\mu }\Vert g \Vert _{s_0+\mu }) \end{aligned}$$

8.13 $$\begin{aligned} \Vert (L_n\circ \mathbf{T} _n-\mathrm {I}) g \Vert _{s_0} \lesssim&\varepsilon ^{2 b-1}\gamma ^{-2} (\Vert \Pi _n \mathcal {F}(U_n) \Vert _{s_0+\mu } +\Vert \Pi _n^{\perp } \mathcal {F}(U_n) \Vert _{s_0+\mu }) \Vert g \Vert _{s_0+\mu }\end{aligned}$$

8.14 $$\begin{aligned} \lesssim&\varepsilon ^{2 b-1}\gamma ^{-2} N_n^{\mu } \Big ( \Vert \mathcal {F}(U_n) \Vert _{s_0}+N_n^{-\beta _1} \Vert \mathcal {F}(U_n) \Vert _{s_0+\beta _1} \Big ) \Vert g \Vert _{s_0+\mu }{.} \end{aligned}$$

Now, for all $\omega \in \mathcal {G}_{n+1}$, we can define, for $n\ge 0$,

8.15 $$\begin{aligned} U_{n+1}:=U_n+H_{n+1}, \quad H_{n+1} :=(\widehat{\mathfrak {I}}_{n+1}, \widehat{\zeta }_{n+1}) :=-\tilde{\Pi }_n \mathbf{T} _n \Pi _n \mathcal {F}(U_n)\in E_n\times \mathbb {R}^{\nu }{,} \end{aligned}$$

where $\tilde{\Pi }_n(\mathfrak {I}, \zeta ):=(\Pi _n \mathfrak {I}, \zeta )$ with $\Pi _n$ defined in (8.2). By construction we have

$$\begin{aligned}&\mathcal {F}(U_{n+1})=\mathcal {F}(U_n)+L_n H_{n+1}+Q_n{,}\\&\quad Q_n:=Q(U_n, H_{n+1}){,} \quad Q(U_n, H):=\mathcal {F}(U_n+H)-\mathcal {F}(U_n)-L_n H{,} \quad H\in E_n\times \mathbb {R}^{\nu }{.} \end{aligned}$$

Then, by the definition of $H_{n+1}$ in (8.15), using $[L_n, \Pi _n]$ and writing $\tilde{\Pi }_n^{\perp }(\mathfrak {I}, \zeta ):=(\Pi _n^{\perp } \mathfrak {I}, 0)$ we have

$$\begin{aligned} \mathcal {F}(U_{n+1})&= \mathcal {F}(U_n)-L_n \tilde{\Pi }_n \mathbf{T} _n \Pi _n \mathcal {F}(U_n)+Q_n =\mathcal {F}(U_n)-L_n \mathbf{T} _n \Pi _n \mathcal {F}(U_n)\\&\quad +L_n \tilde{\Pi }_n^{\perp } \mathbf{T} _n \Pi _n \mathcal {F}(U_n)+Q_n\\&=\mathcal {F}(U_n)-\Pi _n L_n \mathbf{T} _n \Pi _n \mathcal {F}(U_n) +(L_n \tilde{\Pi }_n^{\perp }-\Pi _n^{\perp }L_n) \mathbf{T} _n \Pi _n \mathcal {F}(U_n)+Q_n\\&=\Pi _n^{\perp } \mathcal {F}(U_n)+R_n+Q_n+Q'_n \end{aligned}$$

where

8.16 $$\begin{aligned} R_n:=(L_n \tilde{\Pi }_n^{\perp }-\Pi _n^{\perp }L_n) \mathbf{T} _n \Pi _n \mathcal {F}(U_n){,} \quad Q'_n:=-\Pi _n (L_n \mathbf{T} _n-\mathrm {I}) \Pi _n \mathcal {F}(U_n){.} \end{aligned}$$

### Lemma 8.2

Define

8.17 $$\begin{aligned} w_n:=\varepsilon \gamma ^{-2} \Vert \mathcal {F}(U_n) \Vert _{s_0}{,} \qquad B_n:=\varepsilon \gamma ^{-1} \Vert \mathfrak {I}_n \Vert _{s_0+\beta _1} +\varepsilon \gamma ^{-2} \Vert \mathcal {F}(U_n) \Vert _{s_0+\beta _1}{.} \end{aligned}$$

Then there exists $K:=K(s_0, \beta _1)>0$ such that, for all $n \ge 0$, setting $\alpha _0:=3\mu +3$

8.18 $$\begin{aligned} w_{n+1}\le K N_n^{\alpha _0+\rho ^{-1}-\beta _1} B_n +K N_{n}^{\alpha _0} w_n^2{,} \qquad B_{n+1}\le K N_n^{\alpha _0+\rho ^{-1}} B_n{.} \end{aligned}$$

The proof of Lemma 8.2 follows almost word by word the proof of Lemma 9.2 in [4].

*Proof of*$(\mathcal {P}_3)_{n+1}$. By (8.18) and $(\mathcal {P}_3)_n$

8.19 $$\begin{aligned} B_{n+1}\le K N_n^{\alpha _0+\rho ^{-1}} B_n \le 2 C_* K \varepsilon ^{b_*+1} \gamma ^{-2} N_n^{\alpha _0+\rho ^{-1}} N_{n-1}^k\le C_* \varepsilon ^{b_*+1} \gamma ^{-2} N_n^k{,} \end{aligned}$$

provided $2 K N_n^{\alpha _0+\rho ^{-1}-k}N_{n-1}^k\le 1, \forall n\ge 0$. Choosing *k* as in (8.4) and $N_0$ large enough, i.e. for $\varepsilon$ small enough. By (8.17) and the bound (8.19) $(\mathcal {P}_3)_{n+1}$ holds.

*Proof of*$(\mathcal {P}_2)_{n+1}$. Using (8.17), (8.18) and $(\mathcal {P}_2)_n, (\mathcal {P}_3)_n$, we get

$$\begin{aligned}&w_{n+1}\le K N_n^{\alpha _0+\rho ^{-1}-\beta _1} B_n +K N_n^{\alpha _0} w_n^2\le K N_n^{\alpha _0+\rho ^{-1}-\beta _1} 2 C_* \varepsilon ^{b_*+1} \gamma ^{-2} N_{n-1}^k\\&+K N_n^{\alpha _0} (C_*\varepsilon ^{b_*+1}\gamma ^{-2} N_{n-1}^{-\alpha })^2 \end{aligned}$$

and $w_{n+1}\le C_* \varepsilon ^{b_*+1} \gamma ^{-2} N_n^{-\alpha }$ provided that

8.20 $$\begin{aligned} 4 K N_n^{\alpha _0+\rho ^{-1}-\beta _1+\alpha } N_{n-1}^k\le 1{,} \quad 2 K C_* \varepsilon ^{b_*+1} \gamma ^{-2}N_n^{\alpha _0+\alpha } N_{n-1}^{-2 \alpha }\le 1{,} \,\, \forall n\ge 0{.} \end{aligned}$$

The inequalities in (8.20) hold by (8.5), taking $\alpha$ as in (8.4), $C_1>\alpha _0+\alpha$ and $\delta _0$ in (8.5) small enough. By (8.17), the inequality $w_{n+1}\le C_* \varepsilon ^{b_*+1} \gamma ^{-2} N_n^{-\alpha }$ implies $(\mathcal {P}_2)_{n+1}$.

*Proof of*$(\mathcal {P}_1)_{n+1}$. The bound (8.8) for $\widehat{\mathfrak {I}}_1$ follows by (8.15), (8.10) (for $s=s_0+\mu$) and $\Vert \mathcal {F}(U_0) \Vert _{s_0+2 \mu } \lesssim _{s_0+2\mu } \varepsilon ^{b_*}{.}$ The bound (8.8) for $\widehat{\mathfrak {I}}_{n+1}$ follows by (8.2), $(\mathcal {P}_2)_n$ and (8.4). It remains to prove that (8.7) holds at the step $n+1$. We have

8.21 $$\begin{aligned} \Vert \mathfrak {I}_{n+1} \Vert _{s_0+\mu } \le \sum _{k=1}^{n+1} \Vert \widehat{\mathfrak {I}}_k \Vert _{s_0+\mu } \le C_* \varepsilon ^{b_*} \gamma ^{-1} \sum _{k \ge 1} N_{k-1}^{-\alpha _1}\lesssim C_* \varepsilon ^{b_*} \gamma ^{-1} \end{aligned}$$

taking $\alpha _1$ as in (8.4) and $N_0$ large enough, i.e. $\varepsilon$ small enough. Moreover, using (8.2), $(\mathcal {P}_2)_{n+1}, (\mathcal {P}_3)_{n+1}$, (8.4) we get

$$\begin{aligned} \Vert \mathcal {F}(U_{n+1}) \Vert _{s_0+\mu +1}&\le N_n^{\mu +1} \Vert \mathcal {F}(U_{n+1}) \Vert _{s_0} +N_n^{\mu +1-\beta _1}\Vert \mathcal {F}(U_{n+1}) \Vert _{s_0+\beta _1}\\&\le C_* \varepsilon ^{b_*} N_n^{\mu +1-\alpha } +C_* \varepsilon ^{b_*}N_n^{\mu +1-\beta _1+k}\lesssim C_* \varepsilon ^{b_*}{,} \end{aligned}$$

which is the second inequality in (8.7) at the step $n+1$. The bound $|\zeta _{n+1} |^{\gamma }\lesssim \Vert \mathcal {F}(U_{n+1}) \Vert _{s_0}^{\gamma }$ is a consequence of Lemma 6.1 in [39].

To conclude the proof of Theorem (8.1) it remains to show the bounds (8.9). This is done in the next section. $\quad \square$

### Measure estimates

Let us define for $0<\eta \le \overline{\jmath }_1^4\,\sqrt{\varepsilon }$, $\sigma \ge 1$ and $n\in \mathbb {N}$

8.22 $$\begin{aligned} R_{\ell j k}(\eta , \sigma ):=R_{\ell j k}(i_{n},\eta , \sigma )&:=\{ \omega \in \mathcal {G}_n : |\omega \cdot \ell +d_j^{\infty }-d_k^{\infty } |\le {2\eta }\langle \ell \rangle ^{-\sigma }\}{,} \end{aligned}$$

8.23 $$\begin{aligned} Q_{\ell j }(\eta , \sigma ):=Q_{\ell j }(i_n,\eta , \sigma )&:=\{ \omega \in \mathcal {G}_n : |\omega \cdot \ell +m j |\le {2\eta }\langle \ell \rangle ^{-\sigma }\}{,}\end{aligned}$$

8.24 $$\begin{aligned} P_{\ell j }(\eta , \sigma ):=P_{\ell j }(i_n,\eta , \sigma )&:=\{ \omega \in \mathcal {G}_n : |\omega \cdot \ell +d_j^{\infty } |\le {2\eta }\langle \ell \rangle ^{-\sigma }\}{.} \end{aligned}$$

Recalling (8.6) we can write, setting $\eta \rightsquigarrow \gamma _n$ for the sets $Q_{\ell j }(\eta ,\sigma )$ and $P_{\ell j }(\eta ,\sigma )$, $\eta \rightsquigarrow \gamma ^{*}_n$ for the set $R_{\ell jk}(\eta ,\sigma )$, and $\sigma \rightsquigarrow \tau$,

8.25 $$\begin{aligned} \mathcal {G}_n {\setminus } \mathcal {G}_{n+1}= \bigcup _{\ell \in \mathbb {Z}^{\nu }, j, k\in S^c} \Big ( R_{\ell j k}(i_n,\gamma _{n}^{*},\tau ) \cup Q_{\ell j }(i_n,\gamma _{n},\tau ) \cup P_{\ell j}(i_n,\gamma _{n},\tau )\Big ){.} \end{aligned}$$

Since, by (5.7) and $\gamma >\gamma ^{3/2}$ (see (8.5)), $R_{\ell j k}(i_n)=\emptyset$ for $j=k$, in the sequel we assume that $j\ne k$. We start with a preliminary lemma, which gives a first relation between $\ell ,j,k$ which must be satisfied in order to have non empty resonant sets.

#### Lemma 8.3

Let $n \ge 0$. There is a constant $C>0$ dependent of the tangential set and independent of $\ell , j, k, n, i_n, \omega$ such that the following holds:

- If $$R_{\ell j k}(i_n,\eta ,\sigma )\ne \emptyset $$ then $$|\ell |\ge C |{\lambda }(j)-{\lambda }(k) |\ge \frac{C}{2}\,|j-k |$$;
- If $$Q_{\ell j}(i_n,\eta ,\sigma )\ne \emptyset $$ then $$|\ell |\ge C |j |$$;
- If $$P_{\ell j}(i_n,\eta ,\sigma )\ne \emptyset $$ then $$|\ell |\ge C |j |$$.

#### Proof

If $R_{\ell j k}(i_n)\ne \emptyset$, then there exists $\omega$ such that

$$\begin{aligned} |d_j^{\infty }(\omega , i_n(\omega ))-d_k^{\infty }(\omega , i_n(\omega )) |< {2 \eta }{\langle \ell \rangle ^{-\sigma }} + |{\omega }\cdot \ell |{.} \end{aligned}$$

Moreover, using (7.95), (7.96), (7.2), (7.5), we get $|d_j^{\infty }(\omega , i_n(\omega ))-d_k^{\infty }(\omega , i_n(\omega )) |\ge \frac{1}{3} |{\lambda }(j)-{\lambda }(k) |.$ Thus, for $\varepsilon$ small enough

$$\begin{aligned} 2 |\overline{\omega } ||\ell |\ge |{\omega }\cdot \ell |\ge \left( \frac{1}{3}-\frac{2 \eta }{\langle \ell \rangle ^{\sigma } |{\lambda }(j)-{\lambda }(k) |} \right) |{\lambda }(j) -{\lambda }(k) |\ge \frac{1}{4}|{\lambda }(j)-{\lambda }(k) |\end{aligned}$$

and this proves the first claim on $R_{\ell j k}$. If $Q_{\ell j}\ne \emptyset$ then we have $|m j|< {2 \eta }{\langle \ell \rangle ^{-\sigma }} + |{\omega }\cdot \ell |$. Hence, for $\varepsilon$ small enough, we have

$$\begin{aligned} |j |\le \frac{|\omega \cdot \ell |}{|m |} \le \frac{1}{\tilde{C}} |\ell |, \qquad \tilde{C}:=\frac{|m |}{4 |\overline{\omega } |}. \end{aligned}$$

Following the same arguments and by using that $|d_j |\ge C |j |$ for some constant $C>0$ we get the last statement. $\quad \square$

#### Measure of a resonant set

The aim of this subsection is to prove the following lemma.

##### Lemma 8.4

There is $\mathtt {r}_0>0$ such that, for any $0<\mathtt {r}\le \mathtt {r}_0$, and any choice of $S^{+}\in \mathcal {V}(\mathtt {r})$ we have that

8.26 $$\begin{aligned} |R_{\ell j k}(\eta , \sigma ) |\le K \varepsilon ^{2(\nu -1)} \eta \langle \ell \rangle ^{-\sigma }{,} \end{aligned}$$

for some $K=K(S)$. The same holds for $Q_{\ell j}(\eta ,\sigma )$ and $P_{\ell j}(\eta ,s)$.

The proof of the lemma above involves many arguments and we split it into several steps.

In several bounds we will evidence the dependence of the constants on the tangential set *S* in order to highlight that the smallness of the amplitudes $\xi$ depends on the choice of the tangential sites.

Let us first consider the set $R_{\ell j k}$, which is the most difficult case. We study the sub-levels of the function $\omega \mapsto \phi _R(\omega )$ defined by (recall (4.5),(7.95))

8.27 $$\begin{aligned} \phi _R(\omega )&:=\mathrm {i} \omega \cdot \ell +d_j^{\infty }(\omega )-d_k^{\infty }(\omega ) =\mathrm {i} \omega \cdot \ell +\mathrm {i} m(\omega ) ({\lambda }(j)-{\lambda }(k))\nonumber \\&\quad + \mathrm {i} \varepsilon ^2(\kappa _j-\kappa _k)(\omega ) +(r_j^{\infty }-r_k^{\infty })(\omega ). \end{aligned}$$

We recall that (see (7.2), (7.48))

8.28 $$\begin{aligned}&m=1+\varepsilon ^2 c(\omega )+\mathtt {r}_{m}(\omega ), \quad c(\omega )=\vec {v}\cdot \xi (\omega ){,} \quad \vec {v}:=(2/3)(1+\overline{\jmath }_k^2)_{k=1}^{\nu }\in \mathbb {R}^{\nu },\nonumber \\&\quad \kappa _j(\omega )=\vec {w}_j \cdot \xi (\omega ){,} \end{aligned}$$

where $\kappa _{j}$ is defined in (7.5) (see also (7.40)) and

8.29 $$\begin{aligned} \mathtt {r}_{m}:=\varepsilon ^4 d(\omega )+O(\varepsilon ^{10}\gamma ^{-2}){,} \quad |\mathtt {r}_{m}|^{\gamma }\lesssim \overline{\jmath }_1 \varepsilon ^4{,} \quad |\nabla _{\omega } \mathtt {r}_{m}|\lesssim \overline{\jmath }_1\varepsilon ^2+O(\varepsilon ^{10} \gamma ^{-3}){.} \end{aligned}$$

We first study some properties of the function $\phi _{R}(\omega )$ in (8.27).

**The small divisor**$\phi _{R}(\omega )$**as an affine function of**$\omega$ It will be useful to consider $\phi _R(\omega )$ in (8.27) as a small perturbation of an affine function in $\omega$

8.30 $$\begin{aligned} \phi _R(\omega ) =a_{j k}+b_{\ell j k}\cdot \omega +q_{j k}(\omega ){,} \qquad \ell \in \mathbb {Z}^{\nu },\, j, k\in S^c{.} \end{aligned}$$

where

8.31 $$\begin{aligned} a_{j k}&:=\mathrm {i} \Big ( ({\lambda }(j)-{\lambda }(k)) [1- \vec {v}\cdot \mathbb {A}^{-1}\overline{\omega }] +(\vec {w}_k-\vec {w}_j) \cdot \mathbb {A}^{-1}\overline{\omega }\Big ){,} \end{aligned}$$

8.32 $$\begin{aligned} b_{l j k}&:=\mathrm {i} \Big (\ell + ({\lambda }(j)-{\lambda }(k)) \mathbb {A}^{-T} \vec {v} +\mathbb {A}^{-T}(\vec {w}_j-\vec {w}_k)\Big ){,} \end{aligned}$$

and the remainder $q_{j k}(\omega )$ satisfies

8.33 $$\begin{aligned} |q_{j k}(\omega ) |^{sup}&\lesssim \overline{\jmath }_1 \varepsilon ^{4}|j-k|+\varepsilon ^{4-3a}{,}\nonumber \\ |q_{j k}(\omega ) |^{lip}&\le |\mathtt {r}_{m}(\omega ) |^{lip}|{\lambda }(j)-{\lambda }(k)|+|r_j^{\infty }-r_k^{\infty }|^{lip}\lesssim \overline{\jmath }_1 \varepsilon ^{2}|j-k|+\varepsilon ^{1- 4a}{.} \end{aligned}$$

##### Lemma 8.5

Denoting $\vec {p}_j = {\lambda }(j)\vec {v}+ \vec {w}_j$, we have the following bounds:

$$\begin{aligned}&|\vec {p}_j |\lesssim \overline{\jmath }_1^4|j|{,} \qquad |\vec {w}_j |\lesssim \overline{\jmath }_1^6|j|^{-1}{,}\qquad |\vec {p}_j- \vec {p}_k |\lesssim \overline{\jmath }_1^4 |j-k |{,} \\&|\vec {w}_j- \vec {w}_k |\lesssim {\overline{\jmath }_1^8} |j-k |(|j|^{-2}+|jk|^{-1}){.} \end{aligned}$$

##### Proof

The first bound follows by the fact that $3+\overline{\jmath }_i^2+j^2 \pm j_2 \overline{\jmath }_i\ge 3+\frac{j^2+\overline{\jmath }_i^2}{2} \ge \frac{j^2}{2}$ and $(1+j^2)(1+\overline{\jmath }_i^2)(2+j^2 +\overline{\jmath }_i^2)\lesssim \overline{\jmath }_1^4 \,j^4$. The others follow similarly. $\quad \square$

Fix $\alpha \in (0,1/2)$ and let

8.34 $$\begin{aligned} 0<\beta <{(2-\alpha )}{(\sigma +1)^{-1}}. \end{aligned}$$

We have the following estimates for sets $R_{\ell j k}$ with $|\ell |$ “large”.

##### Lemma 8.6

Let $|\ell |> \varepsilon ^{-\beta }$. Then $R_{\ell jk}(\eta ,\sigma )$ satisfies (8.26).

##### Proof

In this proof we shall denote by *C*(*S*) a running positive constant depending on the set *S*. Suppose that $|j-k |\le c_0 |\ell |$ with $c_0=c_0(S)$ small. By Lemmata 8.5 and 4.1 we have $|\mathbb {A}^{-T}(\vec {p}_j -\vec {p}_k)|\lesssim \overline{\jmath }_1^4 |j-k |< {|\ell |}/{2}$ for $c_0$ sufficiently small. This means that $|b_{\ell jk}| \gtrsim |\ell |/2$. Now suppose that $|j-k |> c_0 |\ell |$. Then

$$\begin{aligned} |a_{j k}|&\ge |{\lambda }(j)-{\lambda }(k) |\Big ( |1-\mathbb {A}^{-1}\overline{\omega } \cdot \vec {v}|-\frac{\mathbb {A}^{-T} (\vec {w}_j-\vec {w}_k)}{|{\lambda }(j)-{\lambda }(k) |} |\Big )\\&{\mathop {\ge }\limits ^{(\hbox {A.4}),(\hbox {A.7})}} |j-k |\Big (\frac{1}{2}- \frac{C(S)|j-k |}{c_0\,|\ell |\,|j k |} \Big ) \ge |j-k |\Big (\frac{1}{2}- 2\,c_0^{-1}\,C(S) \varepsilon ^{\beta } \Big ) \ge |j-k |/4 \end{aligned}$$

for $\varepsilon$ small enough. By (8.22), (8.33)

$$\begin{aligned}&2|b_{\ell j k} ||\overline{\omega } |\ge |b_{\ell j k} \cdot \omega |\ge |a_{j k} |-|\phi _{\ell j k}(\omega ) |-|q_{j k}(\omega ) |\\&\quad \ge \Big (\frac{1}{4}-\frac{2\eta }{c_0 \langle \ell \rangle ^{\sigma +1}} -C(S) \varepsilon ^4-\frac{\varepsilon ^{4-3 a}}{|j-k |} \Big ) |j-k |\ge \frac{1}{8} |j -k|, \end{aligned}$$

for $\varepsilon$ small enough and $\sigma \ge 1$. Again we have shown that $|b_{\ell jk}|>\delta |\ell |$ with $\delta := c_0/ 2|\overline{\omega } |$. Split $\omega =s \widehat{b}+b^\perp$ where $\widehat{b}:=b/|b |$ and $b^\perp \cdot b=0$. Let $\Psi _R(s):=\phi _R(s \widehat{b}+b^\perp )$. For $\varepsilon$ small enough, by (8.33), we get

$$\begin{aligned}&|\Psi _R(s_1)-\Psi _R(p) |\ge (|b |-|q_{j k} |^{lip}) |s_1-p |\\&\quad \ge \left( \delta -C(S)\varepsilon ^2-\frac{\varepsilon ^{1-4 a}}{|j-k |}\right) \, |j -k|\, |s_1-p |\ge \frac{\delta _1}{2} |j -k|\,|s_1-p |. \end{aligned}$$

The lemma follows by Fubini’s theorem. $\quad \square$

We now prove that if the main term (in size) of $\phi _{R}(\overline{\omega })$ is big enough and $|\ell |$ is bounded by some constant then the bad set $R_{\ell j k}(\eta , \sigma )=\emptyset$. We remark that

$$\begin{aligned} \phi _{R}(\overline{\omega }) - q_{jk}(\overline{\omega }) = a_{jk} +b_{\ell jk}\cdot \overline{\omega } = \overline{\omega }\cdot \ell +{\lambda }(j)-{\lambda }(k){.} \end{aligned}$$

##### Lemma 8.7

If $|\ell |\le \varepsilon ^{-\beta }$ and $|\overline{\omega }\cdot \ell +{\lambda }(j)-{\lambda }(k) |\ge \gamma _0 \langle \ell \rangle ^{-\sigma }$ , where $\gamma _0=\varepsilon ^{\alpha }$, then $R_{\ell j k}(\eta , \sigma )=\emptyset$.

##### Proof

By definition

$$\begin{aligned} |\omega \cdot \ell +d_j^{\infty }-d_k^{\infty } |\ge \gamma _0 \langle \ell \rangle ^{-\sigma } -|b_{\ell jk}||\omega -\overline{\omega }|-2 |q_{jk}|^{sup}. \end{aligned}$$

By Lemma 8.3 (recall (8.27)) we have $|j-k |\lesssim C |\ell |$ and so

8.35 $$\begin{aligned}&|b_{\ell jk}||\omega -\overline{\omega }|+2 |q_{jk}|^{sup} \le C(S)\varepsilon ^2 (|\ell |+ |j-k|) \lesssim C(S)\varepsilon ^2 |\ell | \lesssim C(S)\varepsilon ^{2-\beta }\nonumber \\&\quad \le \varepsilon ^{\alpha +\sigma \beta }/2 \le \frac{\gamma _0}{2\langle \ell \rangle ^{\sigma }} \end{aligned}$$

for some $C(S)>0$, for $\varepsilon$ small enough, by (8.34). $\quad \square$

##### Lemma 8.8

Let $|\ell |\le \varepsilon ^{-\beta }$ and $|\overline{\omega }\cdot \ell +{\lambda }(j)-{\lambda }(k) |\le {\gamma _0}{\langle \ell \rangle ^{-\sigma }}$. Then $R_{\ell jk}(\eta ,\sigma )$ satisfies (8.26).

##### Proof

Let us call $p:=\overline{\omega }\cdot \ell +{\lambda }(j)-{\lambda }(k)$ and note that $|p |\lesssim {\gamma }_0=\varepsilon ^{\alpha }$. We also remark that $\ell \ne 0$ since for $j\ne k$ one has $|{\lambda }(j)-{\lambda }(k)|>1/2$. We substitute *p* in the definition of $b_{\ell jk}$ (see (8.32))

$$\begin{aligned} |b_{\ell jk}|= & {} \Big |\ell + (-\overline{\omega }\cdot \ell + p ) \mathbb {A}^{-T} \vec {v} +\mathbb {A}^{-T}(\vec {w}_j-\vec {w}_k)\Big | \gtrsim |\ell - \mathbb {A}^{-T} \vec {v} \overline{\omega }^T \ell \\&+\mathbb {A}^{-T}(\vec {w}_j-\vec {w}_k) |+ \varepsilon ^{\alpha }. \end{aligned}$$

Then, using (A.5) in Lemma (A.1) we have $|b_{\ell jk}|\ge |\ell |\delta /2$ for $\varepsilon$ small enough. The thesis follows reasoning as in Lemma (8.6). $\quad \square$

##### Proof of Lemma 8.4

For the sets $R_{\ell j k}$ the lemma follows by Lemmata 8.6, 8.7, 8.8.

The proof for the sets $Q_{l j}$ and $P_{\ell j}$ follows using the same arguments used for $R_{\ell j k}$. Lemmata 8.6, 8.7 are identical, with the only difference that the non-resonance condition now reads respectively $|\overline{\omega }\cdot \ell + j|\ge \gamma _0\langle \ell \rangle ^{-\sigma }{,} |\overline{\omega }\cdot \ell +{\lambda }(j) |\ge \gamma _0\langle \ell \rangle ^{-\sigma }$ in the case of $Q_{\ell j}$ and $P_{\ell j}$. Regarding Lemma 8.8, it follows from (A.4) in the case of $Q_{\ell j}$ and from (A.6) in the case of $P_{\ell j}$. $\quad \square$

#### Summability

##### Lemma 8.9

For $n\ge 1, |\ell |\le N_{n-1}$, one has $R_{\ell j k}(i_n),Q_{\ell j}(i_n),P_{\ell j}(i_n)=\emptyset$.

##### Proof

We first note that, by Lemma 8.3, if $|{\lambda }(j)-{\lambda }(k)|> C_1^{-1}|\ell |$ (for some $C_1=C_1(S)$) then $R_{\ell j k}(i_n)= R_{\ell j k}(i_{n-1})=\emptyset$, so that our claim is trivial. Otherwise, if

$$\begin{aligned} |{\lambda }(j)-{\lambda }(k)|\le C_1^{-1}|\ell |\le C_1^{-1} N_{n-1}{.} \end{aligned}$$

By (7.101) (with $i_{\delta }^{(1)}\rightsquigarrow i_n$ and $i_{\delta }^{(2)}\rightsquigarrow i_{n-1}$, $N\rightsquigarrow N_{n-1}$) and (8.8) we have for all $j, k\in S^c$

8.36 $$\begin{aligned} |(d_j^{\infty }-d_k^{\infty })(i_n)-(d_j^{\infty }-d_k^{\infty })(i_{n-1})|\le \varepsilon ^{4-3 a} N_{n-1}^{-\mathtt {a}}\qquad \qquad \forall \omega \in \mathcal {G}_n, \end{aligned}$$

where $\mathtt {a}:=\min \{\kappa ,\alpha \}$ (recall $\alpha$ in (8.4) and $\kappa$ in (7.13)). Now for all $j\ne k$, $|\ell |\le N_{n-1}$, $\omega \in \mathcal {G}_n$ by (8.36)

8.37 $$\begin{aligned}&|\omega \cdot \ell +d_j^{\infty }(i_n)-d_k^{\infty }(i_{n})|\ge |\omega \cdot \ell +d_j^{\infty }(i_{n-1})-d_k^{\infty }(i_{n-1})|\nonumber \\&\qquad -|(d_j^{\infty }-d_k^{\infty })(i_n)-(d_j^{\infty }-d_k^{\infty })(i_{n-1})|\nonumber \\&\quad \ge 2\gamma ^{*}_{n-1}\langle \ell \rangle ^{-\tau }-\varepsilon ^{4-3 a}N_n^{-\mathtt {a}}\ge 2\gamma ^{*}_{n}\langle \ell \rangle ^{-\tau } \end{aligned}$$

since $\varepsilon ^{4-3 a}\gamma ^{-3/2}N_n^{\tau -(2/3)\mathtt {a}}2^{n+1}\le 1$. Since by definition $R_{\ell j k}(i_n)\subseteq \mathcal G_n$ then $R_{\ell j k}(i_n)=\emptyset$ .

Now we prove that $Q_{\ell j}(i_{n-1}) \subseteq Q_{\ell j}(i_n)$. We have

8.38 $$\begin{aligned} |m(i_n)-m(i_{n-1}) ||j |{\mathop {\le }\limits ^{(7.48)}}&C \varepsilon ^3 \Vert i_{n}-i_{n-1} \Vert _{s_0+2}|j |{\mathop {\le }\limits ^{(8.8)}} C \varepsilon ^{b_*+3}\gamma ^{-1} N_{n-1}^{-\alpha } |j |\nonumber \\ \le \,\,&\, C \varepsilon ^{b_*+3}\gamma ^{-1} N_{n-1}^{-\alpha } |\ell |\end{aligned}$$

and then

8.39 $$\begin{aligned} |\omega \cdot \ell +m (i_{n}) j |&\ge |\omega \cdot \ell +m (i_{n-1}) j |-|m(i_n)-m(i_{n-1}) ||j |\nonumber \\&\ge 2 \gamma _{n-1} \langle \ell \rangle ^{-\tau }- \varepsilon ^{b_*+3}\gamma ^{-1} N_{n-1}^{-\alpha +1} \ge 2 \gamma _n \langle \ell \rangle ^{-\tau } \end{aligned}$$

since $$|\ell |\le N_{n-1}$$.

As before, by (7.101), for all $j, k\in S^c$

8.40 $$\begin{aligned} |d_j^{\infty }(i_n)-d_j^{\infty }(i_{n-1})|\le \varepsilon ^{4-3 a}N_{n-1}^{-\mathtt {a}}\qquad \qquad \forall \omega \in \mathcal {G}_n. \end{aligned}$$

For all $j\ne k$, $|\ell |\le N_{n-1}$, $\omega \in \mathcal {G}_n$ by (8.36)

8.41 $$\begin{aligned}&|\omega \cdot \ell +d_j^{\infty }(i_n)|\ge |\omega \cdot \ell +d_j^{\infty }(i_{n-1})|-|d_j^{\infty }(i_n)-d_j^{\infty }(i_{n-1})|\nonumber \\&\quad \ge 2\gamma _{n-1} \langle \ell \rangle ^{-\tau }-\varepsilon ^{4-3 a}N_{n-1}^{-\mathtt {a}}\ge 2\gamma _{n} \langle \ell \rangle ^{-\tau } \end{aligned}$$

since $$\varepsilon ^{4-3 a}\gamma ^{-1}N_n^{\tau -(2/3)\mathtt {a}}2^{n+1}\le 1$$. $$\quad \square $$

We have proved that

8.42 $$\begin{aligned} \mathcal {G}_n {\setminus } \mathcal {G}_{n+1}\subseteq \bigcup _{\begin{array}{c} j, k\in S^c\\ |\ell |> N_{n-1} \end{array}}\Big ( R_{\ell j k}(i_n)\cup Q_{\ell j}(i_n)\cup P_{\ell j}(i_n)\Big ), \quad \forall n\ge 1. \end{aligned}$$

##### Lemma 8.10

There exists $\mathtt {C}>0$ such that if $|j |, |k |\ge \mathtt {C} \langle \ell \rangle ^{\nu +2}\gamma ^{-(1/2)}$ then (recall that $\tau =2\nu +6>\nu +2$)

8.43 $$\begin{aligned} R_{\ell j k}(\gamma ^{3/2}, \tau )\subseteq Q_{\ell , j-k}(\gamma , \nu +2). \end{aligned}$$

##### Proof

We have that

8.44 $$\begin{aligned} |\omega \cdot \ell +d_j^{\infty }-d_k^{\infty } |&\ge |\omega \cdot \ell +m(j-k) |-|m ||{\lambda }(j)-j+k-{\lambda }(k) |-\varepsilon ^2 |w_j-w_k |\nonumber \\&\quad -|r_j^{\infty }|-|r_k^{\infty }|\nonumber \\&\ge \frac{2\gamma }{\langle \ell \rangle ^{\nu +2}}-2 |j-k |\frac{C}{|j ||k |}-\frac{\tilde{C}\varepsilon ^2}{\min \{ |j |, |k |\}}\nonumber \\&\ge \frac{2\gamma }{\langle \ell \rangle ^{\nu +2}}- \frac{C\gamma }{\mathtt {C}\langle \ell \rangle ^{2(\nu +2)-1}}-\frac{\tilde{C}\varepsilon ^2 \sqrt{\gamma }}{\mathtt {C} \langle \ell \rangle ^{\nu +2}}\nonumber \\&\ge \frac{\gamma }{\langle \ell \rangle ^{\nu +2}} \Big (2-\frac{C}{2\mathtt {C}\langle \ell \rangle ^{\nu +1}}-\frac{\tilde{C}\varepsilon ^2 }{2\sqrt{\gamma }\mathtt {C} } \Big )\nonumber \\&\ge \frac{\gamma }{\langle \ell \rangle ^{\nu +2}} \ge \frac{\gamma ^{3/2}}{\langle \ell \rangle ^{\tau }} \end{aligned}$$

for $\mathtt {C}$ big enough and since $\varepsilon ^2 (\sqrt{\gamma })^{-1}\ll 1$. $\quad \square$

We are in position to prove (8.9). We have, by (8.42),

$$\begin{aligned} \left|\bigcup _{\ell \in \mathbb {Z}^{\nu }, j, k\in S^c} R_{\ell j k}(i_n) \right|\le & {} \sum _{|\ell |>N_{n-1}, |j |, |k |\ge \mathtt {C}\langle \ell \rangle ^{\nu +2}\gamma ^{-(1/2)}}|R_{\ell j k}(i_n)|\\&+\sum _{|\ell |>N_{n-1}, |j |, |k |\le 2 \mathtt {C} \langle \ell \rangle ^{\nu +2}\gamma ^{-(1/2)}} |R_{\ell j k}(i_n)|. \end{aligned}$$

On one hand we have that, using Lemmata 8.4 and 8.10,

$$\begin{aligned} \sum _{|\ell |>N_{n-1}, |j |, |k |\ge \mathtt {C} \langle \ell \rangle ^{\nu +2}\gamma ^{-(1/2)}}|R_{\ell j k}(i_n)|&\lesssim K\sum _{j-k=h, |h |\le C |\ell |} \varepsilon ^{2(\nu -1)}\gamma \langle \ell \rangle ^{-\nu -2}\\&\lesssim K\varepsilon ^{2(\nu -1)}\gamma \sum _{|\ell |\ge N_{n-1}} \langle \ell \rangle ^{-(\nu +1)}\\&\lesssim K \varepsilon ^{2(\nu -1)}{\gamma }N_{n-1}^{-1}. \end{aligned}$$

On the other hand

$$\begin{aligned} \sum _{\begin{array}{c} |\ell |>N_{n-1}, |j-k |\le C|\ell |\\ |j |, |k |\le 2\mathtt {C} \langle \ell \rangle ^{\tau _1}\gamma ^{-(1/2)} \end{array}} |R_{\ell j k} (i_n)|&\lesssim K\gamma ^{(3/2)} \varepsilon ^{2(\nu -1)} \sum _{|\ell |\ge N_{n-1}} \frac{|\ell |\langle \ell \rangle ^{\nu +2}}{\sqrt{\gamma }\langle \ell \rangle ^{\tau }}\\&\lesssim K\gamma \varepsilon ^{2(\nu -1)} \sum _{|\ell |\ge N_{n-1}} \langle \ell \rangle ^{-(\tau -\nu -3)}\\&\lesssim K \gamma \varepsilon ^{2(\nu -1)} N_{n-1}^{-1}. \end{aligned}$$

The discussion above implies estimates (8.9).

### Conclusion of the Proof of Theorem 5.4

Theorem 8.1 implies that the sequence $(\mathfrak {I}_n, \zeta _n)$ is well defined for $\omega \in \mathcal {G}_{\infty }:=\cap _{n\ge 0} \mathcal {G}_n$, $\mathfrak {I}_n$ is a Cauchy sequence in $\Vert \cdot \Vert _{s_0+\mu }^{{\gamma },\mathcal {G}_{\infty }}$ (see (8.8)) and $|\zeta _n |^{\gamma }\rightarrow 0$. Therefore $\mathfrak {I}_n$ converges to a limit $\mathfrak {I}_{\infty }$ in norm $\Vert \cdot \Vert _{s_0+\mu }^{\gamma ,\mathcal {G}_{\infty }}$ and, by $(\mathcal {P} 2)_n$, for all $\omega \in \mathcal {G}_{\infty }, i_{\infty }(\varphi ):=(\varphi , 0, 0)+\mathfrak {I}_{\infty }(\varphi )$ is a solution of

8.45 $$\begin{aligned} \mathcal {F}(i_{\infty }, 0)=0 \qquad \text{ with } \qquad \Vert \mathfrak {I}_{\infty } \Vert _{s_0+\mu }^{\gamma ,\mathcal {G}_{\infty }}\lesssim \,\varepsilon ^{9- 2 b} \gamma ^{-1} \end{aligned}$$

by (8.7), (8.5). Therefore $\varphi \mapsto i_{\infty }(\varphi )$ is an invariant torus for the Hamiltonian vector field $X_{H_{\varepsilon }}$ (recall (5.1)). By (8.9),

$$\begin{aligned} |\Omega _{\varepsilon }{\setminus } \mathcal {G}_{\infty } |\le & {} |\Omega _{\varepsilon } {\setminus } \mathcal {G}_0 |+\sum _{n\ge 0} |\mathcal {G}_n {\setminus } \mathcal {G}_{n+1} |\le 2\,C_* \varepsilon ^{2 (\nu -1)} \gamma \nonumber \\&+C_* \varepsilon ^{2(\nu -1)}\gamma \sum _{n\ge 1} N_{n-1}^{-1} \lesssim C_{*}\varepsilon ^{2(\nu -1)} \gamma . \end{aligned}$$

The set $\Omega _{\varepsilon }$ in (5.2) has measure $|\Omega _{\varepsilon } |=O(\varepsilon ^{2 \nu })$. Hence $|\Omega _{\varepsilon }{\setminus } \mathcal {G}_{\infty } |/|\Omega _{\varepsilon } |\rightarrow 0$ as $\varepsilon \rightarrow 0$ because $\gamma =o(\varepsilon ^2)$, and therefore the measure of $\mathcal {C}_{\varepsilon }:=\mathcal {G}_{\infty }$ satisfies (5.12).

It remains to show the linear stability of the embedding $i_{\infty }({\varphi })$. By the discussion of Sect. 6 (see also [4] for further details) and Sect. 7, since $i_{\infty }({\varphi })$ is isotropic and solves the Eq. (8.45), it is possible to find a change of coordinates $G_{\infty }$ (of the form (6.6)), so that in the linearized system of the Hamiltonian $H_{\varepsilon }\circ G_{\infty }(\varphi , \eta , w)$ the equation for the actions is given by $\dot{\eta }=0$. Moreover, by Sect. 7 the linear equation for the normal variables *w* is conjugated, by setting $w=\Upsilon \circ \Phi _{\infty }(z)$ to the diagonal system $\dot{z}_j-\mathrm{i}d_j^{\infty }(\omega )z_j=f_{j}(\omega t), j\in S^{c}$, where $f(\omega t)$ is a forcing term.

Since $d_j^{\infty }\in \mathbb {R}$ a standard argument shows that the Sobolev norms of *w* do not increase in time. For further details see [2, 4, 40].

## Appendix A. Non-degeneracy conditions

### Proof of Lemma 4.1

Recalling (4.6) we introduce a matrix $\mathbb K$ so that $\mathbb {A}=: (2/9)\,\, \mathrm {diag}\Big ({\lambda }(\overline{\jmath }_i)(1+\overline{\jmath }_i^2)\Big ) \mathbb K$. Now we show that the entries of $\mathbb K$ are bounded by some constant independent of the $\overline{\jmath }_i$. After some direct computations we have that

$$\begin{aligned}&\mathbb {K}_{j j}=\frac{1+j^2}{j^2}{,} \quad j\in S^+{,}\\&\quad \mathbb {K}_{j k}= 3 \frac{(1+k^2)(2+k^2+j^2)}{(3+k^2+j^2+k j)(3+k^2+j^2-k j)}{,} \quad j, k\in S^+,\,\, j\ne k{.} \end{aligned}$$

Obviously $|\mathbb {K}_{j j} |\le 2$; regarding the off-diagonal terms, we note that $0\le 2 k j \le k^2+j^2$, hence $|\mathbb {K}_{j k} |\le 12$ if $j\ne k$.

We consider the variables $x, p_2, \ldots , p_{\nu }$ defined as

A.1 $$\begin{aligned} \overline{\jmath }_1 =: 1/x{,}\quad \overline{\jmath }_i =: p_i/x{,}\quad 0<p_i\le 1{,} \end{aligned}$$

so that $P(x,p_i)=\det (\mathbb K)$ is a rational function. It is easily seen that $\mathbb K$ computed at $p_i=1$ for all *i*, coincides with the matrix

$$\begin{aligned} (1+x^2)\,\big (\mathrm {I}+2\,g(x)\,(U-\mathrm {I})\big ){,} \qquad g(x):=(3 x^{2}+1)^{-1}{,} \end{aligned}$$

where *U* is the matrix with components $U_{ij}=1$ for any $i,j=1, \ldots , \nu$. Its determinant is

A.2 $$\begin{aligned} \left( \frac{1+x^2}{1+3 x^2}\right) ^{\nu } (3 x^2-1)^{\nu -1}(3 x^2+2 \nu -1){.} \end{aligned}$$

We note that the absolute value of (A.2) is $\ge 1$ at $x=0$. We conclude that there exists $0<\mathtt {r}_0<1$ such that

$$\begin{aligned} \text{ if }\quad 0\le x<\mathtt {r}_0{,}\,\,\, |p_i-1|\le \mathtt {r}_0 \quad \text{ then } \quad |P(x, p_i) |\ge 1/2{.} \end{aligned}$$

This implies the thesis. $\quad \square$

### Lemma A.1

There exists $0<\mathtt {r}_0<1$ such that, for any $S^+\in \mathcal {V}(\mathtt {r})$ with $0<\mathtt {r}\le \mathtt {r}_0$ (see Definition 1.1), the following holds true:

A.3 $$\begin{aligned}&\bullet \quad |\sum _{i=1}^{\nu } \frac{\overline{\jmath }_i}{1+\overline{\jmath }_i^2}\,\ell _i| > \frac{\mathtt {r}}{2}\ne 0 {,} \quad \forall \ell \in \mathbb {Z}^\nu {,} \quad |\ell |=1,2,3,5\,; \end{aligned}$$

A.4 $$\begin{aligned}&\bullet \quad \left| \mathrm{det}\Big ( \mathrm{I} -\mathbb {A}^{-T} \vec {v} (\overline{\omega })^T\Big ) \right| \ge 1\,; \end{aligned}$$

A.5 $$\begin{aligned}&\bullet \quad |\ell - \Big ( \mathrm{I}-\mathbb {A}^{-T} \vec {v} (\overline{\omega })^T\Big )^{-1} \mathbb {A}^{-T}(\vec {w}_j-\vec {w}_k)| \ge \delta |\ell | {,} \qquad \ell \in \mathbb {Z}^\nu , \; j, k\in S^c \end{aligned}$$

A.6 $$\begin{aligned}&\bullet \quad |\ell - \Big ( \mathrm{I}-\mathbb {A}^{-T} \vec {v} (\overline{\omega })^T\Big )^{-1} \mathbb {A}^{-T}\vec {w}_j| \ge \delta |\ell |{,} \qquad \ell \in \mathbb {Z}^\nu {,} \; j \in S^c \end{aligned}$$

Here $\vec {v}$ and $\vec {w}_j$ are defined in (8.28) (see also (7.5)), $\overline{\omega }$ in (1.10) and $\delta$ is some appropriately small pure constant.

### Proof of (A.3)

The case $|\ell |=1$ is trivial. For $|\ell |=2$ we use the fact that the $\overline{\jmath }_i$ are all distinct. For $|\ell |=3,5$ we pass to the variables (A.1) and we get

$$\begin{aligned} \overline{\jmath }_1 |\sum _{i=1}^{\nu } \frac{\overline{\jmath }_i}{1+\overline{\jmath }_i^2}\, \ell _i| = |\sum _{i=1}^{\nu } \frac{p_i}{x^2+p_i^2}\,\ell _i| =: L(x,p){.} \end{aligned}$$

We notice that $L(0,1)=|\sum _i \ell _i|\ge 1$ (since $\sum _i \ell _i$ has the same parity of $|\ell |$) so, by continuity, there exists $0<\mathtt {r}_0<1$ such that $L(x,p)>1/2$ for all $0\le x<\mathtt {r}_0,\,\,\, |p_i-1|\le \mathtt {r}_0$. This implies the result. $\quad \square$

### Proof of (A.4)

We first note that (recall (8.28))

A.7 $$\begin{aligned} \det (\mathrm {I} -\mathbb {A}^{-T}\vec {v}\overline{\omega }^{T}) =1-\mathbb {A}^{-T}\vec {v}\cdot \overline{\omega }{.} \end{aligned}$$

Consider the change of variables (A.1). One can note that the matrix $\mathbb {A}$ in (4.6) at $p_i=1$, $i=2, \ldots , \nu$, is given by

A.8 $$\begin{aligned} \mathbb {A}&:=d(x)\big [\mathrm {I}+e(x)U\big ]{,} \qquad \mathbb {A}^{-1}:=\frac{1}{d(x)}\big [\mathrm {I}-f(x)U\big ]{,}\nonumber \\ d(x)&:=\frac{2(4x^2+1)(3x^4+2x^2+1)}{9 x^3(3x^2+1)}{,} \qquad e(x):=\frac{2x^2}{3x^4+2x^{2}+1}{,} \quad f(x):=\frac{e(x)}{1+\nu e(x)}{.} \end{aligned}$$

By (A.8) at $p_i=1$ we have

A.9 $$\begin{aligned} \mathbb {A}^{-1}\vec {v}\cdot \overline{\omega } =\frac{2(4x^2+ 1) }{3 x}(\vec {1}\cdot \mathbb {A}^{-1}\vec {1}) =3\nu \,\,\frac{(4x^2+1) (3x^2+1)}{(4x^2+1)(3x^4+2(1+\nu )x^2+1)}{.} \end{aligned}$$

We note that, for $x=0$, one has $|\det (\mathrm {I} -\mathbb {A}^{-1}\vec {v}\overline{\omega }^{T}) |=3\nu -1\ge 2$. Thus there is $0<\mathtt {r}_0<1$ so that for all $0\le x<\mathtt {r}_0$, $|p_i-1|\le \mathtt {r}_0$ one has $|\det (\mathrm {I} -\mathbb {A}^{-1}\vec {v}\overline{\omega }^{T})|\ge 1$.

$$\square $$

### Proof of (A.5),(A.6)

We systematically use the variables (A.1). We define $\Omega \!=\!\,\text{ diag }\,({\overline{\omega }_i})$, $V=\,\text{ diag }\,(\vec {v}_i)$ and write $\mathbb {A}=\Omega \mathbb {H}\,V$ where

$$\begin{aligned} \mathbb H = \frac{x^2+1}{3} [ I + \frac{2}{(3x^2+1)} (U-I)] + O(|p-1|) =-\frac{1}{3} (I-2 U)+O(|p-1 |, |x |){.} \end{aligned}$$

Then $\vec {w}_j$ in (8.28) can be written as $\vec {w}_j=\Omega \,V\,b_j$ with

$$\begin{aligned} b_j \cdot \mathtt {e}_i = -\frac{{\lambda }(j)(7+5 \overline{\jmath }_i^2 +\overline{\jmath }_i^4+3 j^2)}{{\lambda }(\overline{\jmath }_i)((3+j^2)^2+(6+j^2) \overline{\jmath }_i^2 +\overline{\jmath }_i^4)}, \end{aligned}$$

where $\mathtt {e}_i$ is the *i*-th vector of the canonical basis of $\mathbb {R}^{\nu }$. In the new coordinates (*x*, *p*) in (A.1), and setting $t=j (\overline{\jmath }_1)^{-1}$, this reads as

$$\begin{aligned}&b_j \cdot \mathtt {e}_i=:b(t, x, p)\cdot \mathtt {e}_i = f(t, x) g(t, x, p_i)+\,t\,g(t, x, p_i){,} \\&f(t,x):= \frac{3\,t\,x^2}{x^2+t^2}{,} \qquad g(t, x, p):= \frac{(x^2+p_i^2)(7x^4+5 x^2p_i^2+p_i^4+3x^2 t^2)}{p_i\,(4x^2+p_i^2) ((3x^2+t^2)^2+(6x^2+t^2)p_i^2+p_i^4)}{.} \end{aligned}$$

We claim that

A.10 $$\begin{aligned} b(t, x, p) =-\frac{t}{t^4+t^2+1} \vec {1}+ O(|p-1|, |x |) \end{aligned}$$

where the term $$ O(|p-1|, |x |)$$ is uniform in *t*.

By direct computations we have

$$\begin{aligned} |g(t, x, p)-g(t, 0, 1)|\lesssim C\big (|x |+|p-1 |\big )(1+t^2)^{-1} {,}\quad |g(t, x, p) |\le C(1+t^{2})^{-1}{,} \end{aligned}$$

with *C* independent of *t*, and for *x*, *p* in a neighborhood of (0, 1). Moreover $\sup _{t} |f(t, x) |\le 3|x|^{2}/2$. Thus, for *x*, *p* sufficiently close to (0, 1), we have that

$$\begin{aligned}&|b(t, x, p)-b(t, 0, 1)|\le |t ||g(t, x, p)-g(t, 0, 1)|+|f(t, x) ||g(t, x, p) |\\&\quad \lesssim O(|p-1|,|x|){,} \end{aligned}$$

which implies the claim (A.10). Hence, setting $s=k (\overline{\jmath }_1)^{-1}$, we have

$$\begin{aligned}&\ell -\Omega ^{-1} (\mathbb {H}^T-U)^{-1} \Omega (b(t, x , p)-b(s, x, p))\\&\quad = \ell - 3(1+\nu )^{-1} h(t, s)\,\vec {1}+O(|p-1 |, |x |){,} \end{aligned}$$

where

$$\begin{aligned} h(t, s):= t\, (t^{4}+t^{2}+1)^{-1}- s\, (s^{4}+s^{2}+1)^{-1}{.} \end{aligned}$$

We note that $|t\, (t^4+t^{2}+1)^{-1} |\le 0.41$, hence each single component satisfies

$$\begin{aligned} | 3(1+\nu )^{-1} h(t, s)+O(|p-1 |, |x |)\cdot \mathtt {e}_i| \le 3 (1+\nu )^{-1} (1-\delta ) \le (1-\delta ) \end{aligned}$$

provided that $|p-1|,|x| \le \mathtt {r}_0$ small enough. Hence, if $\nu \ge 2$, one has $|\ell _i- \big (\frac{3 h(t, s)}{1+\nu }\,\vec {1}+O(|p-1 |, |x |)\big )\cdot \mathtt {e}_i |\ge \delta |\ell _i|{.}$ This concludes the proof of (A.5). The proof of (A.6) is the same just setting $h(t)= t\, (t^4+t^2+1)^{-1}$. $\quad \square$

### Proof of Lemma 5.1

It is well known that, thanks to the choice of $\tau$ in (5.3), $|\Omega _{\varepsilon }{\setminus }\mathcal {G}_0^{(0)} |\le C \varepsilon ^{2(\nu -1)}\gamma$ for some $C=C(S)>0$. Thus we focus on the estimate for the measure of $\mathcal {G}_0^{(1)}$. For indexes $\ell \in \mathbb {Z}{\setminus }\{0\}$, $j,k\in S^{c}$ satisfying

A.11 $$\begin{aligned} |\ell |\le 3{,}\qquad \mathrm{and}\qquad \sum _{i=1}^{\nu } \overline{\jmath }_i\,\ell _i+j=k{,} \end{aligned}$$

we define the sets $T_{\ell j k}:=\{ \omega \in \Omega _{\varepsilon } : |\overline{\omega }\cdot \ell +\varepsilon ^2 \mathbb {A}\xi \cdot \ell + {\lambda }(j)-{\lambda }(k)+\varepsilon ^2 ({\lambda }(j)\mathfrak {l}_{j}-{\lambda }(k)\mathfrak {l}_k) |\le C \gamma \}$. Recalling (5.5) we have that $\Omega _{\varepsilon }{\setminus }\mathcal {G}^{(1)}_{0}=\bigcup T_{\ell j k}$ where the union is restricted to $\ell ,j,k$ satisfying (A.11).

Let us first study the $\varepsilon$-independent part of our small divisor. By (1.8) and (A.11) we have

A.12 $$\begin{aligned} \overline{\omega }\cdot \ell +{\lambda }(j)-{\lambda }(k)=3\sum _{i=1}^{\nu } \frac{\overline{\jmath }_i}{1+\overline{\jmath }_i^2}\,\ell _i+3\frac{j}{1+j^2}-3\frac{(\sum _{i=1}^{\nu } \overline{\jmath }_i\,\ell _i+j)}{1+(\sum _{i=1}^{\nu } \overline{\jmath }_i\,\ell _i+j)^2}. \end{aligned}$$

By Lemma A.1 (see (A.3)) if $|j|\ge \widehat{C}(S)$, which is easily computed in terms of $\mathtt {r}$, (A.12) is bounded from below by $\mathtt {r}/4$. By (A.11), $|{\lambda }(j)-{\lambda }(k) |\le C(S)$. Since $0<|\ell |\le 3$ and substituting $\mathfrak {l}_j=c(\omega )+\kappa _j/{\lambda }(j)$ (recall Lemma 7.10), where $c(\omega ),\kappa _j$ are defined in (7.2), (7.5))

$$\begin{aligned} |\overline{\omega }\cdot \ell +\varepsilon ^{2}\mathbb {A}\xi \cdot \ell +(1+\varepsilon ^2 c(\omega )) ({\lambda }(j)-{\lambda }(k))+\varepsilon ^2 (\kappa _j-\kappa _k)| \ge \mathtt {r}/8 \end{aligned}$$

for $\varepsilon$ small enough (depending on $\mathtt {r}$) and by using the fact that $\kappa _j-\kappa _k$ is uniformly bounded in *j*, *k*. This implies that $T_{\ell jk}=\emptyset$ for $\varepsilon$ small enough.

We are left to deal with the case $|j|\le \widehat{C}(S)$. We write (A.12) as $P(\overline{\jmath }_i,j)/Q(\overline{\jmath }_i, j)$ where *P*, *Q* are polynomials with integer coefficients and *Q* has no real zeros. We remark that $1<Q< C(S)$ due to the condition $|j|\le \widehat{C}(S)$. If $P\ne 0$ then $|P|\ge 1$ and again (A.12) is larger than some *K*(*S*). We conclude that $T_{\ell jk}=\emptyset$ by reasoning as in the case *j* large. Now we study the case in which $P=0$. Fixed $\ell$ in (A.12), then *P* has degree four in *j* and so the condition $P=0$ fixes at most four choices of *j* that we call $\widehat{\jmath }_1, \widehat{\jmath }_2, \widehat{\jmath }_3, \widehat{\jmath }_4$. For $P=0$ (which is (A.12) $=\,0$) we have

A.13 $$\begin{aligned} \overline{\omega }\cdot \ell +\varepsilon ^{2}\mathbb {A}\xi \cdot \ell +\lambda (j)(1+\varepsilon ^{2}\mathfrak {l}_j)-\lambda (k)(1+\varepsilon ^{2}\mathfrak {l}_k) =\varepsilon ^2( {\mathbb A}\xi \cdot \ell -(\overline{\omega }\cdot \ell ) \vec {v}\cdot \xi +( w_{j}-w_{k})\cdot \xi ){,} \end{aligned}$$

where $\vec {v}$ is in (8.28) and $\kappa _j=w_j\cdot \xi$ with $\kappa _j$ in (7.5). These are a finite number (depending only on $\nu$) of linear functions of $\xi$. We compute the derivative in $\xi$ which is

A.14 $$\begin{aligned} ({\mathbb A}^T + \vec {v}\overline{\omega }^T )\ell + ( w_{\widehat{\jmath }}-w_{k}){,} \end{aligned}$$

where $\widehat{\jmath }\in \{ \widehat{\jmath }_1, \widehat{\jmath }_2, \widehat{\jmath }_3, \widehat{\jmath }_4 \}$. Now (A.5) implies that the quantity (A.14) is bounded from below by a constant depending on *S*. This lower bound and Fubini Theorem imply that $|T_{\ell jk}|\le C(S)\varepsilon ^{2(\nu -1)}{\gamma }$ for some $C(S)>0$ depending on *S*. By the discussion above we have

$$\begin{aligned} |\Omega _{\varepsilon }{\setminus } \mathcal {G}_{0}^{(1)}|\le \sum _{|\ell |\le 3, |j|,|k|\le K(S)}|T_{\ell jk}| \le C(S)\varepsilon ^{2(\nu -1)}{\gamma }, \end{aligned}$$

where $K(S)>0$ and $C(S)>0$ are constant depending on the set *S*. This implies the thesis. $\quad \square$

## Appendix B. Normal form identification

### Proof of Theorem 7.9

The core of Theorem 7.9 is to show that the terms in the l.h.s. of (7.78), which are obtained through a rather complicated sets of bounded changes of coordinates, coincide with the ones obtained by a purely formal full Birkhoff Normal Form procedure. In [32] it has been shown that, at purely formal level, the latter is well-defined and not resonant, i.e. the resonant Hamiltonian is supported only on trivial resonances as in (3.11). We procede as follows.

**Step 1** The first step is to show that resonant terms at order $\varepsilon ^{2}$ of the Full Birkhoff normal form coincide with the ones obtained by using the *weak* BNF procedure in Sect. 3, passing to action-angle variables and finally using a *formal linear* BNF.

**Step 2** In order to conclude we note that the bounded maps we applied in Sects. 7.1 and 7.2 are, as functions of $\varepsilon$, $C^{3}$ with values in $\mathcal {L}(H^{s}, H^{s-3})$ . Therefore the Taylor expansion of the Hamiltonian associated to the operators in (7.69) coincides with the Lie series of the generator up to order $\varepsilon ^2$ (see also (7.71)). Then we show that the Lie series coincides (up to order $\varepsilon ^2$) to the one obtained in step 1. Even though we taylor our proof to the particular set of changes of variables that we use in Sect. 7, the argument is quite general and is essentially that the *linear* BNF up to order $\varepsilon ^2$ is coordinate independent.

### Formal equivalence between *weak*$+$*linear* and *full* BNF procedure

One step of formal BNF means that we apply the formal change of variables generated by

B.1 $$\begin{aligned} \mathfrak {F}^{(3)}:=[\mathrm {ad}_{H^{(2)}}]^{-1}H^{(3)} \end{aligned}$$

which removes completely $H^{(3)}$ and conjugates *H* (using (2.5)) to $H\circ {\Phi }_{(\mathfrak {F}^{(3)})^{-1}}=H^{(2)}+\frac{1}{2} \{\mathfrak {F}^{(3)}, H^{(3)}\} +$ h.o.t.

The scecond step of formal BNF removes all the non-resonant terms of degree four thus we get

$$\begin{aligned} H^{(2)}+\frac{1}{2}\Pi _{{\text{ Ker }}(H^{(2)})} \{\mathfrak {F}^{(3)},H^{(3)}\} +\text{ h.o.t. } \end{aligned}$$

In [32] it has been proved that $\big (\Pi _{{\text{ Ker }}(H^{(2)})} -\Pi _{\mathrm{triv}}\big ) \{\mathfrak {F}^{(3)}, H^{(3)}\}= 0$ (see the notation of Definition 7.8) and hence

B.2 $$\begin{aligned} \Pi ^{d_z=2}\Pi _{{\text{ Ker }}(H^{(2)})} \{\mathfrak {F}^{(3)},H^{(3)}\} = \Pi ^{d_z=2}\Pi _{\mathrm{triv}}\{\mathfrak {F}^{(3)},H^{(3)}\}{.} \end{aligned}$$

We claim that

B.3 $$\begin{aligned} \Pi _{\mathrm{triv}}\{\mathfrak {F}^{(3,1)},H^{(3,3)}\}= & {} \Pi _{\mathrm{triv}}\{\mathfrak {F}^{(3,3)},H^{(3,1)}\}= \Pi _{\mathrm{triv}}\{\mathfrak {F}^{(3,2)},H^{(3,0)}\}\nonumber \\= & {} \Pi _{\mathrm{triv}} \{\mathfrak {F}^{(3,0)},H^{(3,2)}\}=0{.} \end{aligned}$$

This implies that

B.4 $$\begin{aligned}&\Pi _{{\text{ Ker }}(H^{(2)})} \Pi ^{d_z=2}\{\mathfrak {F}^{(3)},H^{(3)}\}\nonumber \\&\quad = \Pi _{\mathrm{triv}}\Pi ^{d_z=2}\Big ( \{\mathfrak {F}^{(3,\le 1)},H^{(3,\le 1)}\}+\{\mathfrak {F}^{(3,\le 1)},H^{(3,>1)}\} \Big )\nonumber \\&\qquad +\Pi _{\mathrm{triv}}\Pi ^{d_z=2}\Big ( \{\mathfrak {F}^{(3,2)},H^{(3,0)}\}+\{\mathfrak {F}^{(3,2)},H^{(3,2)},\} +\{\mathfrak {F}^{(3,3)},H^{(3,1)}\} \Big )\nonumber \\&\quad =\Pi _{\mathrm{triv}}\Pi ^{d_z=2}\Big ( \{\mathfrak {F}^{(3,\le 1)},H^{(3,\le 1)}\}+\{\mathfrak {F}^{(3,2)},H^{(3,2)}\} \Big ){.} \end{aligned}$$

#### Proof of the claim (B.3)

Let us consider the term $\{\mathfrak {F}^{(3,1)},H^{(3,3)}\}$, where

$$\begin{aligned} \mathfrak {F}^{(3,1)}=\sum _{\begin{array}{c} j_1,j_2\in S,j_3\in S^c\\ j_1+j_2+j_3=0 \end{array}}C_{j_1j_2j_3}u_{j_1}u_{j_2}u_{j_3}{,} \qquad H^{(3,3)}=\sum _{\begin{array}{c} j_1,j_2,j_3 \in S^c \\ j_1+j_2+j_3=0 \end{array}} \widetilde{C}_{j_1j_2j_3}u_{j_1}u_{j_2}u_{j_3}{,} \end{aligned}$$

for some coefficients ${C}_{j_1j_2j_3}, \widetilde{C}_{j_1j_2j_3}\in \mathbb {C}$. Using (2.2) one gets

B.5 $$\begin{aligned} \{\mathfrak {F}^{(3,1)},H^{(3,3)}\}=\sum _{\begin{array}{c} j_1,j_2\in S, \, k_1,k_2\in S^{c} \\ j_1+j_2+k_1+k_2=0 \end{array}} P_{j_1j_2k_1k_2}u_{j_1}u_{j_2}u_{k_1}u_{k_2},\qquad P_{j_1j_2k_1k_2}\in \mathbb {C}{.} \end{aligned}$$

A monomial $u_{j_1}u_{j_2}u_{k_1}u_{k_2}$ is supported on trivial resonances (3.11) only if $j_1=-j_2$ and $k_1=-k_2$, since $j_i\in S$, $k_i\in S^{c}$, $i=1,2$. On the other hand, by the momentum condition, we have that $j_1+j_2=-(k_1+k_2)$, which is not possible since $0\notin S\cup S^{c}$ (see (1.9)). Hence the (B.3) holds. The others equalities in (B.3) in follows in the same way. $\quad \square$

Let us now perform the same Birkhoff procedure by first cancelling the terms of degree $\le 1$ in *z* (weak BNF) and then the terms of degree two (linear BNF).

By the discussion in Sect. 3, recall the notations of Proposition 3.6, we have that, after two steps of weak Birkhoff normal form, the Hamiltonian of degree less or equal than 4 is

B.6 $$\begin{aligned} \Pi ^{d\le 4} (H\circ \Phi ^{-1}_2)=H^{(2)}+ Z^{(4,0)}_2+H_{2}^{( 3, \ge 2)} +H_{2}^{(4, \ge 2)}. \end{aligned}$$

Here $Z^{(4,0)}_2$ is defined in (3.23),

B.7 $$\begin{aligned} H_2^{(3, 2)}=H_1^{(3, 2)}=H^{(3,2)}, \qquad H_2^{(4,\ge 2)}=H_1^{(4,\ge 2)}, \end{aligned}$$

where $H_1^{(3)}, H_1^{(4)}$ are defined in (3.19). The monomials of degree greater than 4 will be not involved in this computation, so we omit them. It is important to notice that, by direct inspection, $\mathfrak {F}^{(3,\le 1)}= {F}^{(3,\le 1)}$ defined in formula (3.21). By Proposition 3.6, we know that the same change of variables puts one of the constants of motion (lets say $K_3$ and drop the subindex 3) into normal form,

$$\begin{aligned} \Pi ^{d\le 4} (K\circ \Phi ^{-1}_2)=K^{(2)}+ W^{(4,0)}_2+K_{2}^{( 3, \ge 2)} +K_{2}^{(4, \ge 2)}. \end{aligned}$$

The step of formal linear BNF entails applying the formal change of variables generated by

B.8 $$\begin{aligned} {F}^{(3,2)}:=[\mathrm {ad}_{H^{(2)}}]^{-1}H^{(3,2)}{.} \end{aligned}$$

Again, by direct inspection, one can note that $F^{(3,2)}\equiv \mathfrak {F}^{(3,2)}:=\Pi ^{d_{x}=2}\mathfrak {F}^{(3)}$, where $\mathfrak {F}^{(3)}$ is given in (B.1). Thus obtaining the Hamiltonian

B.9 $$\begin{aligned} H^{(2)}+ Z^{(4,0)}_2+ H_{2}^{( 3, 3)} +\frac{1}{2} \{\mathfrak {F}^{(3,2)},H_2^{(3,\ge 2)}\} + H_2^{(4,\ge 2)} + h.o.t. \end{aligned}$$

Since (B.8) puts in normal form also $K\circ \Phi ^{-1}_2$ following the same reasoning as in [32] and Proposition 3.6, we get that

B.10 $$\begin{aligned}&\Pi ^{d_z=2}\Pi _{{\text{ Ker }}(H^{(2)})} ( \frac{1}{2} \{\mathfrak {F}^{(3,2)}, H_2^{(3,\ge 2)}\}+ H_2^{(4,\ge 2)})\nonumber \\&\quad = \Pi ^{d_z=2}\Pi _{\mathrm{triv}} (\frac{1}{2} \{\mathfrak {F}^{(3,2)},H_2^{(3,\ge 2)}\} + H_2^{(4,\ge 2)})\nonumber \\&\quad =\Pi ^{d_z=2}\Pi _{\mathrm{triv}} (\frac{1}{2} \{\mathfrak {F}^{(3,2)},H_2^{(3, 2)}\} + \frac{1}{2} \{\mathfrak {F}^{(3,\le 1)}, H^{(3, \le 1)}\}+\{ \mathfrak {F}^{(3,1)}, H^{(3, 3)}\})\nonumber \\&\quad {\mathop {=}\limits ^{(\hbox {B.3})}} \frac{1}{2} \Pi ^{d_z=2}\Pi _{\mathrm{triv}} ( \{\mathfrak {F}^{(3,2)}, H_2^{(3, 2)}\} + \frac{1}{2} \{\mathfrak {F}^{(3,\le 1)}, H^{(3, \le 1)}\}) \nonumber \\&\quad {\mathop {=}\limits ^{(\hbox {B.4}),(\hbox {B.2})}} \frac{1}{2} \Pi ^{d_z=2}\Pi _{{\text{ triv }}} \{\mathfrak {F}^{(3)}, H^{(3)}\}{.} \end{aligned}$$

We now want to pass to the action-angle variables introduced in (4.7). Since the rescaling with the parameter $\varepsilon$ is covariant under the change of variables that we use, we consider instead of $A_{\varepsilon }$ the symplectic change of variables $A_1:=A_{\varepsilon _{|_{\varepsilon =1}}}$. By recalling that

$$\begin{aligned} \mathsf {H}_0:=H^{(2)}\circ A_{{1{|_{ \begin{array}{c} y=0\\ \theta ={\varphi } \end{array}}}}}{,} \quad \mathsf {H}_1:= H_2^{(3, 2)} \circ {A}_{{1{|_{ \begin{array}{c} y=0\\ \theta ={\varphi } \end{array}}}}} {,} \,\, \Pi ^{d_z=2}\big (\mathsf {H}_2+\mathsf {H}_{\mathcal {R}_2}\big ):= H_2^{(4, 2)}\circ {A}_{{1{|_{ \begin{array}{c} y=0\\ \theta ={\varphi } \end{array}}}}} \end{aligned}$$

and setting

B.11 $$\begin{aligned} \widetilde{F}^{(3, 2)}:=\mathfrak {F}^{(3, 2)} \circ {A}_{{1{|_{ \begin{array}{c} y=0\\ \theta ={\varphi } \end{array}}}}}{,} \end{aligned}$$

we have that (B.10) reads

B.12 $$\begin{aligned} \Pi ^{d_z=2}\Pi _{{\text{ Ker }}(\mathsf {H}_0)} ( \frac{1}{2} \{\widetilde{F}^{(3,2)}, \mathsf {H}_1\}+\mathsf {H}_2+\mathsf {H}_{\mathcal {R}_2 })= \frac{1}{2}\left[ \Pi ^{d_z=2}\Pi _{{\text{ triv }}} \{\mathfrak {F}^{(3)}, H^{(3)}\}\right] \circ A_{{1{|_{ \begin{array}{c} y=0\\ \theta ={\varphi } \end{array}}}}}{.} \end{aligned}$$

### The *rigorous* procedure of subsections 7.1, 7.2 and the *linear* BNF

Since the r.h.s. of (B.12) is the r.h.s. of (7.78) it remains to show that the $\varepsilon ^{2}$-terms of the Hamiltonian associated to the operator $\mathcal {L}_{7}$ in (7.69) coincides with the l.h.s. in (B.12). We remark that the operator $\mathcal {L}_7$ has been constructed through a rigorous procedure providing also “tame” estimates of the remainder of higher order in $\varepsilon$. As already explained the maps $\mathcal {B}$, $\Upsilon _i$, $i=1,2$, are, as functions of $\varepsilon$, $C^{3}$ with values in $\mathcal {L}(H^{s}, H^{s-3})$ . Therefore the Taylor expansion of the Hamiltonian associated to the operators in (7.69) coincides with the Lie series of the generator up to order $\varepsilon ^2$ (see also (7.71)).

Let us then Taylor expand the Hamiltonian $\mathcal {K}^{(2)}=\mathsf {H}\circ \mathcal {B}^{-1}\circ \Upsilon _1^{-1}\circ {\Upsilon _2}^{-1}$ (see (7.56), (7.57), (7.65) and (7.76)) up to order $\varepsilon ^{2}$. It is sufficient to consider $\mathcal {B}_1$ the flow generated by the Hamiltonian in (7.20) and $\Upsilon _1$ the flow of the Hamiltonian $H_{\mathtt {A_1}}$ (which has the form (C.47) and satisfies (7.66)). We have that

B.13 $$\begin{aligned}&\Pi _{\mathrm{Ker}(\mathtt {H}_0)} \Big (\mathtt {Z}_0+ \mathcal {K}^{(1)}_2\Big ){\mathop {=}\limits ^{(7.76),(7.77)}}\Pi _{\mathrm{Ker}(\mathtt {H}_0)} \mathsf {H}\circ {\mathcal {B}}_1^{-1}\circ \Upsilon _1^{-1}+ O(\varepsilon ^3) \end{aligned}$$

B.14 $$\begin{aligned}&{\mathop {=}\limits ^{(7.43),(7.65)}}\mathtt {H}_0+\varepsilon ^{2} \Pi _{\mathrm{Ker}(\mathtt {H}_0)}\Big (\mathsf {H}_2^{(2)}+ \frac{1}{2}\{ H_{\mathtt {A}_1}, \{H_{\mathtt {A}_1},\mathsf {H}_0\}_e\}_e +\{H_{\mathtt {A}_1}, \mathcal {K}_1\}_e\Big )+O(\varepsilon ^{3})\nonumber \\&{\mathop {=}\limits ^{(7.30),(7.43), (7.22)}} \mathtt {H}_0+\varepsilon ^{2} \Pi _{\mathrm{Ker}(\mathtt {H}_0)}\Big (\mathsf {H}_2^{(1)}+ \frac{1}{2}\{ H_{\mathtt {A}_1}, \{H_{\mathtt {A}_1},\mathsf {H}_0\}_e\}_e\nonumber \\&\quad +\{H_{\mathtt {A}_1}, \mathsf {H}_1\}_e+ \{H_{\mathtt {A}_1},\{ S_1,\mathsf {H}_0\}_e\}_e\Big )+O(\varepsilon ^{3}){.}\nonumber \\&{\mathop {=}\limits ^{(7.23)}} \mathtt {H}_0+\varepsilon ^{2} \Pi _{\mathrm{Ker}(\mathtt {H}_0)}\Big (\frac{1}{2}\{ S_1,\{ S_1, \mathsf {H}_0\}_e\}_{e} + \{S_1,\mathsf {H}_1\}_{e}+\frac{1}{2}\{S_2,\mathsf {H}_0\}_{e} +\mathsf {H}_2+\mathsf {H}_{\mathcal {R}_2}\nonumber \\&\qquad \quad \qquad \quad \qquad \quad + \frac{1}{2}\{ H_{\mathtt {A}_1}, \{H_{\mathtt {A}_1},\mathsf {H}_0\}_e\}_e +\{H_{\mathtt {A}_1}, \mathsf {H}_1\}_e+ \{H_{\mathtt {A}_1},\{ S_1,\mathsf {H}_0\}_e\}_e\Big )+O(\varepsilon ^{3}){.} \end{aligned}$$

Using the Jacobi identity we have

B.15 $$\begin{aligned}&\frac{1}{2}\{ S_1, \{S_1,\mathsf {H}_0\}_e\}_e + \frac{1}{2}\{ H_{\mathtt {A}_1}, \{H_{\mathtt {A}_1},\mathsf {H}_0\}_e\}_e+ \{H_{\mathtt {A}_1},\{ S_1,\mathsf {H}_0\}_e\}_e\nonumber \\&\quad =\frac{1}{2}\{ S_1+H_{\mathtt {A}_1}, \{S_1+H_{\mathtt {A}_1},\mathsf {H}_0\}_e\}_e +\frac{1}{2}\{\mathsf {H}_0,\{S_1,S_1+H_{\mathtt {A}_1}\}\}_e{.} \end{aligned}$$

Moreover, setting $\mathsf {F}_1:=S_1+H_{\mathtt {A}_1}$, we note that

B.16 $$\begin{aligned} \{\mathsf {F}_1,\mathsf {H}_0\}_e= \{H_{\mathtt {A}_1},\mathsf {H}_0\}_e +\{S_1,\mathsf {H}_0\}_e {\mathop {=}\limits ^{(7.22), (7.43),(7.66)}}-\mathsf {H}_1{.} \end{aligned}$$

Since $\Pi _{\mathrm{Ker}(\mathtt {H}_0)}\{S_2,\mathsf {H}_0\}_{e}\equiv 0$, by (B.13), (B.15), and (B.16) we get

B.17 $$\begin{aligned} \Pi ^{d_{w}=2}\Pi _{\mathrm{Ker}(\mathtt {H}_0)} \Big (\mathtt {Z}_0+ \mathcal {K}^{(1)}_2\Big ) =\Pi ^{d_{w}=2} \Pi _{\mathrm{Ker}(\mathtt {H}_0)}\Big ( \frac{1}{2}\{\mathsf {F}_1, \mathsf {H}_1\}_{e} +\mathsf {H}_2+\mathsf {H}_{\mathcal {R}_2} \Big ){.} \end{aligned}$$

Since $\text{ Ker }(H^{(2)})$ is trivial on cubic monomials, we deduce that

B.18 $$\begin{aligned} \mathsf {F}_1 = -\mathrm{ad}^{-1}_{\mathsf {H}_0}(\mathsf {H}_1 ) = \widetilde{F}^{(3,2)} \end{aligned}$$

and this concludes the proof. $\quad \square$

## Appendix C. Technical Lemmata

### C.1 Flows of hyperbolic PDEs

In this subsection we study some properties of the flow of (7.11). We start by studying the time-one flow map of the pseudo differential PDE

C.1 $$\begin{aligned} \partial _{\tau }\Psi ^{\tau }(u)=(J\circ b) \Psi ^{\tau }(u){,}\qquad \Psi ^{0}u=u{,} \end{aligned}$$

and how this differs from $\Phi ^{\tau }$. Proposition 3.1 in [33] guarantees that the flow of (C.1) is the composition of the diffeomorphism of the torus

C.2 $$\begin{aligned}&\mathcal {A}^{\tau }h(\varphi , x):=(1+\tau \beta _x(\varphi , x)) h(\varphi , x+\tau \beta (\varphi , x)){,} \qquad \quad \,\, \varphi \in \mathbb {T}^{\nu }{,}\, x\in \mathbb {T}{,}\nonumber \\&(\mathcal {A}^{\tau })^{-1}h(\varphi , y) :=(1+ \tilde{\beta }_y(\tau ,\varphi , y))\,h(\varphi , y+\tilde{\beta }(\tau , \varphi , y)){,} \quad \varphi \in \mathbb {T}^{\nu }{,}\, y\in \mathbb {T}{,} \end{aligned}$$

where $\tilde{\beta }(\tau ;x,\xi )$ is such that

$$\begin{aligned} x\mapsto y=x+\tau \beta ({\varphi },x) \; \Leftrightarrow \; y\mapsto x=y+\tilde{\beta }(\tau ,{\varphi },x){,} \;\; \tau \in [0,1]{,} \end{aligned}$$

with a pseudo differential operator of order $-1$ up to smoothing remainders belonging to the class $\mathfrak {L}_{\rho ,p}$ (see Definition 2.8 in [33]) for any $\rho \in \mathbb {N}$, $\rho \ge 3$, $p\ge s_0$. For completeness we restate the definition of $\mathfrak {L}_{\rho ,p}$ in Appendix C.2 (see Definition C.5). The class $\mathfrak {L}_{\rho ,p}$ has the property of being closed w.r.t. changes of variables as $\Phi ^\tau$ (see Lemma *B*.10 in [33]). Moreover, in Lemma C.8, we show that $\mathfrak {L}_{\rho ,p}$ is contained into $\mathfrak {C}_{-1}$ which is another class of “tame” operators. Such class is introduced in Definition C.6 and is included in $-1$-tame operators, see Lemma C.10.

We refer to Proposition 3.1, Corollary 3.2 and Appendix *A* in [33] for properties and estimates of $\Psi ^{\tau }$ and $\mathcal {A}^{\tau }$ in (C.2).

#### Lemma C.1

Fix $\rho \ge 3$ and $p\ge s_0$. There exist $\delta \ll 1$, $\sigma _1:=\sigma _1(\rho ,p)$ such that if

C.3 $$\begin{aligned} \Vert \beta \Vert _{s_0+\sigma _1} \le \delta \end{aligned}$$

then the following holds. Let $\Psi ^{\tau }$ be the flow of the system (C.1), then the flow of (7.11) $\Phi ^\tau$ is well defined for $|\tau |\le 1$ and one has $\Phi ^1= \Pi _S^\perp \Psi ^1\Pi _S^\perp \circ (\mathrm {I} +\mathcal {R})$ where $\mathcal {R}$ is an operator with the form (6.16). Moreover $\mathcal {R}$ belongs to $\mathfrak {L}_{\rho ,p}(\mathcal {O})$ and satisfies

C.4 $$\begin{aligned} \mathbb {M}^{\gamma }_{\mathcal {R}}(s, \mathtt {b}) \lesssim _s \Vert \beta \Vert ^{\gamma , \mathcal {O}}_{s+\sigma _1}{,} \qquad \mathbb {M}_{\Delta _{12}\mathcal {R} }(p, \mathtt {b})\lesssim _{p} \Vert \Delta _{12}\beta \Vert _{p+\sigma _1}{.} \end{aligned}$$

#### Proof

Proposition 3.1 in [33] provides $\delta , \sigma _1$ such that if the smallness condition (C.3) is satisfied with such parameters, then the flow $\Psi ^{\tau }$ is well-defined for $|\tau |\le 1$. Let us define $\Upsilon ^{\tau }$ as the flow of the following Cauchy problem

C.5 $$\begin{aligned} \partial _{\tau } \Upsilon ^{\tau } u= -\big (\Psi ^{\tau }_* \mathtt {Z} \big ) \Upsilon ^{\tau } u{,}\qquad \Upsilon ^0 u=u \end{aligned}$$

with

$$\begin{aligned} \mathtt {Z}u&:=(J\circ b) \Pi _S[ u]+\Pi _S(J\circ b) \Pi _S^{\perp }[ u]= \sum _{j\in S} \big (g_j(\tau ), u \big )_{L^2}\, \chi _j(\tau )\\&\quad + \sum _{j\in S} \big (\tilde{g}_j(\tau ), u \big )_{L^2}\, \tilde{\chi }_j(\tau ){,}\\ g_j&=\tilde{\chi }_{j}:=e^{\mathrm {i} j x}{,} \quad \chi _j=J (b(\tau ) \,e^{\mathrm {i} j x}){,} \quad \tilde{g}_j:={\lambda }(j)\Pi _S^{\perp }[b(\tau )\,e^{\mathrm {i} j x}]{.} \end{aligned}$$

Equation (C.5) is well posed on $H^{s}$ since its vector field is finite rank. By the following computations

$$\begin{aligned}&(\partial _{\tau } \Psi ^{\tau }) (\Upsilon ^{\tau } u) +\Psi ^{\tau } (\partial _{\tau } \Upsilon ^{\tau } u)\\&\quad =(J\circ b) \Pi _S^{\perp }[\Psi ^{\tau } (\Upsilon ^{\tau } u)] -\Pi _S[(J\circ b) \Pi _S^{\perp }[\Psi ^{\tau } (\Upsilon ^{\tau } u)]\\&\quad =(J\circ b) \Psi ^{\tau } (\Upsilon ^{\tau } u) -\Big ((J\circ b) \Pi _S[\Psi ^{\tau } (\Upsilon ^{\tau } u)] +\Pi _S[(J\circ b) \Pi _S^{\perp }[\Psi ^{\tau } (\Upsilon ^{\tau } u)]\Big )\, \end{aligned}$$

we have that $\Phi ^{\tau }=\Pi _S^{\perp }\circ \Psi ^{\tau }\circ \Upsilon ^{\tau }$ is well-defined on $H^s$ and solves (7.11).

Now we show that $\Upsilon ^{\tau }-\mathrm {I}$ is of the form (6.16). By Taylor expansion at $\tau =0$ we get

C.6 $$\begin{aligned} \Upsilon ^{\tau } u-u= -\tau \big (\Psi ^{\tau }_* \mathtt {Z} (\Upsilon ^{\tau } u) \big )_{|_{\tau =0}} +\int _0^{\tau }(1-t) \big (\partial _{t t} \Upsilon ^{t}(u)\big )\,dt{.} \end{aligned}$$

Note that

$$\begin{aligned} \Psi ^{\tau }_* \mathtt {Z} (\Upsilon ^{\tau } u)\!=\!\!\sum _{j\in S} \big ((\Phi ^{\tau })^* g_j(\tau ), u \big )_{L^2}\,(\Psi ^{\tau })^{-1}\chi _j(\tau ) \!+\!\!\sum _{j\in S} \big ((\Phi ^{\tau })^*\tilde{g}_j(\tau ), u \big )_{L^2}\,(\Psi ^{\tau })^{-1}\tilde{\chi }_j(\tau ) \end{aligned}$$

has already the form (6.16) and $\big (\Psi ^{\tau }_* \mathtt {Z} (\Upsilon ^{\tau } u)\big )_{|_{\tau =0}}=\mathtt {Z} u$. We denoted by $(\Psi ^{\tau })^*$, $(\Phi ^{\tau })^*$ the flows of the adjoint PDEs

$$\begin{aligned} \partial _{\tau } (\Psi ^{\tau })^{*} u=-b J( (\Psi ^{\tau })^{*} u){,}\qquad \partial _{\tau } (\Phi ^{\tau })^{*} u =-\Pi _S^{\perp }\,b\, J\, \Pi _S^{\perp }[ (\Phi ^{\tau })^{*} u]{.} \end{aligned}$$

We have

$$\begin{aligned} \int _0^{\tau } (1-t)\partial _{t t} \Upsilon ^t(u)\,ds= & {} \sum _{j\in S} \int _0^{\tau }(1-t) \big (\mathtt {g}_j(t), u \big )_{L^2}\, \mathtt {f}_j(t)\,dt\\&+\sum _{j\in S}\int _0^{\tau } (1-t) \big (\tilde{\mathtt {g}}_j(t), u \big )_{L^2}\,\tilde{\mathtt {f}}_j(t)\,dt \end{aligned}$$

where

$$\begin{aligned} \mathtt {g}_j&:= \big (-(\Psi _*^s \mathtt {Z})^* (\Phi ^s)^*-(\Upsilon ^s)^* b\,J ((\Psi ^s)^*)\big )(g_j){,} \\&\quad \mathtt {f}_j:=-(J\circ b) ((\Psi ^{s})^{-1})\chi _j +(\Psi ^s)^{-1}J(b'(s) e^{\mathrm {i} j x}){,}\\ \tilde{\mathtt {g}}_j&:= \big (-(\Psi _*^s \mathtt {Z})^* (\Phi ^s)^*-(\Upsilon ^s)^* b\, J ((\Psi ^s)^*)\big )(\tilde{g}_j) +(\Phi ^s)^*{\lambda }(j)\Pi _S^{\perp }[b'(s) e^{\mathrm {i} j x}]{,}\\&\quad \tilde{\mathtt {f}}_j:=-(J\circ b) ((\Psi ^{s})^{-1})\tilde{\chi }_j{.} \end{aligned}$$

Thus by (C.6), for $\tau =1$ we get

C.7 $$\begin{aligned}&\Upsilon ^1 u-u=\mathcal {R} u{,} \qquad \mathcal {R} u:=\mathcal {R}_1 u+\mathcal {R}_2 u{,}\end{aligned}$$

C.8 $$\begin{aligned}&\mathcal {R}_1 u:=-\mathtt {Z} u{,} \qquad \mathcal {R}_2 u:=\int _0^{1} (1-t)\partial _{t t} \Upsilon ^t(u)\,dt{,} \end{aligned}$$

where $\mathcal {R}_1$ has the finite dimensional form (6.16) and $\mathcal {R}_2$ has the form (6.16). Hence by Lemma C.7 we have

C.9 $$\begin{aligned}&\mathbb {M}^{\gamma }_{\mathcal {R}_1}(s, \mathtt {b}) \lesssim _s \sum _{|j |\le C} \,\,\sup _{\tau \in [0,1]} (\Vert \mathtt {f}_j(\tau )\Vert _s^{\gamma , \mathcal {O}_0} \Vert \mathtt {g}_j(\tau ) \Vert _{s_0}^{\gamma , \mathcal {O}_0} +\Vert \mathtt {f}_j(\tau )\Vert _{s_0}^{\gamma , \mathcal {O}_0} \Vert \mathtt {g}_j(\tau )\Vert _s^{\gamma , \mathcal {O}_0}){,} \nonumber \\ \end{aligned}$$

C.10 $$\begin{aligned}&\mathbb {M}^{\gamma }_{\mathcal {R}_2}(s, \mathtt {b}) \lesssim _s \sum _{|j |\le C} \,\,\sup _{\tau \in [0,1]} (\Vert \tilde{\mathtt {f}}_j(\tau )\Vert _s^{\gamma , \mathcal {O}_0} \Vert \tilde{\mathtt {g}}_j(\tau ) \Vert _{s_0}^{\gamma , \mathcal {O}_0} +\Vert \tilde{\mathtt {f}}_j(\tau )\Vert _{s_0}^{\gamma , \mathcal {O}_0} \Vert \tilde{\mathtt {g}}_j(\tau )\Vert _s^{\gamma , \mathcal {O}_0}){.}\nonumber \\ \end{aligned}$$

By using the estimates in Proposition 3.1 and Corollary 3.2 in [33] we have

C.11 $$\begin{aligned} \Vert \mathtt {f}_j \Vert ^{\gamma , \mathcal {O}_0}_s, \Vert \tilde{ \mathtt {g}_j} \Vert ^{\gamma , \mathcal {O}_0}_s \!\lesssim _s\! \Vert b \Vert ^{\gamma , \mathcal {O}_0}_{s+s_0+2} +\Vert \partial _{\tau } b(\tau ) \Vert ^{\gamma , \mathcal {O}_0}_{s+1}{,}\quad \Vert \tilde{\mathtt {f}_j}\Vert ^{\gamma , \mathcal {O}_0}_s{,} \Vert \mathtt {g}_j \Vert _s^{\gamma , \mathcal {O}_0} \lesssim _s \Vert b \Vert ^{\gamma , \mathcal {O}_0}_{s+s_0+2}{.} \end{aligned}$$

In the same way, the bounds for the variation on the *i*-variable (the second in (C.4)) follows by the estimates on the derivatives of the coefficients $\mathtt {g}_j, \tilde{\mathtt {g}_j}, \chi _j, \tilde{\chi _j}$ whose depend on the variation $\Delta _{1 2}$ of the flows $\Phi ^{\tau }$, $\Psi ^{\tau }$ and their adjoints. We have proved that $\Upsilon ^1u=u+\mathcal {R} u$ and hence $\Phi ^1 u=\Psi ^{1}\circ (\mathrm {I}+\mathcal {R})u$. By (C.9), (C.10) and (C.11) we obtain (C.4). $\quad \square$

The system (7.11) is a Hyperbolic PDE, thus we shall use a version of Egorov Theorem to study how pseudo differential operators change under the flow $\Phi ^{\tau }$. This is the content of Theorem 3.4 in [33] which provides precise estimates for the transformed pseudo differential operators.

The following proposition is the counter-part of Proposition 3.5 in [33]. It describes the structure of an operator like $\mathcal {L}_{\omega }$ conjugated by a flow of a system like (7.11).

#### Proposition C.2

(Conjugation). Let $\mathcal {O}$ be a subset of $\mathbb {R}^\nu$. Fix $\rho \ge 3$, $\alpha \in \mathbb {N}$, $p\ge s_0$ and consider a linear operator

C.12 $$\begin{aligned} \mathcal {L}:=\Pi _S^{\perp }\Big ( \omega \cdot \partial _{\varphi }- J \circ (m+a(\varphi , x)) +\mathcal {Q} \Big ) \end{aligned}$$

where $m=m(\omega )$ is a real constant, $a=a(\omega ,i(\omega ))\in C^\infty (\mathbb {T}^{\nu +1})$ is real valued, both are Lipschitz in $\omega \in \mathcal {O}$ and *a* is Lipschitz in the variable *i*. Moreover $\mathcal {Q}=\mathrm{Op}(\mathtt {q}(\varphi , x, \xi ))+\widehat{\mathcal {Q}}$ with $\widehat{\mathcal {Q}}\in \mathfrak {L}_{\rho ,p}(\mathcal {O})$ and $\mathtt {q}=\mathtt {q}(\omega ,i(\omega ))\in S^{-1}$ satisfying

C.13 $$\begin{aligned} |\mathtt {q} |_{-1, s, \alpha }^{\gamma , \mathcal {O}}&\lesssim _{s, \alpha } \mathtt {k}_1+ \mathtt {k}_2 \Vert f \Vert ^{{\gamma },\mathcal {O}}_{s+\sigma }{,} \end{aligned}$$

C.14 $$\begin{aligned} |\Delta _{12} \mathtt {q} |_{-1, p, \alpha }&\lesssim _{p, \alpha } \mathtt {k}_3(\Vert \Delta _{12} f \Vert _{p+\sigma } +\Vert \Delta _{12} f \Vert _{p+\sigma }\Vert f \Vert _{p+\sigma }){.} \end{aligned}$$

Here $\mathtt {k}_1, \mathtt {k}_2, \mathtt {k}_3,\sigma >0$ are constants depending on $\mathtt {q}$ while $f=f(\omega ,i(\omega ))\in C^\infty (\mathbb {T}^{\nu +1})$, is Lipschitz in $\omega$ and in the variable *i* . There exist $\tilde{\sigma }=\tilde{\sigma }(\rho )>0$ and $\delta _*:=\delta _*(\rho )$ such that, if

C.15 $$\begin{aligned} \Vert \beta \Vert ^{\gamma , \mathcal {O}}_{s_0+\tilde{\sigma }} +\Vert a \Vert ^{\gamma , \mathcal {O}}_{s_0+\tilde{\sigma }} +\mathtt {k}_2 \Vert f\Vert ^{\gamma , \mathcal {O}}_{s_0+\tilde{\sigma }} +\mathtt {k}_1+\mathbb {M}^{{\gamma }}_{\widehat{\mathcal {Q}}}(s_0, \mathtt {b}) \le \delta _*{,} \end{aligned}$$

the following holds for $p+\sigma \le s_0+\tilde{\sigma }$. Consider $\Phi :=\Phi ^{1}$ the flow at time one of the system (7.11), where *b* is defined in (7.10). Then we have

C.16 $$\begin{aligned} \mathcal {L}_+:=\Phi \mathcal {L}\Phi ^{-1} =\Pi _S^{\perp } \Big (\omega \cdot \partial _{\varphi } -J \circ (m+ a_+(\varphi , x))+\mathcal {Q}_+\Big ) \end{aligned}$$

where

C.17 $$\begin{aligned} m+a_+(\varphi , x):= -(\omega \cdot \partial _{\varphi } \tilde{\beta })(\varphi , x+\beta (\varphi , x)) +(m+a(\varphi , x+\beta (\varphi , x))) (1+\tilde{\beta }_x(\varphi , x+\beta (\varphi , x))) \end{aligned}$$

with $\tilde{\beta }$ the function such that $x+\tilde{\beta }(\varphi , x)$ is the inverse of the diffeomorphism of the torus $x\mapsto x+\beta (\varphi , x)$. The operator $\mathcal {Q}_+:=\mathrm{Op}(\mathtt {q}_+(\varphi , x, \xi )) +\widehat{\mathcal {Q}}_+$, with

C.18 $$\begin{aligned} |\mathtt {q}_+ |^{{\gamma }, \mathcal {O}}_{-1, s, \alpha }&\lesssim _{s, \alpha , \rho } \mathtt {k}_1+\mathtt {k}_2 \Vert f\Vert ^{{\gamma }, \mathcal {O}}_{s+\tilde{\sigma }} +\Vert \beta \Vert ^{{\gamma }, \mathcal {O}}_{s+\tilde{\sigma }}+\Vert a \Vert ^{{\gamma }, \mathcal {O}}_{s+\tilde{\sigma }}{,}\nonumber \\ |\Delta _{12} \mathtt {q}_+ |_{-1, p, \alpha }&\lesssim _{p, \alpha , \rho } \mathtt {k}_3(\Vert \Delta _{12} f \Vert _{p+\tilde{\sigma }} +\Vert \Delta _{12} f \Vert _{p+\tilde{\sigma }}\Vert f \Vert _{p+\tilde{\sigma }})\nonumber \\&\quad +\Vert \Delta _{12}\beta \Vert _{p+\tilde{\sigma }} +\Vert \Delta _{12} a \Vert _{p+\tilde{\sigma }} \end{aligned}$$

and $\widehat{\mathcal {Q}}_+\in \mathfrak {L}_{\rho ,p}(\mathcal {O})$ with, for $s_0\le s \le \mathcal {S}$,

C.19 $$\begin{aligned} \mathbb {M}^{{\gamma }}_{\widehat{\mathcal {Q}}_+}(s, \mathtt {b}) \lesssim _{s, \rho } \mathbb {M}^{{\gamma }}_{\widehat{\mathcal {Q}}}(s, \mathtt {b}) + \Vert \beta \Vert ^{{\gamma },\mathcal {O}}_{s+\tilde{\sigma }} +\mathtt {k}_1 +\mathtt {k}_2 \Vert f\Vert ^{{\gamma },\mathcal {O}}_{s+\tilde{\sigma }}+\Vert a\Vert ^{{\gamma },\mathcal {O}}_{s+\tilde{\sigma }}{,} \end{aligned}$$

for any $0\le \mathtt {b}\le \rho -2$ and

C.20 $$\begin{aligned} \mathbb {M}_{\Delta _{12} \widehat{\mathcal {Q}}_+ }(p, \mathtt {b})&\lesssim _{p, \rho } \mathbb {M}_{\Delta _{12} \widehat{\mathcal {Q}}}(p, \mathtt {b})\nonumber \\&\quad +\mathtt {k}_3(\Vert \Delta _{12} f \Vert _{p+\tilde{\sigma }} +\Vert \Delta _{12} f \Vert _{p+\tilde{\sigma }}\Vert f \Vert _{p+\tilde{\sigma }}) +\Vert \Delta _{12}\beta \Vert _{p+\tilde{\sigma }}+\Vert \Delta _{12} a \Vert _{p+\tilde{\sigma }} \end{aligned}$$

for any $$0\le \mathtt {b}\le \rho -3$$.

#### Proof

The strategy is the following.

- We conjugate
  C.21 $$\begin{aligned} \mathcal {L}^0:= \omega \cdot \partial _{\varphi }- J \circ (m+a(\varphi , x)) +\mathcal {Q} \end{aligned}$$
  by the flow $\Psi ^{\tau }$ in (C.1). In order to do this we use Proposition 3.5 in [33].
- The operator $\mathcal {L}^0$ differs from $\mathcal {L}$ by a infinitely regularizing operator of the form (6.16). By using this fact and Lemma C.1 we estimate the difference between $\Phi ^{\tau } \mathcal {L} (\Phi ^{\tau })^{-1}$ and $\Psi ^{\tau } \mathcal {L}^0 (\Psi ^{\tau })^{-1}$.

Let $\Psi ^{\tau }$ be the flow in (C.1). The (C.13)-(C.14) imply that $\mathcal {L}_0$ in (C.21) satisfies the hypotheses of Proposition 3.5 in [33]. Hence, if $y:=x+\beta (\varphi , x)$, then we have

$$\begin{aligned} \mathcal {L}^0_+ h&:=\Psi \mathcal {L}^0 \Psi ^{-1} = \omega \cdot \partial _{\varphi } h-J \{\big (m+a_+(\varphi , x)\big )\,h\} +\mathrm{Op}(\mathtt {q}_+)+\widehat{\mathcal {Q}}_{\star } h{,}\\ m+a_+(\varphi , x)&:= -\omega \cdot \partial _{\varphi }\tilde{\beta }(\varphi , y) +(m+a(\varphi , y))(1+\tilde{\beta }_y(\varphi , y)){.} \end{aligned}$$

In particular $\mathtt {q}_+$ and $\widehat{\mathcal {Q}}_{\star }$ satisfy bounds like (C.18)-(C.20). By Lemma C.1 we have (recall (C.7), (C.8))

$$\begin{aligned}&\widehat{\mathcal {Q}}_{\star \star }:= \Phi \mathcal {L} \Phi ^{-1}-\Pi _S^{\perp } \mathcal {L}^0_+ \Pi _S^{\perp } =\Pi _S^{\perp } \Psi \mathcal {L}^0 \Psi ^{-1}\Pi _S +\Pi _S \Psi \mathcal {L}^0\Psi ^{-1} +\Psi \mathcal {L} \Gamma \Psi ^{-1} \\&\quad +\Psi \mathcal {L} \mathcal {R} \Psi ^{-1}+\Psi \mathcal {R} \mathcal {L} \Gamma \Psi ^{-1} -\Psi \mathcal {L}^0 \Pi _S \Psi ^{-1} -\Psi \Pi _S \mathcal {L} \Pi _S^{\perp } \Psi ^{-1}. \end{aligned}$$

We define the remainder $\widehat{\mathcal {Q}}_+:=\widehat{\mathcal {Q}}_{\star }+\widehat{\mathcal {Q}}_{\star \star }$. To conclude the proof we show that $\widehat{\mathcal {Q}}_{\star \star }$ satisfies the bounds (C.19) and (C.20). We note that

C.22 $$\begin{aligned}&\Pi _S^{\perp } \Psi \mathcal {L}^0 \Psi ^{-1}\Pi _S h =\sum _{j\in S} (h, g_j^{(1)})_{L^2} \chi _j^{(1)}{,} \quad g_j^{(1)}:=e^{\mathrm {i} j x}{,} \quad \chi _j^{(1)}:=\Psi \mathcal {L}^0 \Psi ^{-1}e^{\mathrm {i} j x}{,}\nonumber \\&\Pi _S \Psi \mathcal {L}^0\Psi ^{-1} h =\sum _{j\in S} (h, g_j^{(2)})_{L^2} \chi _j^{(2)}{,} \quad g_j^{(2)}:=\Psi \mathcal {L}^0\Psi ^{-1} e^{\mathrm {i} j x}{,} \quad \chi _j^{(2)}=e^{\mathrm {i} j x}{,}\nonumber \\&\Psi \mathcal {L}^0 \Pi _S \Psi ^{-1} =\sum _{j\in S} (h, g_j^{(3)})_{L^2} \chi _j^{(3)}{,} \quad g_j^{(3)}:=(\Psi ^{-1})^* e^{\mathrm {i} j x}{,} \quad \chi _j^{(3)}:=\Psi \mathcal {L}^0 e^{\mathrm {i} j x}{,}\nonumber \\&\Psi \Pi _S \mathcal {L} \Pi _S^{\perp } \Psi ^{-1} h =\sum _{j\in S} (h, g_j^{(4)})_{L^2} \chi _j^{(4)}{,} \quad g_j^{(4)}:=(\mathcal {L} \Pi _S^{\perp } \Psi ^{-1})^* e^{\mathrm{i} j x}{,} \quad \chi _j^{(4)}:=\Psi e^{\mathrm{i} j x}{.} \end{aligned}$$

Thus by Lemma C.7, Corollary 3.2 in [33] (for the estimates on $\Psi$) and (C.21) we get the bounds (C.19) and (C.20) for the operators (C.22). The bounds on $\Psi \mathcal {L} \Gamma \Psi ^{-1}, \Psi \mathcal {L} \mathcal {R} \Psi ^{-1}, \Psi \mathcal {R} \mathcal {L} \Gamma \Psi ^{-1}$ follow by Proposition 3.1 in [33] and (C.4). $\quad \square$

#### Remark C.3

Assume that the symbols $a, \mathtt {q}$ and the smoothing operator $\widehat{\mathcal {Q}}$ in (C.12) admits an expansion in $\varepsilon$ (actually in degree of homogeneity of $\overline{v}$ in (6.25)) of the form

C.23 $$\begin{aligned} a=\sum _{i=1}^{3}\varepsilon ^{i}a^{(i)}+a^{(\ge 4)}{,}\quad \mathtt {q}=\sum _{i=1}^{3}\varepsilon ^{i}\mathtt {q}^{(i)}+\mathtt {q}^{(\ge 4)}{,}\quad \widehat{\mathcal {Q}}=\sum _{i=1}^{3}\varepsilon ^{i}\widehat{\mathcal {Q}}^{(i)}+\widehat{\mathcal {Q}}^{(\ge 4)}{,} \end{aligned}$$

where $a^{(i)}, \mathtt {q}^{(i)}$ have the form respectively (6.36), (6.34), and $a^{(\ge 4)}$, $\mathtt {q}^{(\ge 4)}$ satisfy estimates like (7.42). The remainders $\widehat{\mathcal {Q}}^{(i)}$ are *almost-diagonal* (see Def. 6.4) and $\widehat{\mathcal {Q}}^{(\ge 4)}$ satisfies estimates like (7.42). Assume also that $\beta$ in (7.10) has an expansion as in (C.23). Then the symbols $a^{+}, \mathtt {q}^{+}$ and the operator $\mathcal {Q}^{+}$ in (C.16) admit the same expansion in $\varepsilon$ as in (C.23). This fact can be deduced by following the proof of Proposition 3.5 in [33]. More precisely one reasons as follows. First of all, by linearity, the conjugate of a sum as in (C.23) is the sum of the conjugates. The conjugate of a $\widehat{\mathcal {Q}}^{(i)}$ in (C.23) under the flow of (7.11) is a smoothing remainder by applying Lemma *B*.10 in [33]. Of course in order to obtain homogeneous terms of degree $\le 3$ in $\varepsilon$ we must Taylor expand the flow, following Remark 7.2 this implies that the remainders are in $\mathfrak {L}_{\rho -3,p}$ (of course since $\rho$ is arbitrary this is not a problem).

The conjugation of the pseudo differential operators $J\circ a(\varphi ,x)$ and $\mathrm{Op}(\mathtt {q}(\varphi , x, \xi ))$ is based on the Egorov Theorem 3.4 in [33]. This is a constructive perturbation scheme so we can Taylor expand up to order three. In conclusion (C.18) holds for each term in the homogeneity expansion, possibly with a larger $\tilde{\sigma }$. On the other hand expanding the remainder $\mathcal {Q}^{+}$ gives an estimate as (C.19) but with $\rho \rightsquigarrow \rho -3$.

#### Remark C.4

We point out that the remainder in Proposition C.2 is of the order of $\beta$, i.e. of the generator of the torus diffeomorphism. This will create problems in fulfilling the smallness conditions in the KAM reducibility scheme of Sect. 7.3, where a term is perturbative if it is small w.r.t. ${\gamma }^{3/2}$ ( and ${\gamma }\ll \varepsilon ^2$ ).

### C.2 Classes of “smoothing” operators

In the first step of our reduction procedure (see Theorem 7.1) we need to work with operators which are pseudo differential up to a remainder in the class $\mathfrak {L}_{\rho ,p}$ defined as follows. This class of smoothing (in space) operators has been introduced in [33].

#### Definition C.5

Fix $s_0\ge (\nu +1)/2$ and $p,\,\mathcal {S}\in \mathbb {N}$ with $s_0\le p< \mathcal {S}$ with possibly $\mathcal {S}=+\infty$. Fix $\rho \in \mathbb {N}$, with $\rho \ge 3$ and consider any subset $\mathcal {O}$ of $\mathbb {R}^\nu$. We denote by $\mathfrak {L}_{\rho , p}=\mathfrak {L}_{\rho , p}(\mathcal {O})$ the set of the linear operators $A=A(\omega ):H^s(\mathbb {T}^{\nu +1})\rightarrow H^{s}(\mathbb {T}^{\nu +1})$, $\omega \in \mathcal {O}$ with the following properties:

- The operator *A* is Lipschitz in $\omega$,
- The operators $\partial _{\varphi }^{\vec {\mathtt {b}}} A$, $[\partial _{\varphi }^{\vec {\mathtt {b}}} A, \partial _x]$, for all $\vec {\mathtt {b}}=(\mathtt {b}_1,\ldots ,\mathtt {b}_\nu )\in \mathbb {N}^{\nu }$ with $0\le |\vec {\mathtt {b}}| \le \rho -2$ have the following properties, for any $s_0\le s\le \mathcal {S}$, with possibly $\mathcal {S}=+\infty$:
  (i) For any $m_{1},m_{2}\in \mathbb {R}$, $m_{1},m_{2}\ge 0$ and $m_{1}+m_{2}=\rho -|\vec {\mathtt {b}}|$ one has that the operator $\langle D_{x}\rangle ^{m_{1}} \partial _{{\varphi }}^{\vec {\mathtt {b}}}A \langle D_{x}\rangle ^{m_{2}}$ is Lip-0-tame according to Definition 2.3 and we set
    C.24 $$\begin{aligned} \mathfrak {M}^{\gamma }_{\partial _{{\varphi }}^{\vec {\mathtt {b}}}A}(-\rho +|\vec {\mathtt {b}}|,s):= \sup _{\begin{array}{c} m_{1}+m_{2}=\rho -|\vec {\mathtt {b}}|\\ m_{1},m_{2}\ge 0 \end{array}}\mathfrak {M}^{\gamma }_{\langle D_{x}\rangle ^{m_{1}} \partial _{{\varphi }}^{\vec {\mathtt {b}}}A \langle D_{x}\rangle ^{m_{2}}}(0,s)\,; \end{aligned}$$
  (ii) For any $m_{1},m_{2}\in \mathbb {R}$, $m_{1},m_{2}\ge 0$ and $m_{1}+m_{2}=\rho - |\vec {\mathtt {b}}|-1$ one has that $\langle D_{x}\rangle ^{m_{1}} [\partial _{{\varphi }}^{\vec {\mathtt {b}}}A,\partial _{x}] \langle D_{x}\rangle ^{m_{2}}$ is Lip-0-tame according to Definition 2.3 and we set
    C.25 $$\begin{aligned} \mathfrak {M}^{\gamma }_{[\partial _{{\varphi }}^{\vec {\mathtt {b}}}A,\partial _{x}]}(-\rho +|\vec {\mathtt {b}}|+1,s):= \sup _{\begin{array}{c} m_{1}+m_{2}=\rho -|\vec {\mathtt {b}}|-1\\ m_{1},m_{2}\ge 0 \end{array}}\mathfrak {M}^{\gamma }_{\langle D_{x}\rangle ^{m_{1}} [\partial _{{\varphi }}^{\vec {\mathtt {b}}}A,\partial _{x}] \langle D_{x}\rangle ^{m_{2}}}(0,s){.} \end{aligned}$$
  We define for $0\le \mathtt {b}\le \rho -2$
  C.26 $$\begin{aligned} \begin{aligned} \mathbb {M}^{\gamma }_{A}(s, \mathtt {b}):=&\max _{0\le |\vec {\mathtt {b}}|\le \mathtt {b}}\max \left( \mathfrak {M}_{\partial _{{\varphi }}^{\vec {\mathtt {b}}}A}^{\gamma }(-\rho +|\vec {\mathtt {b}}|,s), \mathfrak {M}_{\partial _{{\varphi }}^{\vec {\mathtt {b}}}[A,\partial _{x}]}^{\gamma }(-\rho +|\vec {\mathtt {b}}|+1,s)\right) . \end{aligned} \end{aligned}$$
  Assume now that the set $\mathcal {O}$ and the operator *A* depend on $i=i(\omega )$, and are well defined for $\omega \in \mathcal {O}_0\subseteq \Omega _{\varepsilon }$ for all *i* satisfying (6.12). We consider $i_{1}=i_{1}(\omega )$, $i_{2}=i_{2}(\omega )$ and for $\omega \in \mathcal {O}(i_1)\cap \mathcal {O}(i_2)$ we define
  C.27 $$\begin{aligned} \Delta _{12}A:=A(i_1)-A(i_2){.} \end{aligned}$$
  We require the following:
- The operators $\partial _{{\varphi }}^{\vec {\mathtt {b}}}\Delta _{12}A$, $[\partial _{{\varphi }}^{\vec {\mathtt {b}}}\Delta _{12}A ,\partial _{x}]$, for $0\le |\vec {\mathtt {b}}|\le \rho -3$, have the following properties, for any $s_0\le s\le \mathcal {S}$, with possibly $\mathcal {S}=+\infty$:
  (iii) For any $m_{1},m_{2}\in \mathbb {R}$, $m_{1},m_{2}\ge 0$ and $m_{1}+m_{2}=\rho -|\vec {\mathtt {b}}|-1$ one has that $\langle D_{x}\rangle ^{m_{1}} \partial _{{\varphi }}^{\vec {\mathtt {b}}} \Delta _{12}A \langle D_{x}\rangle ^{m_{2}}$ is bounded on $H^{p}$ into itself. More precisely there is a positive constant $\mathfrak {N}_{\partial _{{\varphi }}^{\vec {\mathtt {b}}}\Delta _{12}A }(-\rho +|\vec {\mathtt {b}}|+1,p)$ such that, for any $h\in H^{p}$, we have
    C.28 $$\begin{aligned} \sup _{\begin{array}{c} m_{1}+m_{2}=\rho -|\vec {\mathtt {b}}|-1\\ m_{1},m_{2}\ge 0 \end{array}} \Vert \langle D_{x}\rangle ^{m_{1}} \partial _{{\varphi }}^{\vec {\mathtt {b}}}\Delta _{12}A\langle D_{x}\rangle ^{m_{2}} h\Vert _{p}\le \mathfrak {N}_{\partial _{{\varphi }}^{\vec {\mathtt {b}}}\Delta _{12}A }(-\rho +|\vec {\mathtt {b}}|+1,p)\Vert h\Vert _{p}\,; \end{aligned}$$
  (iv) For any $m_{1},m_{2}\in \mathbb {R}$, $m_{1},m_{2}\ge 0$ and $m_{1}+m_{2}=\rho -|\vec {\mathtt {b}}|-2$ one has that $\langle D_{x}\rangle ^{m_{1}} [\partial _{{\varphi }}^{\vec {\mathtt {b}}} \Delta _{12}A ,\partial _{x}] \langle D_{x}\rangle ^{m_{2}}$ is bounded on $H^{p}$ into itself. More precisely there is a positive constant $\mathfrak {N}_{[\partial _{{\varphi }}^{\vec {\mathtt {b}}}\Delta _{12}A,\partial _{x}] }(-\rho +|\vec {\mathtt {b}}|+2,p)$ such that for any $h\in H^{p}$ one has
    C.29 $$\begin{aligned} \sup _{\begin{array}{c} m_{1}+m_{2}=\rho -|\vec {\mathtt {b}}|-2\\ m_{1},m_{2}\ge 0 \end{array}} \Vert \langle D_{x}\rangle ^{m_{1}}[ \partial _{{\varphi }}^{\vec {\mathtt {b}}}\Delta _{12}A,\partial _{x}]\langle D_{x}\rangle ^{m_{2}} h\Vert _{p}\le \mathfrak {N}_{[\partial _{{\varphi }}^{\vec {\mathtt {b}}}\Delta _{12}A, \partial _{x}] }(-\rho +|\vec {\mathtt {b}}|+2,p)\Vert h\Vert _{p}{.} \end{aligned}$$

We define for $0\le \mathtt {b}\le \rho -3$

C.30 $$\begin{aligned} \mathbb {M}_{\Delta _{12}A }(p, \mathtt {b}):=\max _{0\le |\vec {\mathtt {b}}|\le \mathtt {b}}\max \left( \mathfrak {N}_{\partial _{{\varphi }}^{\vec {\mathtt {b}}}\Delta _{12}A }(-\rho +|\vec {\mathtt {b}}|+1,p), \mathfrak {N}_{\partial _{{\varphi }}^{\vec {\mathtt {b}}}[\Delta _{12}A ,\partial _{x}]}(-\rho +|\vec {\mathtt {b}}|+2,p)\right) {.} \end{aligned}$$

By construction one has that $\mathbb {M}^{\gamma }_{A}(s, \mathtt {b}_1)\le \mathbb {M}^{\gamma }_{A}(s, \mathtt {b}_2)$ if $\mathtt {b}_1\le \mathtt {b}_2\le \rho -2$ and $\mathbb {M}_{\Delta _{12}A }(p, \mathtt {b}_1)\le \mathbb {M}_{\Delta _{12}A }(p, \mathtt {b}_2)$ if $\mathtt {b}_1\le \mathtt {b}_2\le \rho -3$.

We shall also deal with “tame” operators in the following class.

#### Definition C.6

Fix $\mathcal {S}\in \mathbb {N}$ with possibly $\mathcal {S}=+\infty$. Fix $\mathtt {b}=s_0+6\tau +6$ and consider $\mathcal {O}\subseteq \mathbb {R}^\nu$. We denote by $\mathfrak {C}_{-1}:=\mathfrak {C}_{-1}(\mathcal {O})$ the set of the linear operators $A=A(\omega ):H^s(\mathbb {T}^{\nu +1})\rightarrow H^{s}(\mathbb {T}^{\nu +1})$, $\omega \in \mathcal {O}$ which satisfy the following for any $s_0\le s\le \mathcal {S}$:

- $\langle D_x \rangle ^{1/2} A \langle D_x \rangle ^{1/2}$, $\langle D_x \rangle ^{1/2}\partial _{\varphi _m}^{\mathtt {b}} A \langle D_x \rangle ^{1/2}$, $\langle D_x \rangle ^{1/2}[\partial _{\varphi _m}^{\mathtt {b}} A, \partial _x]\langle D_x \rangle ^{1/2}$, for $m=1, \ldots , \nu$, $0\le \mathtt {b}_1\le \mathtt {b}$ are Lip-0-tame operators (see Definition 2.3) and we define
  C.31 $$\begin{aligned} \mathfrak {M}^{{\gamma }}_{\partial _{\varphi _m}^{\mathtt {b}_1}A}(-1, s)&:= \mathfrak {M}^{{\gamma }}_{\langle D_x \rangle ^{1/2} \partial _{\varphi _m}^{\mathtt {b}_1}A \langle D_x \rangle ^{1/2}}(0, s),\nonumber \\ \mathfrak {M}^{{\gamma }}_{\partial _{\varphi _m}^{\mathtt {b}_1}[A, \partial _x]}(-1, s)&:=\mathfrak {M}^{{\gamma }}_{\langle D_x \rangle ^{1/2}\partial _{\varphi _m}^{\mathtt {b}_1} [A, \partial _x]\langle D_x \rangle ^{1/2}}(0, s) {,} \end{aligned}$$
  C.32 $$\begin{aligned} \mathbb {B}^{{\gamma }}_A(s)&:=\max _{\begin{array}{c} 0\le \mathtt {b}_1\le \mathtt {b}\\ m=1,\ldots , \nu \end{array}} \max \Big (\mathfrak {M}^{{\gamma }}_{\partial _{\varphi _m}^{\mathtt {b}_1}A}(-1, s), \mathfrak {M}^{{\gamma }}_{\partial _{\varphi _m}^{\mathtt {b}_1}[A, \partial _x]}(-1, s) \Big ){.} \end{aligned}$$
  Assume now that the set $\mathcal {O}$ and the operator *A* depend on $i=i(\omega )$, and are well defined for $\omega \in \mathcal {O}\subseteq \mathbb {R}^\nu$ for all *i* satisfying (6.12). We consider $i_{1}=i_{1}(\omega )$, $i_{2}=i_{2}(\omega )$ and for $\omega \in \mathcal {O}(i_1)\cap \mathcal {O}(i_2)$ let $\Delta _{12}A$ as in (C.27). We require the following:
- $\langle D_x \rangle ^{1/2} \partial _{\varphi _m}^{\mathtt {b}_1} \Delta _{12} A \langle D_x \rangle ^{1/2}$, $\langle D_x \rangle ^{1/2}[\partial _{\varphi _m}^{\mathtt {b}_1} \Delta _{12} A, \partial _x]\langle D_x \rangle ^{1/2}$ for $m=1, \ldots , \nu$, $0\le \mathtt {b}_1\le \mathtt {b}$ are bounded operators on $H^{s_0}$ into itself. More precisely there are positive constants $\mathfrak {N}_{\partial _{\varphi _m}^{\mathtt {b}_1}\Delta _{12} A}(-1, s_0)$, and $\mathfrak {N}_{[\partial _{\varphi _m}^{\mathtt {b}_1}\Delta _{12} A,\partial _{x}]}(-1, s_0)$ such that, for any $h\in H^{s_0}$, we have
  C.33 $$\begin{aligned} \Vert \langle D_x \rangle ^{1/2} \partial _{\varphi _m}^{\mathtt {b}_1} \Delta _{12} A \langle D_x \rangle ^{1/2}h\Vert _{s_0}&\le \mathfrak {N}_{\partial _{\varphi _m}^{\mathtt {b}_1}\Delta _{12} A}(-1, s_0)\Vert h\Vert _{s_0}{,}\nonumber \\ \Vert \langle D_x \rangle ^{1/2} [\partial _{\varphi _m}^{\mathtt {b}_1} \Delta _{12} A,\partial _{x}] \langle D_x \rangle ^{1/2}h\Vert _{s_0}&\le \mathfrak {N}_{[\partial _{\varphi _m}^{\mathtt {b}_1}\Delta _{12} A,\partial _{x}]}(-1, s_0)\Vert h\Vert _{s_0}{.} \end{aligned}$$
  We define
  C.34 $$\begin{aligned} \mathbb {B}_{\Delta _{12} A}(s_0):= \max _{\begin{array}{c} 0\le \mathtt {b}_1\le \mathtt {b}\\ m=1,\ldots , \nu \end{array}} \max \Big (\mathfrak {N}_{\partial _{\varphi _m}^{\mathtt {b}_1}\Delta _{12}A}(-1, s_0), \mathfrak {N}_{\partial _{\varphi _m}^{\mathtt {b}_1}[\Delta _{12}A, \partial _x]}(-1, s_0) \Big ){.} \end{aligned}$$

The next Lemma shows that the finite rank operators of the form (6.16) are in $\mathfrak {L}_{\rho , p}$.

#### Lemma C.7

Fix $\rho \ge 3$. Let $\mathcal {R}$ be an operator of the form (6.16), where the functions $g_j(\tau ), \chi _j(\tau )$ belong to $H^s$ for $\tau \in [0, 1]$ and depend in a Lipschitz way on the parameter $\omega \in \mathcal {O}\subset \mathbb {R}^{\nu }$. Then there exists $\sigma _1=\sigma _1(\rho )>0$ such that $\mathcal {R}$ belongs to $\mathfrak {L}_{\rho , p}$ and

C.35 $$\begin{aligned}&\mathbb {M}^{{\gamma }}_{\mathcal {R}}(s, \mathtt {b})\lesssim _s \sum _{|j |\le C} \,\,\sup _{\tau \in [0,1]} (\Vert \chi _j(\tau )\Vert _{s+\sigma _1}^{\gamma , \mathcal {O}} \Vert g_j \Vert _{s_0+\sigma _1}^{\gamma , \mathcal {O}} +\Vert \chi _j(\tau )\Vert _{s_0+\sigma _1}^{\gamma , \mathcal {O}} \Vert g_j(\tau )\Vert _{s+\sigma _1}^{\gamma , \mathcal {O}}){,} \end{aligned}$$

C.36 $$\begin{aligned}&\mathbb {M}_{\Delta _{12} \mathcal {R} }(p, \mathtt {b}) \le _{p} \sum _{|j |\le C} \,\,\sup _{\tau \in [0,1]} \Big ( \Vert \Delta _{12} \chi _j(\tau ) \Vert _{p+\sigma _1} \Vert g_j \Vert _{p+\sigma _1}+ \Vert \chi _j(\tau ) \Vert _{p+\sigma _1}\Vert \Delta _{12} g_j \Vert _{p+\sigma _1} \Big ){.}\nonumber \\ \end{aligned}$$

#### Proof

The Lemma follows by reasoning as in the proof of Lemma B.2 in [33] and using the explicit formula (6.16). $\quad \square$

We conclude this section by showing the connection between the class $\mathfrak {L}_{\rho ,p}$ and the class $\mathfrak {C}_{-1}$ in Definition C.6.

#### Lemma C.8

Consider $\mathtt {b}\in \mathbb {N}$ and $\rho \in \mathbb {N}$ with $\rho \ge \mathtt {b}+3$. The following holds.

(i) If $A\in \mathfrak {L}_{\rho ,p}$ (see Definition C.5) then $A\in \mathfrak {C}_{-1}$ (see Definition C.6) with
  C.37 $$\begin{aligned} \mathbb {B}^{\gamma }_{A}(s,\mathtt {b}) \le _{\rho ,s}\mathbb {M}^{\gamma }_{A}(s,\rho -2){,} \qquad \mathbb {B}_{\Delta _{12}A }(p,\mathtt {b}) \le _{\rho ,p}\mathbb {M}_{\Delta _{12}A }(p,\rho -3){.} \end{aligned}$$
(ii) Consider a symbol $a=a(\omega , i(\omega ))$ in $S^{m}$ with $m\le -1$ depending on $\omega \in \mathcal {O}_0\subset \mathbb {R}^{\nu }$ in a Lipschitz way and on *i* in a Lipschitz way and let $A:=\mathrm{Op}(a(x,\xi ))$. Then one has that $A\in \mathfrak {C}_{-1}$ with
  C.38 $$\begin{aligned} \mathbb {B}^{\gamma }_{A}(s,\mathtt {b}) \le _{s}|a|^{{\gamma },\mathcal {O}_0}_{m,s+\mathtt {b}+2, 0}{,} \quad \mathbb {B}_{\Delta _{12} A}(p,\mathtt {b}) \le _{s}|\Delta _{12} a|_{m,p+\mathtt {b}+3, 0}. \end{aligned}$$
(iii) Let $A,B\in \mathfrak {C}_{-1}$. Then $A\circ B\in \mathfrak {C}_{-1}$ with
  C.39 $$\begin{aligned} \mathbb {B}^{\gamma }_{A\circ B}(s,\mathtt {b})&\le _{s} \mathbb {B}^{\gamma }_{A}(s,\mathtt {b})\mathbb {B}^{\gamma }_{A}(s_0,\mathtt {b})+ \mathbb {B}^{\gamma }_{A}(s_0,\mathtt {b})\mathbb {B}^{\gamma }_{A}(s,\mathtt {b}) \end{aligned}$$
  C.40 $$\begin{aligned} \mathbb {B}_{\Delta _{12} (A\circ B) }(p, \mathtt {b})&\le _{p, \rho } \mathbb {B}_{\Delta _{12} A }(p,\mathtt {b})\mathbb {B}_B(p,\mathtt {b}){,} +\mathbb {B}_{\Delta _{12} B }(p,\mathtt {b})\mathbb {B}_A(p,\mathtt {b}){.} \end{aligned}$$

#### Proof

Let us check item (*i*). The fact that $\langle D_{x}\rangle ^{1/2}A\langle D_x \rangle ^{1/2}$ is Lip-0-tame follows by (C.24) since $\rho \ge 1$. Indeed $\langle D_{x}\rangle ^{-\rho +1}$ is bounded in *x* and for any $h\in H^{s}$

$$\begin{aligned} \Vert \langle D_{x}\rangle ^{\frac{1}{2}}A\langle D_x \rangle ^{\frac{1}{2}}h\Vert _{s}^{{\gamma },\mathcal {O}_0}&\le \Vert \langle D_{x}\rangle ^{-\rho +1}\big (\langle D_{x}\rangle ^{\rho -\frac{1}{2}}A \langle D_x \rangle ^{\frac{1}{2}}\big ) h\Vert _{s}^{{\gamma },\mathcal {O}_0}\\&\le _{s}\mathfrak {M}^{\gamma }_{A}(-\rho ,s)\Vert h\Vert ^{\gamma ,\mathcal {O}_0}_{s_0}+ \mathfrak {M}^{\gamma }_{A}(-\rho ,s_0)\Vert h\Vert ^{\gamma ,\mathcal {O}_0}_{s}. \end{aligned}$$

By studying the tameness constant of the operators $\partial _{\varphi }^{\vec {\mathtt {b}}} A, [A, \partial _x], [\partial _{\varphi }^{\vec {\mathtt {b}}} A, \partial _x] \Delta _{12}A ,\partial _{{\varphi }}^{\vec {\mathtt {b}}}\Delta _{12}A , [\Delta _{12}A ,\partial _{x}]$, $[\partial _{{\varphi }}^{\vec {\mathtt {b}}}\Delta _{12}A ,\partial _{x}]$ for $\vec {\mathtt {b}}\in \mathbb {N}^{\nu }$, $|\vec {\mathtt {b}}|=\mathtt {b}$, following the same reasoning as above, one gets the (C.37).

In order to prove item (*ii*) one can follow almost word by word the proof of Lemma *A*.4 in [33].

Let us check (C.39). Let $\mathtt {b}\in \mathbb {N}$ and consider $0\le \mathtt {b}_1\le \mathtt {b}$, $m=1, \ldots , \nu$. One has

C.41 $$\begin{aligned} \langle D_{x}\rangle ^{\frac{1}{2}}\partial _{{\varphi }_m}^{\mathtt {b}_1}(A\circ B) \langle D_{x}\rangle ^{\frac{1}{2}} =\sum _{\mathtt {c}_1+\mathtt {c}_2=\mathtt {b}_1}C(\mathtt {c}_1, \mathtt {c}_2) \langle D_{x}\rangle ^{\frac{1}{2}}(\partial _{{\varphi }_m}^{\mathtt {c}_1}A) (\partial _{{\varphi }_m}^{\mathtt {c}_2}B)\langle D_{x}\rangle ^{\frac{1}{2}}{.} \end{aligned}$$

We show that each summand in (C.41) is a Lip-0-tame operator. We have for $h\in H^{s}$

C.42 $$\begin{aligned}&\Vert \langle D_{x}\rangle ^{\frac{1}{2}} (\partial _{{\varphi }_m}^{\mathtt {c}_1}A) (\partial _{{\varphi }_m}^{\mathtt {c}_2}B)\langle D_{x}\rangle ^{\frac{1}{2}}h \Vert _{s}^{\gamma ,\mathcal {O}_0} \nonumber \\&\quad \le \Vert \langle D_{x}\rangle ^{\frac{1}{2}} (\partial _{{\varphi }_m}^{\mathtt {c}_1}A)\langle D_{x}\rangle ^{\frac{1}{2}} \langle D_{x}\rangle ^{-1} \langle D_{x}\rangle ^{\frac{1}{2}} (\partial _{{\varphi }_m}^{\mathtt {c}_2}B)\langle D_{x}\rangle ^{\frac{1}{2}}h \Vert _{s}^{\gamma ,\mathcal {O}_0}\nonumber \\&\quad \le _{s} (\mathbb {B}^{\gamma }_{A}(s,\mathtt {b}) \mathbb {B}^{\gamma }_{B}(s_0,\mathtt {b}) +\mathbb {B}^{\gamma }_{A}(s_0,\mathtt {b})\mathbb {B}^{\gamma }_{B}(s,\mathtt {b}))\Vert h\Vert _{s_0}^{\gamma ,\mathcal {O}_0} +\mathbb {B}^{\gamma }_{A}(s_0,\mathtt {b}) \mathbb {B}^{\gamma }_{B}(s_0,\mathtt {b})\Vert h\Vert _{s}^{\gamma ,\mathcal {O}_0}{.} \end{aligned}$$

In (C.42) we used the fact that $\langle D_{x}\rangle ^{\frac{1}{2}} (\partial _{{\varphi }_m}^{\mathtt {c}_1}A)\langle D_{x}\rangle ^{\frac{1}{2}}$ and $\langle D_{x}\rangle ^{\frac{1}{2}} (\partial _{{\varphi }_m}^{\mathtt {c}_2}B)\langle D_{x}\rangle ^{\frac{1}{2}}$ are 0-tame by hypothesis (see Definition (C.6)). This proves (C.39) for the operators $A\circ B$ and $\partial _{{\varphi }_m}^{\mathtt {b}_1}(A\circ B)$ for any $0\le \mathtt {b}_1\le \mathtt {b}$, $m=1, \ldots , \nu$. One concludes the proof of (C.39) and (C.40) followings the same ideas used above. For further details we refer to the proof of Lemma *B*.1 in [33]. $\quad \square$

#### Lemma C.9

Let $X,Y\in \mathfrak {C}_{-1}$ then $\mathrm {ad}^k_{X}[Y]\in \mathfrak {C}_{-1}$ for any $k\ge 1$ (recall (7.68)). Moreover for any $k\ge 1$ we have

C.43 $$\begin{aligned} \mathbb {B}^{{\gamma }}_{\mathrm {ad}^k_{X}[Y]}(s) \lesssim _s \mathbb {B}_{X}^{{\gamma }}(s) \big (\mathbb {B}_{X}^{{\gamma }}(s_0)\big )^{k-1}\mathbb {B}^{{\gamma }}_Y(s_0) +\big (\mathbb {B}_{X}^{{\gamma }}(s_0)\big )^k \mathbb {B}^{{\gamma }}_Y(s){.} \end{aligned}$$

Moreover if *X*, *Y* depend on some parameter *i* we have

C.44 $$\begin{aligned} \mathbb {B}_{\Delta _{12}\mathrm {ad}^k_{X}[Y]}(s_0)\lesssim \sum _{j_1+j_2=k-1} \mathbb {B}_{X(i_2)}^{j_1}(s_0)\,\mathbb {B}_{\Delta _{12} X}(s_0)\,\mathbb {B}_{X(i_1)}^{j_2}(s_0)\,\mathbb {B}_{Y(i_1)}(s_0)+\mathbb {B}_{X(i_2)}^k \mathbb {B}_{\Delta _{12} Y}(s_0). \end{aligned}$$

#### Proof

It follows by using the formula

$$\begin{aligned} \Delta _{12} \mathrm {ad}_X^k[Y]=\sum _{j_1+j_2=k-1} \mathrm {ad}_{X(i_2)}^{j_1}\,\mathrm {ad}_{\Delta _{12} X}\,\mathrm {ad}^{j_2}_{X(i_1)}[Y(i_1)]+\mathrm {ad}_{X(i_2)}^{k}[\Delta _{12} Y] \end{aligned}$$

and applying iteratively the estimates of Lemma C.8. $\quad \square$

#### Lemma C.10

Let $A\in \mathfrak {C}_{-1}$ then *A* is a Lip-$-1$-modulo tame operator according to Definition 2.7. Moreover

C.45 $$\begin{aligned}&\mathfrak {M}^{\sharp , \gamma ^{3/2}}_A( s)\le \max _{m=1, \ldots , \nu } \mathfrak {M}^{\gamma ^{3/2}}_{\partial _{\varphi _m}^{s_0} [A, \partial _x]}(-1,s){,} \quad \mathfrak {M}^{\sharp , \gamma ^{3/2}}_A( s, \mathtt {b}_0)\le \max _{m=1,\ldots , \nu } \mathfrak {M}^{\gamma ^{3/2}}_{\partial _{\varphi _m}^{s_0+\mathtt {b}_0} [A, \partial _x]}(-1,s){,} \end{aligned}$$

C.46 $$\begin{aligned}&\Vert {\langle D_x\rangle }^{1/2}\underline{\Delta _{12} A}{\langle D_x\rangle }^{1/2}\Vert _{\mathcal L(H^{s_0})}, \,\, \Vert {\langle D_x\rangle }^{1/2}\underline{\Delta _{12} \langle \partial _{{\varphi }}\rangle ^{{\mathtt {b}_0}} A } {\langle D_x\rangle }^{1/2}\Vert _{\mathcal L(H^{s_0})} \le \mathbb {B}_{\Delta _{12} A}(s_0, \mathtt {b}_0){.} \end{aligned}$$

#### Proof

It follows arguing as in the proof of Lemma *A*.4 in Appendix *A* of [33] using the definition of $\mathfrak {C}_{-1}$ in Definition C.6. $\quad \square$

### C.3 Linear Birkhoff normal form

The next lemma regards almost diagonal vector fields which belong to $\mathfrak {C}_{-1}$.

#### Lemma C.11

Let $X_A:=J A\in \mathfrak {C}_{-1}$ with *A* almost diagonal in the sense of Definition 6.4 . Then $|A_{j, l}^{j', l'} |\le C\langle j,j'\rangle ^{-2}$ for some constant $C>0$.

#### Proof

The operator $B:=\langle D_x \rangle ^{1/2} J A \langle D_x \rangle ^{1/2}$ belongs to $\mathcal {L}(H^s)$ for any *s*. Then $|B_{j, l}^{j', l'} |\le C$ for all $j, j'\in \mathbb {Z}$, $l, l'\in \mathbb {Z}^{\nu }$. The thesis follows by the fact that

$$\begin{aligned} |B_{j, l}^{j', l'} |=|A_{j, l}^{j', l'} |\langle j \rangle ^{1/2}\langle j' \rangle ^{1/2}|{\lambda }(j)|\gtrsim |A_{j, l}^{j', l'} |\langle j \rangle ^{1/2} \langle j' \rangle ^{1/2}|j |\end{aligned}$$

and $|j |\gtrsim \langle j \rangle ^{1/2}\langle j' \rangle ^{1/2}$, since *A* is almost diagonal. $\quad \square$

We now study the flows generated by operators belonging to the class $\mathfrak {C}_{-1}$ given in Definition C.6.

In Sect. 7.2 we look for symplectic changes of variable $\Upsilon _i:H^s_{S^{\perp }}(\mathbb {T}^{\nu +1})\rightarrow H^s_{S^{\perp }}(\mathbb {T}^{\nu +1})$, $i=1,2, 3$, that are the time-1 flow of quadratic Hamiltonians

C.47 $$\begin{aligned} H_{\mathtt {A}_i}(u):=\varepsilon \sum _{j, j'\in S^c} (\mathtt {A}_i)_j^{j'}(\varphi )\,u_{j'}\,\overline{u}_{j}{,} \end{aligned}$$

where $\mathtt {A}_i(\varphi )$ is a self-adjoint operator $\forall \varphi \in \mathbb {T}^{\nu }$ and thus

C.48 $$\begin{aligned} \Upsilon _i:=\exp (\varepsilon \, J \mathtt {A}_i) =\mathrm {I}_{H_S^{\perp }}+\varepsilon \, J \mathtt {A}_i +\varepsilon ^2 \frac{(J \mathtt {A}_i)^2}{2} +\varepsilon ^3 R_i{,} \quad R_i:=\sum _{k\ge 3} \frac{\varepsilon ^{k-3}}{k!}\,(J \mathtt {A}_i)^k{.} \end{aligned}$$

Given linear operators $\mathtt {B}_i$, $i=1,2,3$ define the matrices $\mathtt {A}_i$, $i=1,2,3$ as

C.49 $$\begin{aligned} (\mathtt {A}_i)_{j, l}^{j', l'}&=(\mathtt {A}_i)_{j}^{j'}(l-l') =-\frac{(\mathtt {B}_i)_{j}^{j'}(l-l')}{\delta _{l j j'}}{,} \nonumber \\&\quad \delta _{l j j'}\ne 0{,}\quad |j-j'|\le 2\,i\,\overline{\jmath }_1{,} \,\,\,\,|l-l'|\le i, \quad i=1, 2{,} \end{aligned}$$

C.50 $$\begin{aligned} (\mathtt {A}_3)_{j, l}^{j', l'}&=(\mathtt {A}_3)_{j}^{j'}(l-l') =-\frac{(\mathtt {B}_3)_{j}^{j'}(l-l')}{\delta ^*_{l j j'}}{,}\nonumber \\&\quad \delta ^*_{l j j'}\ne 0{,}\quad |j-j'|\le 6\,\overline{\jmath }_1{,} \,\,\,\,|l-l'|\le 3{,} \end{aligned}$$

where, recall (1.8), (4.6),(5.6),

C.51 $$\begin{aligned} \delta _{\ell j j'}:=\overline{\omega }\cdot \ell +{\lambda }(j)-{\lambda }(j'), \quad \delta _{\ell j j'}^*:=\delta _{\ell j j'} +\varepsilon ^2 ( \mathbb {A}\xi \cdot \ell +{\lambda }(j')\mathfrak {l}_{j'}-{\lambda }(j)\mathfrak {l}_j). \end{aligned}$$

#### Lemma C.12

Let $j, j'\in S^c$, $j\ne j'$. If $\sum _{i=1}^{\nu } \overline{\jmath }_i\,\ell _i+j-j'=0$, $0<|\ell |\le 2$, $\delta _{\ell j j'}\ne 0$, where $\delta _{\ell j j'}$ are given in (C.51), then there exists a constant *C* depending on the set *S* such that $|\delta _{\ell j j'} |\ge C$.

#### Proof

If $|\ell |=1$ we have by the preservation of momentum

$$\begin{aligned} \delta _{\ell , j, j'}={\lambda }(j-j')-{\lambda }(j)+{\lambda }(j')=\frac{3 j j'(j-j')[3+j j'+(j-j')^2]}{(1+j^2)(1+j'^2)(1+(j-j')^2)}{.} \end{aligned}$$

It is easy to verify that (recall that $|j-j'|\le 2 \overline{\jmath }_1$)

$$\begin{aligned}&|j\,\, j' ||j-j'||3+j j'+(j-j')^2 |\ge |j\,\, j' |^2, \quad (1+j^2)(1+j'^2)(1+(j-j')^2)\\&\quad \le K |j\,\, j' |^2\overline{\jmath }_1^2{.} \end{aligned}$$

Which implies the thesis for $|\ell |=1$. Now suppose $|\ell |=2$ and consider $j_1, j_2\in S$. We can write $\delta _{\ell j j'}={\lambda }(j_1)+{\lambda }(j_2)+{\lambda }(j)-{\lambda }(j')$ and by the conservation of momentum

C.52 $$\begin{aligned} {\lambda }(j_1)+{\lambda }(j_2)+{\lambda }(j)-{\lambda }(j')=(j_1+j_2)(j_1+j)(j_2+j) \,P(j_1, j_2, j) \end{aligned}$$

and *P* is the rational function

C.53 $$\begin{aligned} P(x, y, z):=\frac{3+x^2+y^2+z^2+xy+xz+ y z+x y z(x+y+z)}{(1+x^2)(1+y^2)(1+z^2)(1+(x+y+z)^2)}{.} \end{aligned}$$

If $|j |> N$, where $N=N(S)$ is a large constant to be fixed and which depends on the set *S*, then

$$\begin{aligned} |(j_1+j_2) (j_1+j) (j_2+j)|\ge C_1\,j^2 \end{aligned}$$

for some constant $C_1:=C_1(N)>0$ (possibly small), provided that *N* is large enough. Moreover

$$\begin{aligned}&|(1+j_1 j_2) j^2+(j_1^2 j_2+j_1 j_2^2) j+3+j_1^2+j_2^2 |\ge C_2\, j^2{,} \\&\quad |(1+j_1^2)(1+j_2^2)(1+j^2)(1+(j_1+j_2+j)^2)|\le C_3\, j^4 \end{aligned}$$

for some small constant $C_2:=C_2(N)>0$, provided that *N* is large enough, and some big constant $C_3:=C_3(S)>0$. Thus $|{\lambda }(j_1)+{\lambda }(j_2)+{\lambda }(j)-{\lambda }(j') |\ge C_1 C_2 C_{3}^{-1}>0$.

Now consider $|j |\le N$. Then we have

$$\begin{aligned}&|(j_1+j_2) (j_1+j) (j_2+j)||(1+j_1 j_2) j^2+(j_1^2 j_2+j_1 j_2^2) j+3+j_1^2+j_2^2 |\ge M_1\\&|(1+j_1^2)(1+j_2^2)(1+j^2)(1+(j_1+j_2+j)^2|\le M_2 \end{aligned}$$

for some constant $M_1, M_2>0$ depending on *S*. Set $C_4:=M_1/ M_2$.

Therefore $|{\lambda }(j_1)+{\lambda }(j_2)+{\lambda }(j)-{\lambda }(j')|\ge C_4>0$. At the end we choose $C\ge \max \{ C_4, C_1 C_2/ C_3 \}$. $\quad \square$

#### Lemma C.13

Assume that $\mathtt {B}_{i}$, $i=1,2,3$ are such that $J\mathtt {B}_{i}\in \mathfrak {C}_{-1}$ and that they are *almost diagonal* (see Definition 6.4). Then, for any $\omega \in \mathcal {G}^{(1)}_0$ (see (5.5)), the following holds true:

(*i*) The linear vector fields $X_{\mathtt {A}_i}:=J \mathtt {A}_i$, with $\mathtt {A}_i$ defined as in (C.49),(C.50), belongs to the class $\mathfrak {C}_{-1}$, in particular it satisfies the following:

C.54 $$\begin{aligned} \mathbb {B}_{\varepsilon ^i X_{\mathtt {A}_i}}^{{\gamma }}(s) \le C(s) \varepsilon ^i \quad i=1,2{,} \qquad \mathbb {B}_{\varepsilon ^i X_{\mathtt {A}_3}}^{{\gamma }}(s) \le C(s) \varepsilon ^3{\gamma }^{-1}{,}\qquad \forall s\ge s_0{.} \end{aligned}$$

Note that $X_{\mathtt {A}_i}$ does not depend on $i(\omega )$.

(*ii*) The transformation $\Upsilon _i:H^s(\mathbb {T}^{\nu +1})\rightarrow H^s(\mathbb {T}^{\nu +1})$, $i=1,2,3$ defined in (C.48) is invertible and satisfies, for any $u=u(\omega )\in H^s$ Lipschitz in $\omega \in \mathcal {O}_{\infty }^{2{\gamma }}$,

C.55 $$\begin{aligned} \Vert (\Upsilon _i^{\pm 1}-\mathrm {I}) u \Vert _s^{{\gamma }, \mathcal {O}_{\infty }^{2{\gamma }}}&\lesssim \varepsilon ^i C(s_0) \Vert u \Vert ^{{\gamma }, \mathcal {O}_{\infty }^{2{\gamma }}}_s + \varepsilon ^i C(s) \Vert u \Vert ^{{\gamma }, \mathcal {O}_{\infty }^{2{\gamma }}}_{s_0}{,} \quad i=1,2{,} \end{aligned}$$

C.56 $$\begin{aligned} \Vert (\Upsilon _3^{\pm 1}-\mathrm {I}) u \Vert _s^{{\gamma }, \mathcal {O}_{\infty }^{2{\gamma }}}&\lesssim \varepsilon ^3\gamma ^{-1} C(s_0) \Vert u \Vert ^{{\gamma }, \mathcal {O}_{\infty }^{2{\gamma }}}_s + \varepsilon ^3\gamma ^{-1} C(s) \Vert u \Vert ^{{\gamma }, \mathcal {O}_{\infty }^{2{\gamma }}}_{s_0}{.} \end{aligned}$$

#### Proof

First of all notice that, by Lemmata C.11, C.12 and the fact that $\omega \in \mathcal {G}_0^{(1)}$ (see (5.5)) we will have

C.57 $$\begin{aligned} |(\mathtt {A}_i)_{j, l}^{j', l'} |\le \frac{C}{\langle j, j'\rangle ^2} \quad i=1,2, \qquad |(\mathtt {A}_3)_{j, l}^{j', l'} |\le \frac{C\gamma }{\langle j, j'\rangle ^2}{,} \qquad \forall j, j'\in S^c,\,\, l, l'\in \mathbb {Z}^{\nu } \end{aligned}$$

for some constant $C>0$ depending on the set *S*.

*Proof of item (i).* First we note that that $B:=\langle D_x \rangle ^{1/2} J \mathtt {A}_i \langle D_x \rangle ^{1/2}$ maps $H^s$ to itself for all $s\ge 0$. Indeed it is sufficient to exploit the fact that the matrix entries $B_j^{j'}(l-l')$ are uniformly bounded by a constant and *B* is almost diagonal. The matrix elements of $\partial _{\varphi _m}^{\mathtt {b}} X_{\mathtt {A}_1}$, $[X_{\mathtt {A}_1}, \partial _x]$, $[\partial _{\varphi _m}^{\mathtt {b}} X_{\mathtt {A}_1}, \partial _x]$ are respectively

$$\begin{aligned}&\langle \ell _m-\ell '_m \rangle ^{\mathtt {b}} {\lambda }(j) (\mathtt {A}_1)_{j}^{j'}(\ell -\ell '){,} \;\; (j-j') {\lambda }(j) (\mathtt {A}_1)_{j}^{j'}(\ell -\ell '){,}\\&\quad \langle \ell _m-\ell '_m \rangle ^{\mathtt {b}}(j-j') {\lambda }(j) (\mathtt {A}_1)_{j}^{j'}(\ell -\ell '){.} \end{aligned}$$

Note that by the definition of $\mathtt {A}_1$ in (C.49)

$$\begin{aligned} \langle \ell _m-\ell '_m \rangle ^{\mathtt {b}}, |j-j'|\le C \end{aligned}$$

for some constant *C* depending on the set *S*. Thus arguing as above one can easily prove that $\partial _{\varphi _m}^{\mathtt {b}} X_{\mathtt {A}_1}$, $[X_{\mathtt {A}_1}, \partial _x]$, $[\partial _{\varphi _m}^{\mathtt {b}} X_{\mathtt {A}_1}, \partial _x]$ are $-1$-Lip-tame operators. This concludes the proof of the (C.54)

*Proof of item (ii).* By (C.48) we have

C.58 $$\begin{aligned} (\Upsilon _i-\mathrm {I})u =\sum _{k\ge 1} \frac{\varepsilon ^{i k} X_{\mathtt {A}_i}^k u}{k!}{,} \qquad (\Upsilon _1^{-1}-\mathrm {I})u =\sum _{k\ge 1} (-1)^k\frac{\varepsilon ^{i k} X_{\mathtt {A}_i}^k u}{k!}{.} \end{aligned}$$

By using iteratively the property (*iii*) of Lemma C.8 and item (*i*) we have that

$$\begin{aligned} \Vert X^k_{\mathtt {A}_i} u \Vert _s^{{\gamma }, \mathcal {O}_{\infty }^{2{\gamma }}} \le \mathbb {B}^{{\gamma }}_{X_{\mathtt {A}_i}}(s) (\mathbb {B}^{{\gamma }}_{X_{\mathtt {A}_i}}(s_0) )^{k-1} \Vert u \Vert _{s_0}^{{\gamma }, \mathcal {O}_{\infty }^{2{\gamma }}} +(\mathbb {B}^{{\gamma }}_{X_{\mathtt {A}_i}}(s_0) )^{k} \Vert u \Vert _s^{{\gamma }, \mathcal {O}_{\infty }^{2{\gamma }}}{.} \end{aligned}$$

By using this relation to estimate the Lip-Sobolev norm of (C.58) and by noting that $\varepsilon ^{n} C(s_0)^n$ is a summable sequence, for $\varepsilon$ small enough, we prove the thesis. $\quad \square$

## Footnotes

1 Since $\omega$ is diophantine we can replace the time variable with angles $\varphi \in \mathbb {T}^{\nu }$. The time dependence is recovered by setting $\varphi =\omega t$.

2 The function $\varepsilon \overline{v}$ represents a torus supporting a quasi-periodic motion which is invariant for the system (5.1) with $P=0$, namely it is the approximate solution from which we bifurcate.

3 Notice that $\xi =\xi (\omega )$, recall (4.11).

4 Whenever it is not strictly necessary, we shall drop the dependence on $i_{\delta }$.
